# One Stone Two Birds: Novel Fluorescent Probe Chemistry That Detects Redox Stress in Cell Culture

**DOI:** 10.1021/acs.analchem.6c02835

**Published:** 2026-08-11

**Authors:** Shubham Bansal, Muskan Gori, Binghe Wang

**Affiliations:** Department of Chemistry and Center for Diagnostics and Therapeutics, 1373 Georgia State University, Atlanta, Georgia 30303, United States

## Abstract

Redox homeostasis is a critical part of physiological processes. Disruption of such homeostasis is associated with various pathological conditions. Tools for studying cellular redox states are important for understanding various biological mechanisms and for the targeted delivery of drugs and/or imaging agents. Along this line, there have been reaction-based fluorescent probes capable of detecting either oxidative stress or hypoxia. However, tools are not available to detect dynamic redox changes in either direction. Toward that end, we describe chemistry that allows for detecting both reduction and oxidation changes using a probe redox pair, moving toward monitoring dynamic redox processes. Embedded in this new approach is also new chemistry for fluorophore activation through the selective reduction of a sulfone group to a thioether under severe hypoxia, complementing the widely used nitro or *N*-oxide reduction approaches. We demonstrate the feasibility through solution-phase studies and cell-culture work in two cell lines: cancer cells and macrophages. One optimized redox probe pair was studied for its response to both induction of highly reactive oxygen species (hROS) production by turning off the fluorescence and severe hypoxia with fluorescence turn-on. Overall, this approach sets a new direction in designing redox-sensitive tools.

## Graphical abstract

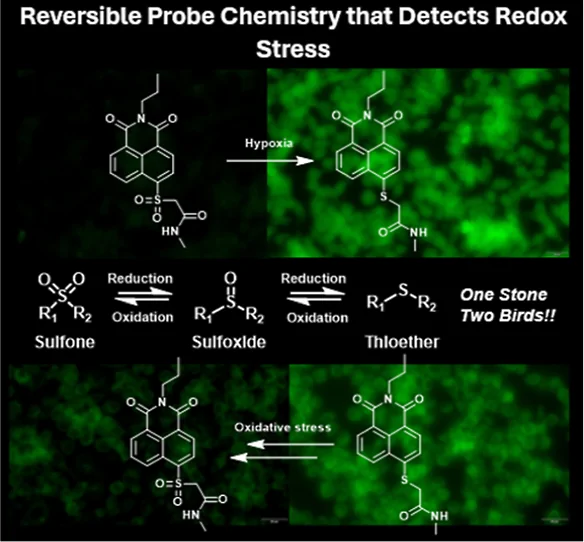

Under normal conditions, cells maintain redox homeostasis.^1,2^ Either oxidative stress^3^ or hypoxia^4^ can be a cause for disruption of such a balance. Oxidative stress is reflected in the overproduction of reactive oxygen species (ROS)^5^ and is associated with conditions such as cancer,^6–8^ viral infections,^9–12^ cardiovascular diseases,^13,14^ neurological disorders,^15,16^ chronic inflammation,^17,18^ and diabetes^19,20^ among others. ROS is a group of species with significant variations in reactivity and concentration.^21^ Among all the ROS, hydrogen peroxide (H_2_O_2_) and hypochlorite (OCl^–^) are two known species to accumulate under various pathological conditions.^21^ Although there is a need to look into the experimental details when discussing their concentrations for practical applications,^22–24^ H_2_O_2_ concentration has been reported to be as high as 610 μM under certain pathophysiological conditions.^21^ In contrast, basal concentrations have been reported to be around 3 μM in human plasma.^21^ For OCl^–^, the concentration differences between physiological and pathological states are in a similar range. Specifically, the concentration of OCl^–^ has been reported to be about 39 μM in unstimulated neutrophils and about 398 μM upon stimulation.^25^ Such differences in ROS concentration have been exploited in imaging and drug delivery applications.^26–33^ It should be noted that H_2_O_2_ is ubiquitous and tends to have high background and issues of concentration fluctuation under normal physiological conditions.^34–36^ On the other hand, highly ROS (hROS) tend to be much more correlated with pathological conditions. Though there is no strict definition of hROS, anything above H_2_O_2_ reactivity is generally considered hROS. As an example, OCl^–^ is only produced by immune cells in response to infection,^37^ inflammation,^38^ or in malignancy such as acute myeloid leukemia^39^ and certain stages of chronic myeloid leukemia (CML).^40,41^ Infiltration of malignant tumors or inflamed sites by neutrophils and macrophages tends to elevate OCl^–^ levels at such locations. Therefore, the ability to detect hROS affords the chance to specifically study pathologically relevant ROS.

On the flip side of the issue is hypoxia, which can be the result of various pathological conditions,^42–44^ including malignant solid tumors,^45–47^ cardiovascular disorders,^48–50^ neurological disorders,^51^ and respiratory disorders^52,53^ among others. Along the same line, tumor hypoxia has been widely studied as it often leads to unfavorable prognoses for patients with few treatment options.^54^ Tumor hypoxia is an oxygen-deficient state and a key feature of malignant tumors developed from the inadequate and irregular vascularization of rapidly proliferating cancer cells.^47,55–57^ Such changes also result in the alteration of cellular metabolism.^58,59^ As a result, hypoxic regions are often embedded deep in the tumor, having an oxygen level less than 2%. In the most severe cases, the oxygen level is at ∼0.1%, which is known as radiobiological hypoxia.^60,61^ Hypoxia causes tumor cells to have their own microenvironment, including low extracellular pH, high interstitial fluid pressure, glucose deficiency, and an increase in the concentration of extracellular lactate.^58,59^ Often radiotherapy is ineffective against hypoxic tissue due to the essential nature of oxygen as a sensitizer in radiotherapy.^62–66^ Furthermore, chemotherapies are also ineffective in tumors under hypoxia, as hypoxic cells enter into hypoxia-induced quiescence while chemotherapies target actively proliferating cells.^62–66^ Often, surgical procedures are the only option for hypoxic tissues because of difficulties in targeting hypoxic tissues.^67–69^ For all these reasons, there is also a widespread interest in detecting hypoxic regions for studying basic ROS biology as well as diagnostic^70–72^ and drug delivery^73,74^ purposes.

Multiple approaches have been developed for the detection of either oxidative stress or hypoxia/reductive conditions.^27,30–32,70–72^ For sensing oxidative stress, primarily oxidation of boronic acid/ester and chalcogens (group 16 elements) have been used (Figure 1E,F).^27,30–32^ For sensing reductive conditions, four types of reduction reactions are commonly used in fluorescent probe design: reduction of a nitro to an amino group,^75,76^ *N*-oxide to an amino group,^77,78^ and quinone to dihydroquinone^71,79^ as well as reductive cleavage of an azo moiety (Figure 1A–D).^80,81^ Most such reduction reactions are not reversible in practical terms, except for the reduction of quinone to dihydroquinone. However, quinones are Michael acceptors and can react with protein and DNA molecules,^82^ leading to covalent modifications.^82^ Further, the azo group can be reduced by the gut microbiome, specifically by azoreductases;^83^ *N*-oxides participate in several chemical reactions;^84^ nitro reduction-based approaches can be activated in mildly hypoxic or normoxic conditions, leading to off-target effects.^85^ Furthermore, the nitro group is considered a toxicophore,^86^ and nitro reduction-based approaches have safety and efficacy concerns.^87^ These examples indicate the need for new chemistry to detect hypoxia, especially in malignant tumors. Furthermore, tumor hypoxia may vary greatly within the same patients.^88,89^ Often during the progression of disease, malignant tumors generally become increasingly more heterogeneous.^90^ Due to this heterogeneity, the tumor tends to include a diverse collection of cells with distinct molecular signatures.^90^ Among this heterogeneity, redox state can vary significantly within the same and/or among different tumors.^90,91^ For example, human breast cancer tissues have been reported to have a more reductive environment than the benign adjacent breast tissues.^91^ On the other hand, lung cancer tissues have been reported to have a higher oxidative environment as compared to the benign lung tissue.^92^ To detect this heterogeneity, we are interested in developing detection chemistry that affords the ability to report redox changes in both directions. Herein, we report a new type of activation chemistry for selective reduction of a sulfone group to thioether under severe hypoxia while providing excellent stability under normoxia and a fluorescent reporting event (Figure 1). In the reverse, hROS such as hypochlorite can oxidize thioether to sulfoxide and sulfone. Our approach provides a new platform for redox sensing. Below we describe our findings in detail.

**Figure 1:**
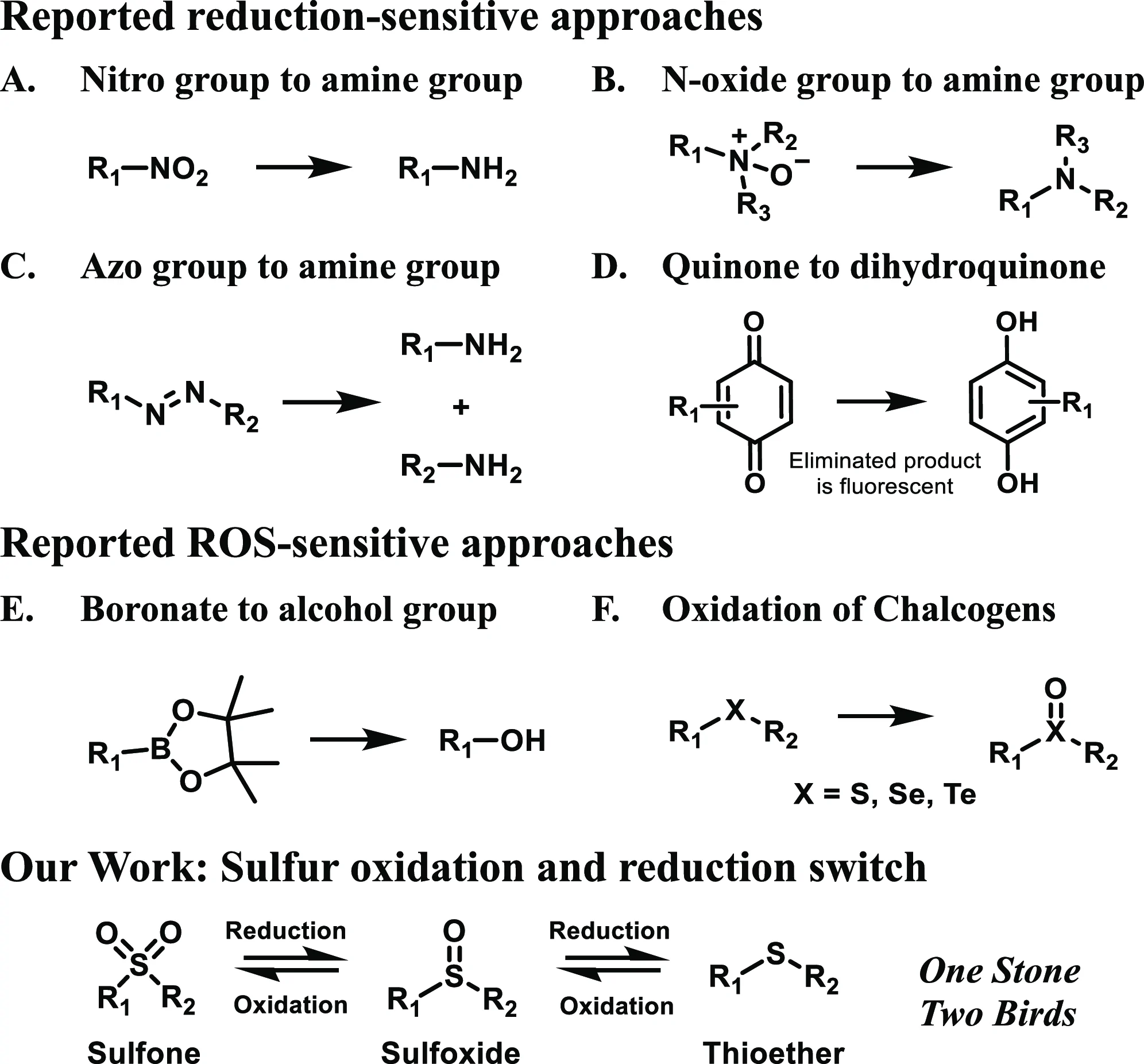
Chemistry of reaction-based redox-sensitive approaches. (A) Reduction of a nitro group to an amino group. (B) Reduction of an *N*-oxide group to an amino group. (C) Reduction of an azo group to an amino group. (D) Reduction of quinone to dihydroquinone. (E) Conversion of a boronate ester to an alcohol. (F) Oxidation of chalcogens (group 16 elements).

## Experimental Section

The general material and methods and the rest of the experimental section are in the Supporting Information.

### Reduction in HeLa Cells under Hypoxia

First, a 10 mM stock solution of compound was prepared in DMF. Then, a working solution of 20 μM was prepared by adding 20 μL of 10 mM stock solution to 10 mL of media. Subsequently, a 6-well plate was prepared a day before the experiment to achieve 80% cell confluency. On the day of the experiment, media was removed from each well of the 6-well plate and washed with 1 mL of cold PBS. After that, 2 mL of the designated working solution was added to the designated wells, and the plate was then incubated under normoxia or hypoxia at 37 °C. At the designated time, the media was aspirated, and cells were washed with 3 × 3 mL of cold PBS and then incubated with 200 μL Trypsin at 37 °C until the cells detached from the plate. Then, 1 mL of cold PBS was added to each well, and the sample was analyzed by using a flow cytometer.

### Sensing Hypoxia in RAW264.7 Cells

First, a 10 mM stock solution of probe was prepared in DMF. Then, a working solution of 10 μM was prepared by adding 10 μL of 10 mM stock solution to 10 mL of media. Subsequently, 60 mm Petri dishes were prepared a day before the experiment to achieve 80% cell confluency. On the day of the experiment, media was removed from the Petri dish and washed with 1 mL of cold PBS. After that, 2 mL of the designated working solution was added to the designated wells, and the plates were then incubated under normoxia or hypoxia at 37 °C. At the designated time, the media was aspirated, and cells were washed with 3 × 1 mL of Fluorobrite DMEM, and then the samples were analyzed by using a fluorescence microscope.

### Sensing Oxidative Stress in RAW264.7 Cells

First, a 10 mM stock solution of probe and PMA was prepared in DMF. Then, a working solution of 10 μM probe was prepared by adding 10 μL of 10 mM stock solution to 10 mL of media. Simultaneously, a working solution of 10 μM probe and 2 μM PMA was prepared by adding 10 μL of 10 mM probe solution and 2 μL of 10 mM PMA solution to 10 mL of media. Subsequently, 60 mm Petri dishes were prepared a day before the experiment to achieve 80% cell confluency. On the day of the experiment, media was removed from the Petri dish and washed with 1 mL of cold PBS. After that, 2 mL of the designated working solution was added to the designated wells, and the plates were then incubated under normoxia at 37 °C. At the designated time, the media was aspirated, and cells were washed with 3 × 1 mL of Fluorobrite DMEM, and then the samples were analyzed by using a fluorescence microscope.

### Sensing Reversibility in RAW264.7 Cells

First, a 10 mM stock solution of probe and PMA was prepared in DMF. Simultaneously, a 10 mM stock solution of Rhodamine B was prepared in water. Then, a working solution of 10 μM probe or rhodamine B was prepared by adding 10 μL of 10 mM stock solution to 10 mL of media. Subsequently, 96-well plates were prepared a day before the experiment to achieve 80% cell confluency. On the day of the experiment, media was removed from the Petri dish and washed with 0.1 mL of cold PBS per well. After that, 0.1 mL of the designated working probe solution was added to the designated wells, and 0.1 mL of 10 μM rhodamine B was added to the wells in the absence of cells. Then, the plates were incubated under hypoxia at 37 °C. At the designated time, the samples were analyzed by using a plate reader. Then, PMA was added to the designated wells to have a final concentration of 2 μM, and the plates were incubated under normoxia at 37 °C. At the designated time, the samples were analyzed by using a plate reader.

## Results and Discussion

### The Design

In designing the overall approach, we were interested in functional group chemistry that offers the following features: (1) general chemical stability, (2) lack of problematic features such as a Michael acceptor, a hot electrophile, or a known toxicophore, (3) being devoid of terminal/irreversible redox conversion under normal pathophysiological conditions, (4) sensitivity to redox chemistry under near-pathophysiological conditions, and (5) known ability to influence spectroscopic properties of adjacent chromophore. For all these reasons, we turned to a thioether conjugated to an aromatic system, which is known to impart fluorescence changes upon oxidation.^93^ For the initial studies, we chose naphthalimide because of its strong fluorescence when directly connected to a thioether and weak fluorescence when directly connected to a sulfoxide group.^94^ We reasoned that the sulfone analogue should also have different fluorescence properties from that of the thioether because of the lack of lone-pair electrons on the sulfur atom. As a result, we chose the naphthalimide core to have a fluorescence on–off system via thioether-sulfoxide/sulfone conversions. It should be noted that sulfoxide has been widely studied and is known to be reduced by enzymes, including methionine sulfoxide reductase A.^95,96^ The sulfone moiety has not been explored for reduction-based fluorescent detection. For example, sulindac sulfone has been known to be stable in liver microsomes,^97^ which is highly desired to achieve selective activation at the desired site. Hence, we chose to examine sulfone reduction under extreme hypoxia. Sulfones are often considered resistant to further oxidation and hence not prone to irreversible side reactions under oxidative stress.^98^ Therefore, we designed four sets of molecules containing a naphthalimide core conjugated with a sulfone moiety with varying R_1_ (Figure 2). For now, R_2_ is fixed and reserved for later modification if needed, including conjugation with a targeting moiety or a solubility enhancer, among others (Figure 2). This approach is intended for activation by pathologically relevant, strongly reductive conditions of hypoxic environments. This aspect is unique in the sense that most literature is based on reducing quinones,^71,79^ a nitro group,^75,76^ *N*-oxides,^77,78^ or an azo group,^80,81^ which tend to be achievable under mildly reductive conditions or under normal physiological conditions.

**Figure 2:**
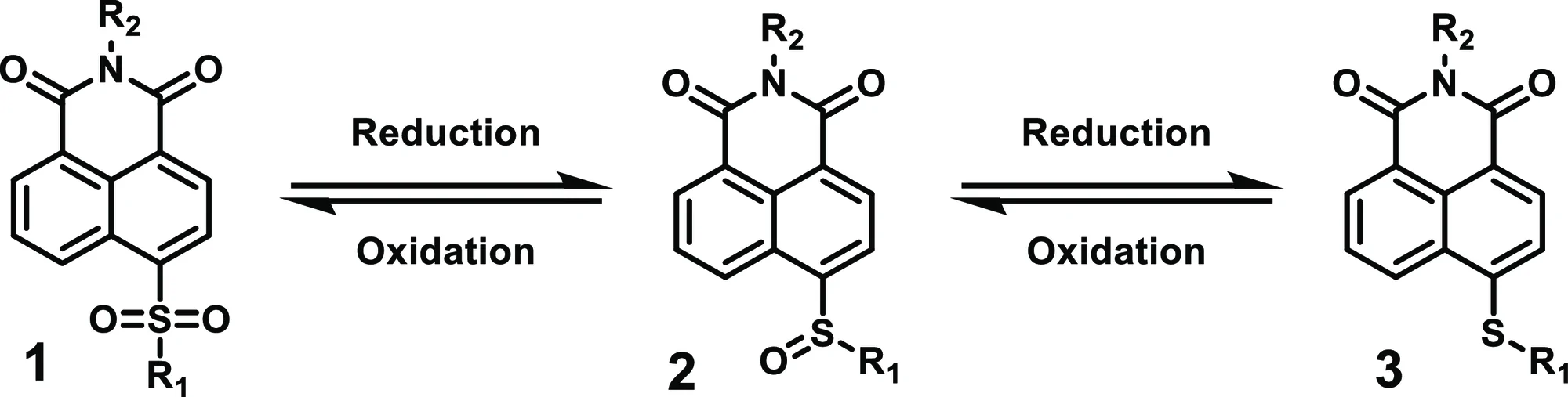
General design of probes.

### Synthesis

Synthesis of the probes is straightforward, as shown in Scheme 1, with all of the reactions being extensively explored.^99,100^ Briefly, 4-bromo-1,8-naphthalic anhydride **4** was reacted with thiols **5a**–**d** in the presence of K_2_CO_3_ to yield compounds **6a**–**d**, followed by reaction with an amine (**7a**–**d**) to yield probes **3a**–**d** (Scheme 1). Probes **3a**–**d** were then reacted with *m*-CPBA to yield the corresponding sulfoxide or sulfone. Specifically, thioethers **3a**–**d** were each reacted with 3 eq. of *m*-CPBA to yield sulfones **1a**–**d** and 0.9 eq. of *m*-CPBA to yield sulfoxides **2a**–**d**. A reported probe **1d** was synthesized as a positive control in GSH reactivity studies (see a later section for details).^101^ Next, we determined their spectroscopic properties in order to select a probe with desired spectral properties for cellular studies.

**Scheme 1:**
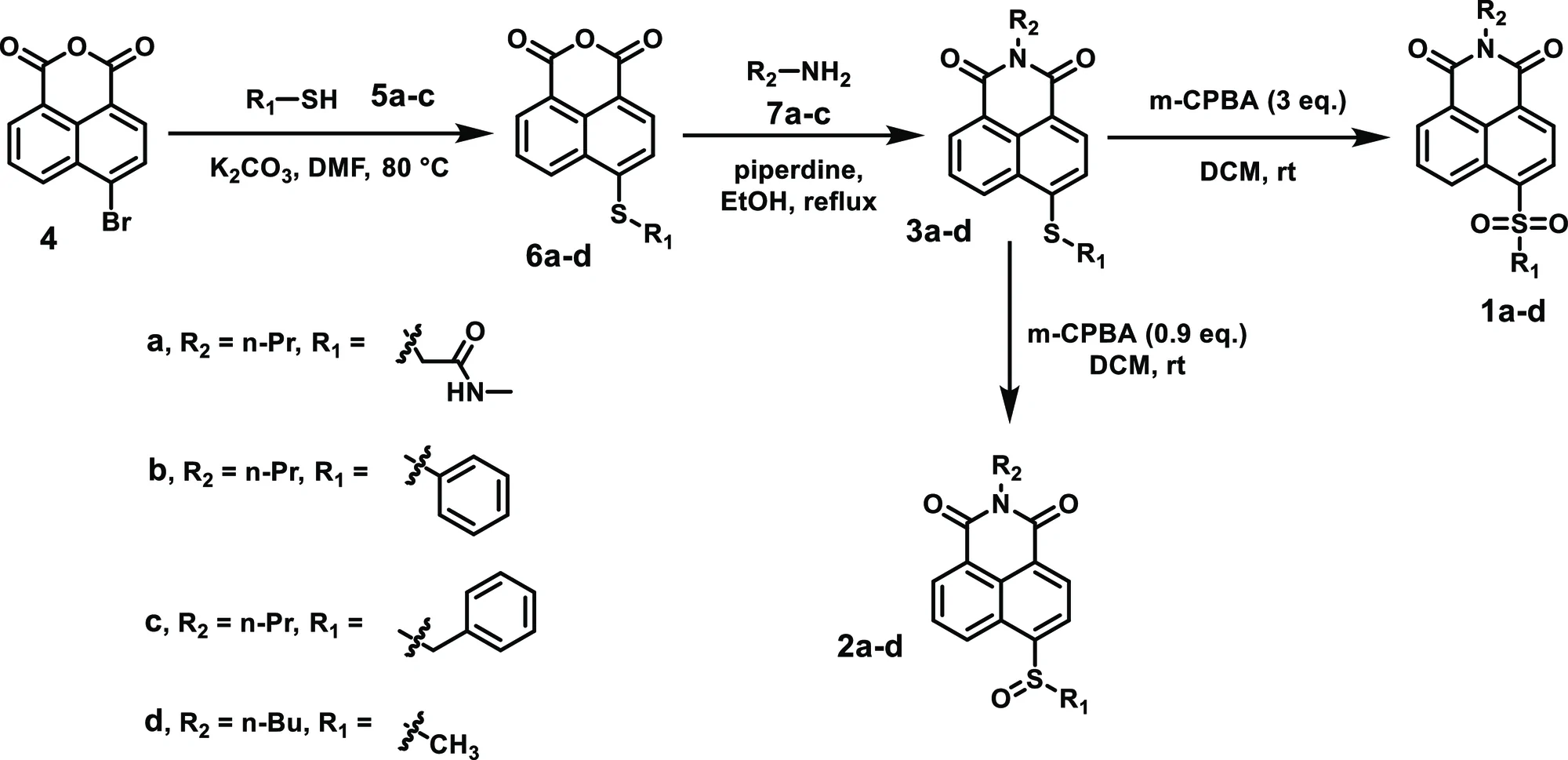
Synthesis Scheme of Probes

### Spectroscopic Properties

We started by determining the UV–vis and fluorescence properties for each of the synthesized compounds, including thioether **3a**–**c**, sulfoxide **2a**–**c**, and sulfone **1a**–**c**. Briefly, a 10 mM stock solution of each compound was prepared in DMF and then used to prepare a 50 μM solution in PBS/ACN (1:1) at pH 7.4 for spectral measurement. As expected, all three series of thioethers showed significant UV–vis changes upon oxidation to sulfoxide and then sulfone (Figure 3). Specifically, thioethers **3a**–**c** showed strong fluorescence with a Stokes shift of more than 100 nm (Figure 3). The fluorescence quantum yields (Φ_F_) of **3a, 3b,** and **3c** were determined to be 0.21, 0.09, and 0.10, respectively (Table S1). On the other hand, both sulfoxides **2** *a* **/2c** and sulfones **1** *a* **/1c** showed no fluorescence. In contrast, sulfone **1b** and sulfoxide **2b** showed some background fluorescence compared to thioether **3b** (Figures 3 and S1). Therefore, for subsequent studies, we used the **1** *a* **/2a/3a** and **1** *c* **/2c/3c** sets because of their desirable features of fluorescence on–off switch upon redox conversions.

**Figure 3:**
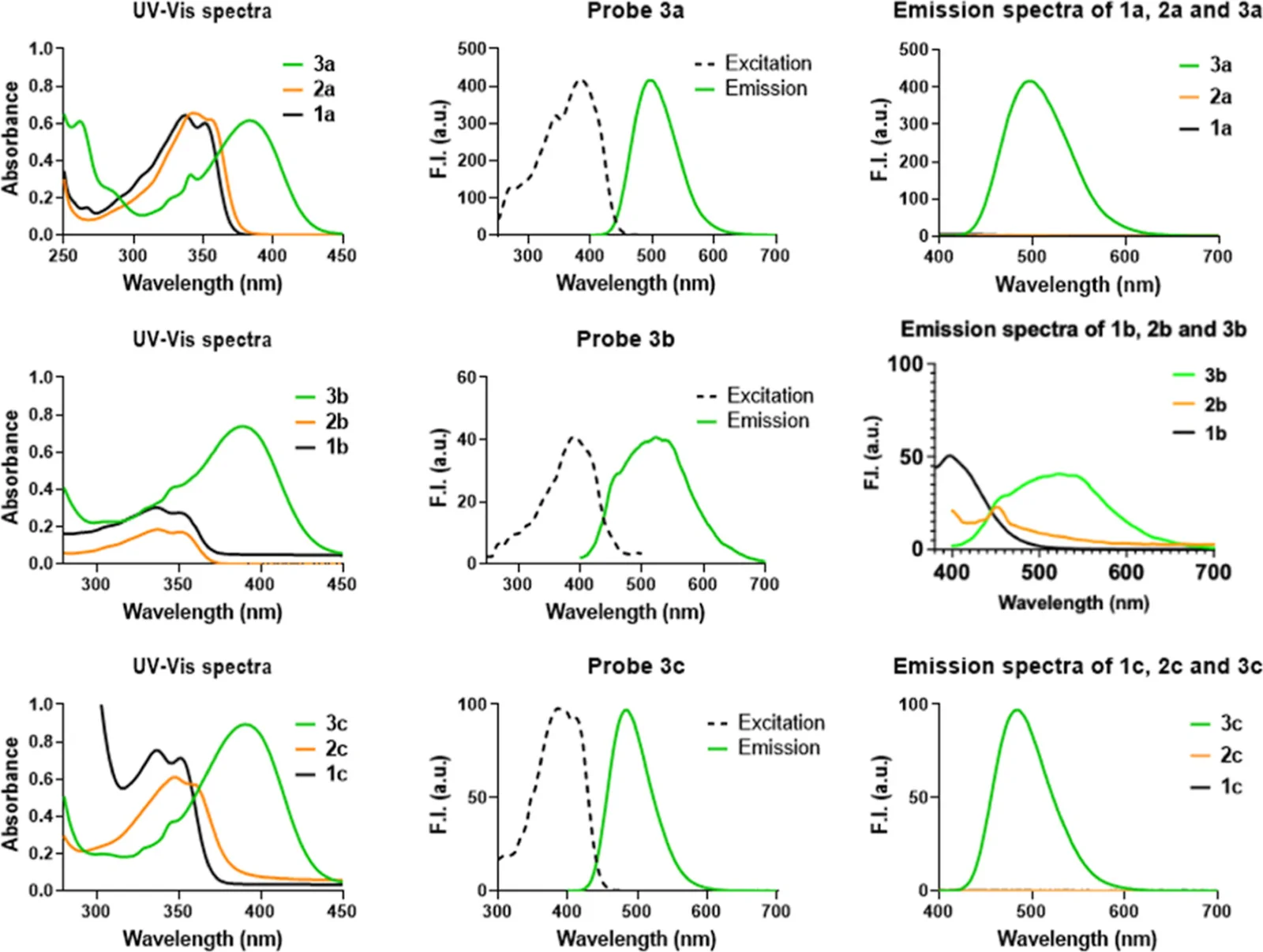
UV–vis and fluorescence spectra of each probe. Emission spectra of **1b** and **3b** were recorded by exciting at 360 and 400 nm, respectively.

### Probe Reaction with ROS

As discussed before, ROS is a group of species with varying reactivity.^21^ Among all the ROS, only hydrogen peroxide (H_2_O_2_) and hypochlorite (OCl^–^) are stable enough to accumulate.^21^ These ROS species react with thioethers with a significant difference in reaction rate. Specifically, for the reaction with the thioether of methionine, the second order rate constant is 2 × 10^–2^ M^–1^ s^–1^ with H_2_O_2_ ^102^ and 3.7 × 10^8^ M^–1^ s^–1^ with hypochlorite.^103,104^ This 10-orders-of-magnitude difference indicates a binary yes-or-no switch in kinetic terms in response to hypochlorite, but not H_2_O_2._ Further, thioether oxidation is almost instantaneous (<1 ms) in a practical sense by NaOCl at single digit micromolar concentrations.^105^ Nevertheless, we tested the reactivity of our thioether probe with four forms of biologically relevant ROS, including H_2_O_2_, NaOCl, ONOO^–^, and ^•^OH. Specifically, we incubated 50 μM of probe **3c** with 50 μM ROS in PBS/ACN (1:1) at pH 7.4 (except for ^•^OH, which was prepared in situ using the Fenton reaction with 1 mM H_2_O_2_ and 0.1 mM Fe­(II) (See later for details). Among the four reactions, we observed probe turn-off only with NaOCl (Figure 4A–C). These results are in-line with literature reports and our previously reported study.^22,105,106^ We should note that under the same conditions, ONOO^–^ and ^•^OH were able to turn on the fluorescence of probes such as boronate **4** or APF, which are designed to detect the respective species (Figure 4D–G), serving as positive controls. Overall, the results indicate that the thioether probe can be rapidly and selectively activated by NaOCl and can be used in cellular studies.

**Figure 4:**
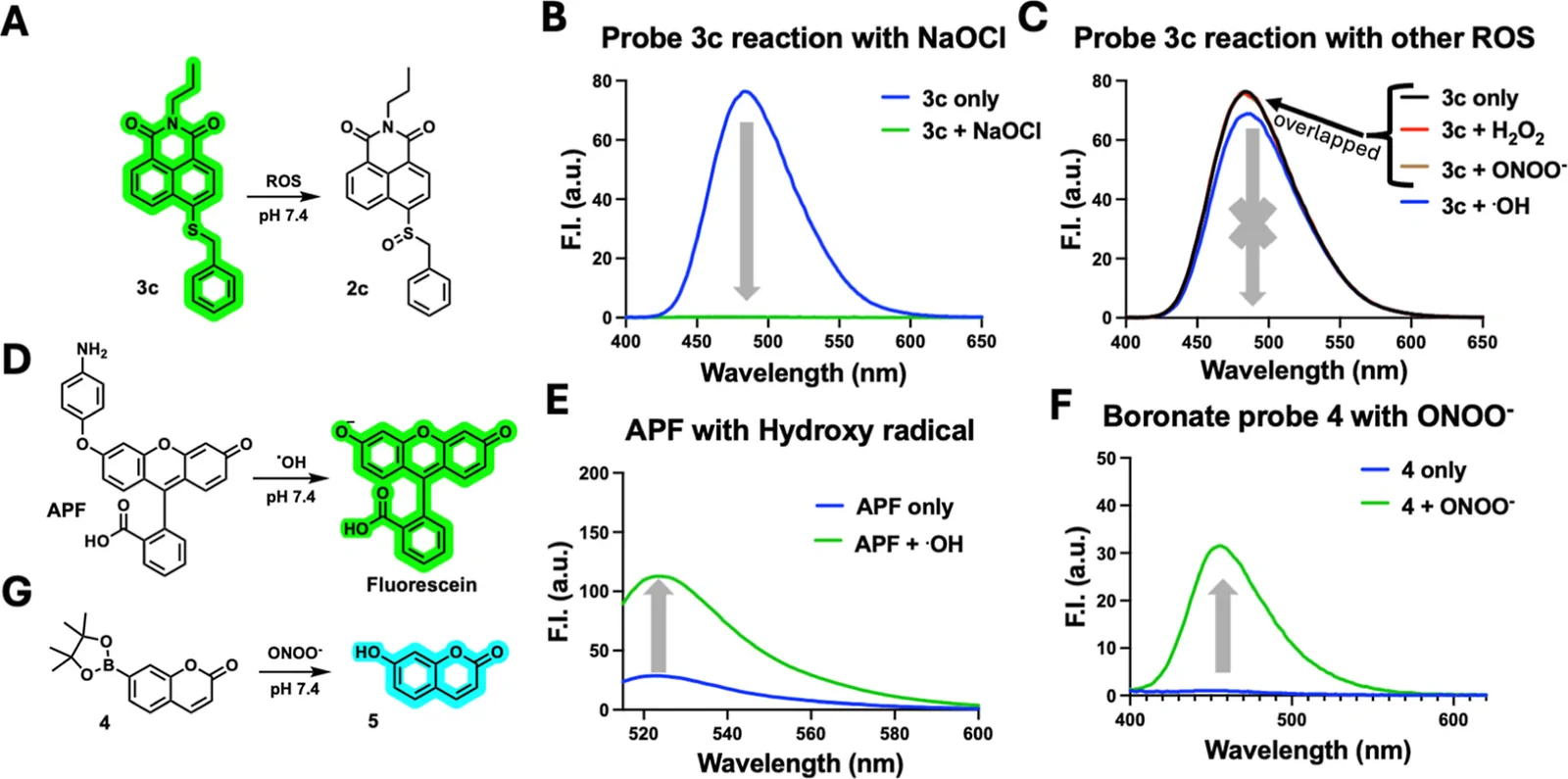
(A) Chemical reaction of thioether **3c** oxidation by NaOCl. (B) Fluorescence spectra of 50 μM of **3c** with 50 μM NaOCl in PBS/ACN (1:1) at pH 7.4; (C) fluorescence spectra of 50 μM of **3c** with 50 μM H_2_O_2_, 50 μM ONOO^–^, and ^•^OH (1 mM H_2_O_2_ and 100 μM Fe^2+^) in PBS/ACN (1:1) at pH 7.4; (D) chemical reaction of **APF** reaction with ^•^OH. (E) Fluorescence spectra of 10 μM **APF** with ^•^OH (1 mM H_2_O_2_ and 100 μM Fe^2+^) in PBS/ACN (1:1) at pH 7.4; (F) fluorescence spectra of 50 μM boronate probe **4** with 50 μM ONOO^–^ in PBS/ACN (1:1) at pH 7.4. (G) Chemical reaction of boronate probe **4** with ONOO^–^.

At this point, it is important to do a kinetic analysis to understand the negative responses of **3c** to other forms of ROS studied (Figure 4C). The negative results with H_2_O_2_ are easy to understand. With a rate constant of 2 × 10^–2^ M^–1^ s^–1^,^102^ the *t*_1/2_ is about 12 days at 50 μM each, i.e., the reaction is too slow to be meaningful for the experiments conducted. However, the same cannot be said about the other two: ONOO^–^ and ^•^OH. We use ONOO^–^ as an example for discussion. First, ONOOH has a p*K*_a_ of about 7.4, and two species, ONOOH and ONOO^–^, have significantly different reaction kinetics.^103,107^ The second order rate constants of the reaction of ONOOH and ONOO^–^ with methionine have been reported to be 2 × 10^3^ M^–1^ s^–1^ and 2 × 10^–1^ M^–1^ s^–1^, respectively.^103,107^ The 2 × 10^3^ M^–1^ s^–1^ reaction rate indicates an estimated *t*_1/2_ of 10 s at 50 μM each. Then why the negative results with ONOO^–^. The answer is in its stability. Degradation of ONOO^–^ has a first-order apparent rate constant of about 0.9–1.2 s^–1^,^105,108^ giving a *t*_1/2_ of 0.6–0.8 s, which is much shorter than the thioether oxidation half-life, therefore creating a kinetic barrier for probe oxidation. The issue with ^•^OH is similar. Though it is expected to react with thioethers with a second-order rate constant of 7 × 10^9^ M^–1^ s^–1^,^109^ ^•^OH also degrades fast, with the commonly reported *t*_1/2_ being in the range of μs-ns scale in an aqueous environment due to radical consumption.^110–112^ Further, ^•^OH has high and nondiscriminative reactivity with essentially all organic molecules with a similar reaction rate.^113^ Therefore, the lack of response of **3c** to ^•^OH is not due to “reactivity” per se, but the unstable nature of ^•^OH. Then we need to answer why there are positive results with APF. The second-order rate constant for the reaction between APF and ^•^OH has been reported as 2.9 × 10^11^ M^–1^ s^–1^,^114^ which is near diffusion control and about 40-fold higher than that of thioether, allowing for ^•^OH detection. Therefore, detection in a biologically relevant environment needs to consider kinetic and other interference factors. Along this line, we also need to note that superoxide produced in cellular processes is not expected to be an interference because of its known slow reaction (*k*: 0.3 M^–1^ s^–1^)^103^ and fast dismutation/degradation (*t*_1/2_ in the range of μs).^115^ Overall, our thioether-based probe can be rapidly activated selectively by OCl^–^ in the presence of other hROS such as ^•^OH and ONOO^–^.

### Sensing Cellular Oxidative Stress

To study both oxidative stress and hypoxia, we needed a cell line that can produce sufficiently high levels of hROS such as OCl^–^, among others, upon stimulation. It is important to note that not all cell lines produce a sufficient level of hROS upon stimulation.^24^ Therefore, as a first step, we studied hROS production in different cell lines using a well-established commercial probe DCFH-DA, which is the diester version of DCFH and capable of cellular penetration.^116–118^ Subsequent hydrolysis of the ester groups of DCFH-DA would lead to DCFH (Figure 5), which is not readily cell membrane-permeable and thus is trapped intracellularly.^119^ DCFH reacts rapidly with hypochlorite (second-order rate constant: 580 ± 18 M^–1^ s^–1^)^22^ or a species with similar reactivity to generate a fluorescent product (Figure 5).^120^ On the other hand, DCFH does not react with H_2_O_2_.^22,23,31^ Therefore, we used DCFH-DA to detect hROS with reactivity similar to or higher than that of hypochlorite.^23^

**Figure 5:**
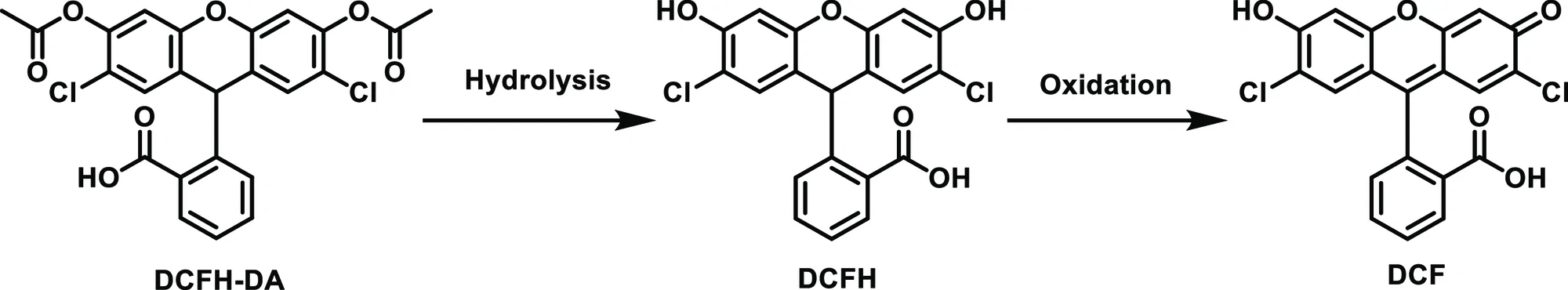
DCFH-DA activation chemistry.

We chose a total of six cell lines, including three cancer cell lines (HeLa, RS4; 11, 4T1), two normal cell lines (HEK293T, H9C2), and a macrophage cell line (RAW264.7) to study ROS production upon stimulation with LPS and/or PMA. Briefly, we incubated the cells with 10 μM DCFH-DA with and without a stimulant (2 μg/μL LPS or 2 μM PMA) for 30 min before fluorescence studies. We found that only RAW264.7 cells showed a significant increase in hROS production upon stimulation with either stimulant (Figure 6). This was expected for several reasons. First, macrophages are known to express myeloperoxidase (MPO) and thus produce hypochlorite, a stable hROS.^121^ Second, LPS is recognized by cell membrane receptors including TLR4, MD2, and CD14,^122,123^ and their expression level can change depending on cell line, leading to differences in ROS production. Along this line, immune cells such as macrophages are known to express these receptors and respond to LPS.^122,123^ Third, the level of hROS is expected to be low for non-MPO-expressing cells even if stimulated. This is because of the short half-life of other hROS such as superoxide anion (O_2_^–.^, short-lived), hydroxyl radicals (OH^•^, react with almost everything in the cell), peroxynitrite (ONOO^–^, short-lived), and singlet oxygen species (^1^O_2_, short-lived), among other forms.^124,125^ As further validation, we wanted to see if prolonged exposure to a known stimulant would increase hROS production. Thus, HeLa and 4T1 cells were incubated with LPS or PMA for 4 h. Neither cell types showed a measurable level of hROS production (Figure S2). Overall, only RAW264.7 (macrophage) cells showed elevated hROS production with LPS or PMA stimulation. Again, such results are in line with the lack of MPO expression and thus hypochlorite production by solid tumor cells and the lack of accumulation of other hROS due to their short half-life.^126–130^ Though infiltration by neutrophils and macrophages can lead to detectable levels of OCl^–^ in solid tumors (or in inflamed tissues) in vivo, the situation with cell culture is completely different.^126–130^ Subsequently, we used the RAW264.7 cells for studying whether probes **3a** and **3c** can sense changes in the redox state.

**Figure 6:**
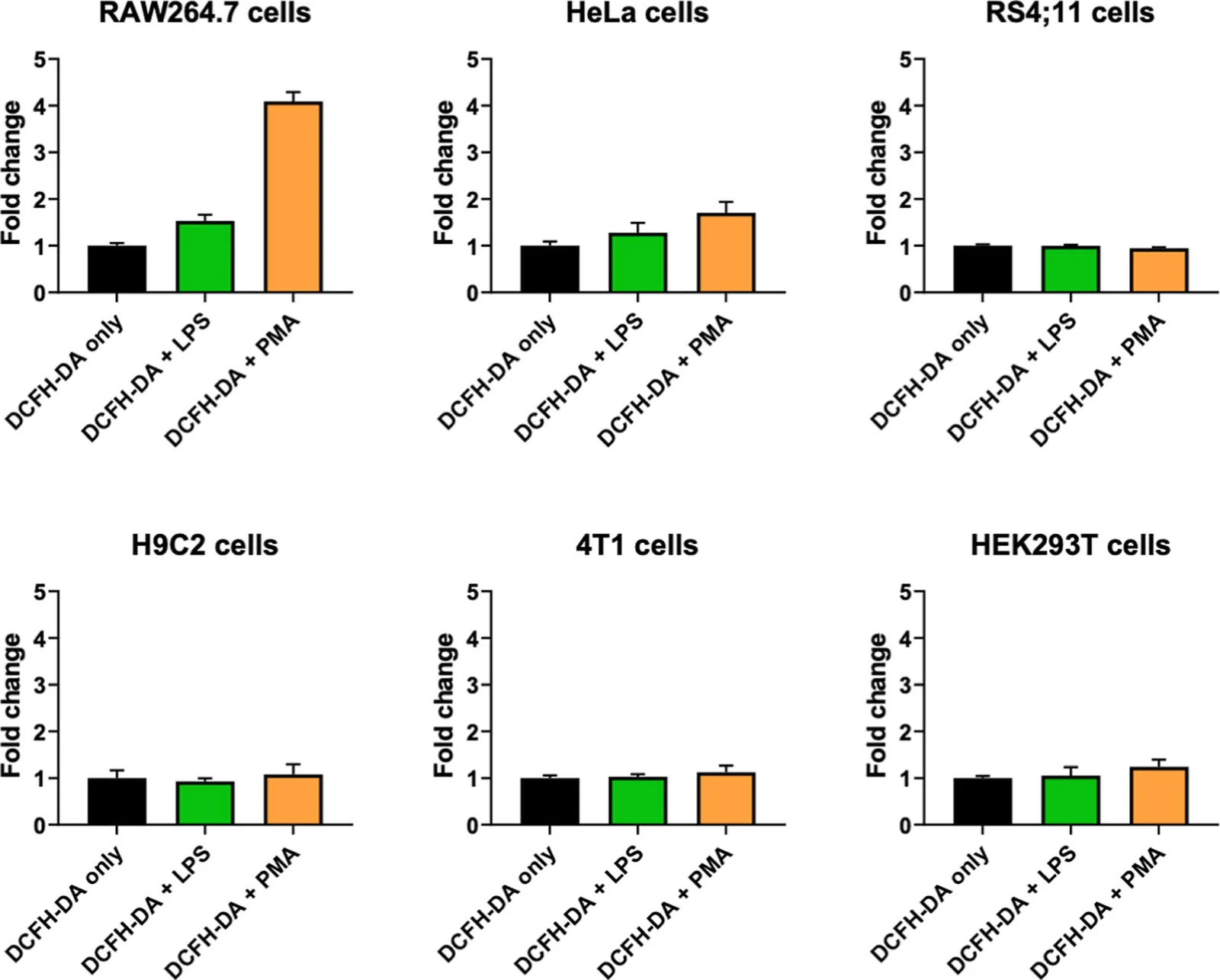
ROS detection in various cells with LPS or PMA stimulation after 30 min incubation.

Briefly, we incubated 10 μM of probe **3a** or **3c** in the presence and absence of 2 μM PMA with RAW264.7 cells and found a significant fluorescence decrease upon PMA stimulation (Figure 7), as expected. These results are in line with the results from the DCFH-DA probe (Figure 6). Subsequently, we examined the ability to sense hypoxia via reduction of sulfones **1a,c**.

**Figure 7:**
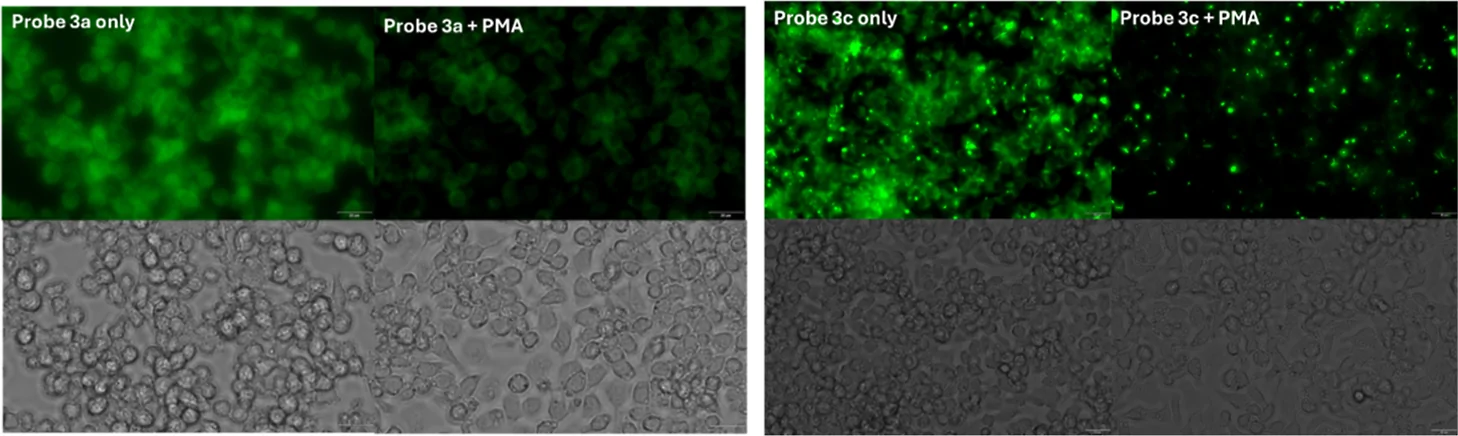
Sensing oxidative stress in RAW264.7 cells using probe **3a** or **3b**.

### Probe Reduction under Anaerobic Conditions in RAW264.7 Cells

As discussed before, the interest and need are in detecting redox shifts in both directions. This means the probe must possess the characteristics of responding to either hypoxia or oxidative stress. Again, the design principle relies on redox conversions between thioether and sulfone/sulfoxide. Between these two reactions, thioether oxidation under oxidative stress has been extensively studied and reported to work in live cells and animal models.^93^ Indeed, we also observed similar results of thioether oxidation under oxidative stress (Figure 7). Subsequently, we focused on detecting hypoxia using sulfone reduction chemistry. We used the same cell line, RAW264.7, as in the oxidative stress studies to detect hypoxia using the same probe sets **1** *a* **/2a/3a** and **1** *c* **/2c/3c**. Initially, we used fluorescent microscopy to determine the visual contrast in hypoxia vs normoxia conditions. Then, we used flow cytometry to quantify the results (see later sections for details). Briefly, we incubated 10 μM of the sulfone probe **1a** or **1c** with RAW264.7 cells under anaerobic conditions and found probe turn-on within 1 h of incubation (Figure 8), indicating sulfone reduction under hypoxia. Furthermore, this is the first time that such probe activation chemistry is based on sulfone reduction under hypoxia conditions; it provides new opportunities in sensing hypoxia or a reductive cellular environment.

**Figure 8:**
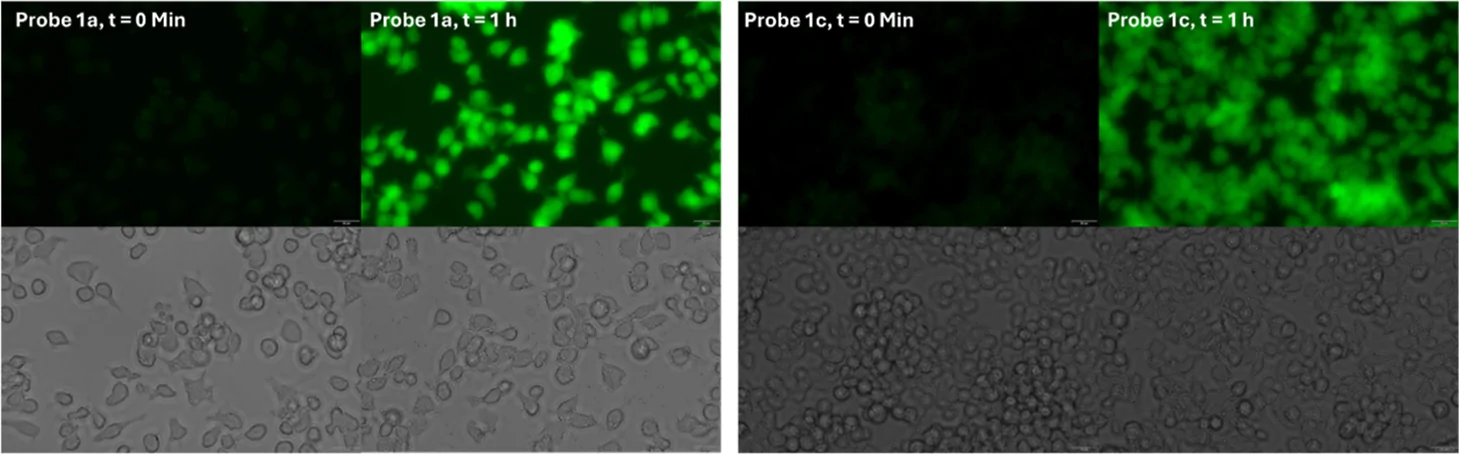
Sensing hypoxia in RAW264.7 cells using probe **1a** or **1c.**

After establishing the ability for RAW264.7 cells to turn on the fluorescence of probe **1c** under hypoxia (Figure 8), we designed experiments to compare probe turn-on under normoxia and hypoxia. Briefly, we incubated 10 μM of the sulfone probe **1c** with RAW264.7 cells under anaerobic and normal conditions for 3 h and found a significant difference in the probe turn-on under hypoxia (Figure 9), indicating the feasibility of a reduction-sensitive probe as designed. Given the new chemistry, it is crucial to understand the following factors: first, whether the probe can reversibly detect both oxidative stress and hypoxia within the same cells; second, whether the probe can detect hypoxia in cancer cells; third, the kinetics of hypoxia sensing with respect to changes in the partial pressure of oxygen. As an initial attempt, we designed an experiment to show reversibility in RAW264.7 cells. Specifically, we used the same batch of cells to first examine the ability to turn it on under anaerobic conditions and then the ability to turn it off under oxidative stress. Briefly, we incubated 10 μM of probe **1a** or **1c** with RAW264.7 cells under anaerobic conditions for 1 h and found the probe turned on under hypoxia as expected (Figure S3). Afterward, the same cells were incubated with 2 μM PMA under normoxia, and fluorescence was recorded after 1 h (Figure S3). It was found that the fluorescence intensity increased after 1 h of PMA stimulation (Figure S3). These results are unexpected and indicate that further reductive stress even after emerging from hypoxia upon PMA stimulation. Specifically, such results are quite the opposite of the general behaviors of RAW264.7 cells without first experiencing hypoxia.^21^ It should be noted that the tumor microenvironment has been reported to change macrophage phenotypes between M1/M2.^131^ Therefore, hypoxia could alter the cellular biology leading to such results. Indeed, severe hypoxia has been reported to decrease the PMA-stimulated ROS production as compared to normoxia in leukocytes.^132^ Our results are in line with such reports and indicate that this probe chemistry could help to understand those cellular changes. However, understanding this biological process is expected to be a complex undertaking beyond the scope of this probe chemistry study. Nevertheless, it is crucial to understand the probe activation in a cancer cell line under various partial pressures of oxygen. Therefore, in this study, we focused on studying the next section.

**Figure 9:**
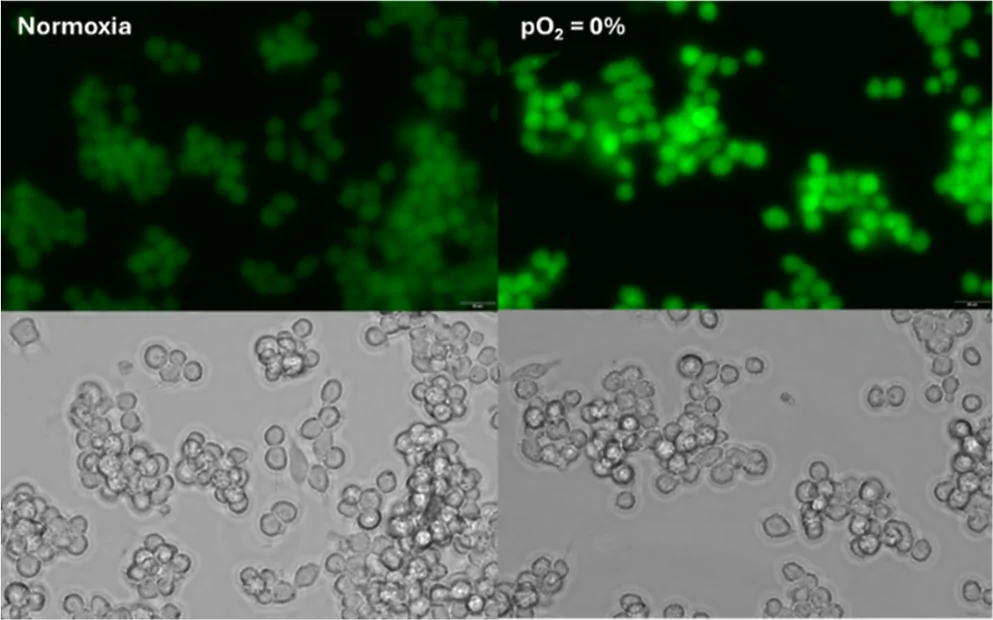
Reduction of probe **1c** by RAW264.7 cells under anaerobic conditions.

### Sulfone Reduction under Hypoxia in HeLa Cells

After determining the feasibility of the probe design using RAW264.7 cells, we extended similar studies to HeLa cells to cross-validate sulfone reduction in different cell lines. A key feature needed in sensing hypoxia is stability of the probe under normoxia (pO_2_: 21%) and reduction of the probe under hypoxia (pO_2_: <0.5%). Therefore, we examined these factors using HeLa cells and one probe set **1** *c* **/2c/3c** as used in RAW264.7 cells^46^.

For cellular fluorescence measurement, we used flow cytometry, as it can record fluorescence in each cell and can be used to quantify the fluorescence changes at different time points. Briefly, we started by incubating probes **1c** and **2c** at 20 μM with HeLa cells at three oxygen concentrations: 21% (normoxia), 0.5% (hypoxia), and anaerobic conditions. Under both hypoxic and anaerobic conditions, a fluorescence intensity increase was observed, indicating sulfone reduction to thioether. Sulfone reduction was found to be dependent on oxygen concentration, with the rate being slower at pO_2_ of 0.5% when compared to anaerobic conditions (Figures 10 and 11). For example, 50% of the cellular population shifted toward increased fluorescence intensity within 28 h at pO_2_ of 0.5%, whereas a similar 50% cellular population shift was observed in just 6 h under anaerobic conditions (Figures 10 and 11). On the other hand, under normoxic conditions, the probe **1c** was found to be stable toward reduction, with only 6% of the cellular population shifted toward increased fluorescence intensity in 6 h and 17% in 28 h (Figure S4). This stability under normoxia is desired. In contrast, the other approaches, including azo-reduction or nitro-reduction, are subject to interference by reduction under mildly hypoxic or normoxic conditions.^83,85^ Specifically, azo-reduction-based probes can be reduced by the gut microbiome, specifically by azoreductases,^83^ and nitro-reduction-based probes can be reduced under mildly hypoxic or normoxic conditions, leading to off-target effects.^85^ For biological applications, it is desired that the probe is activated within minutes to hours. As such, probes such as sulfone **1c** are expected to be very useful because of their highly stable nature under normoxia for up to 6 h and significant activation under hypoxia within 3–6 h. Such results indicate that the reduction-sensitive probe works as designed regardless of the cell line.

**Figure 10:**
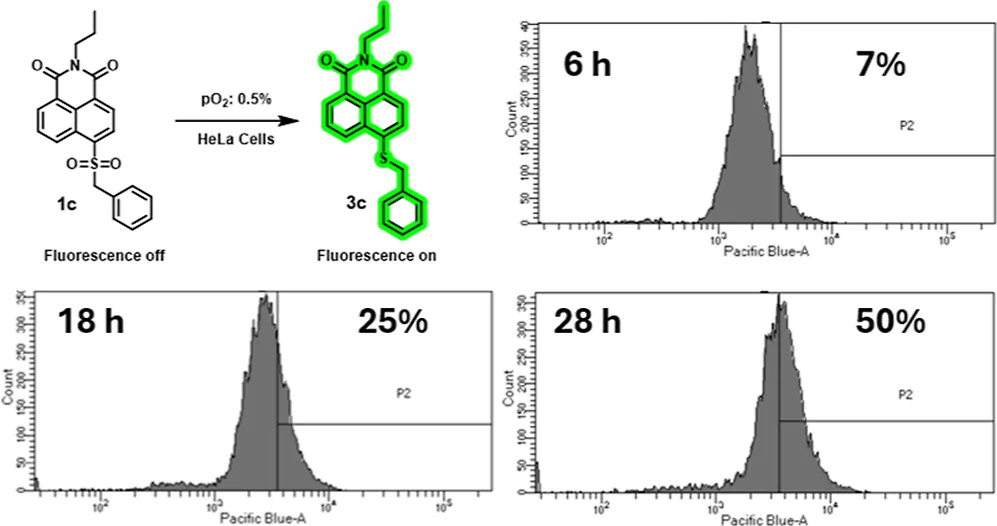
Reduction of probe **1c** by HeLa cells under hypoxia (pO_2_ of 0.5%).

**Figure 11:**
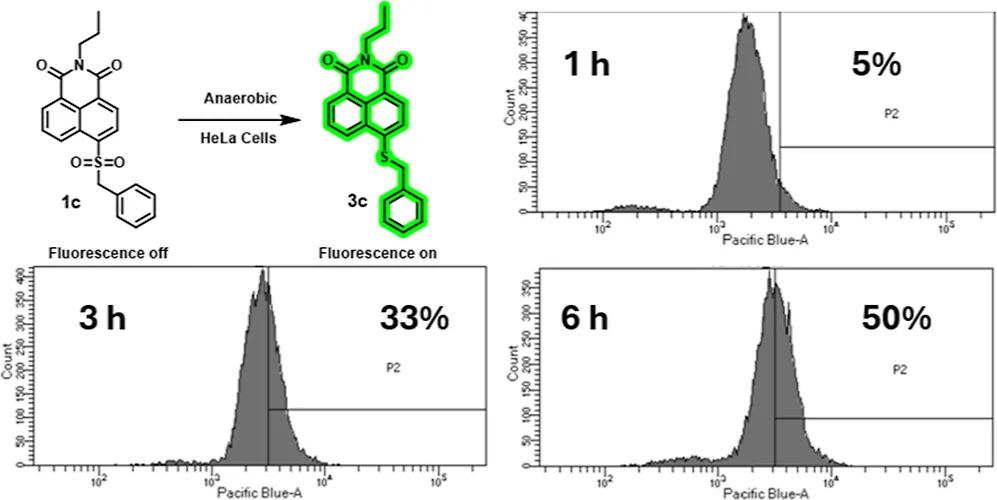
Reduction of probe **1c** by HeLa cells under anaerobic conditions.

### Cellular Hypoxia Sensing Using Sulfoxide Reduction Chemistry

Results of sulfone reduction and probe **1c** activation under hypoxia indicate feasibility of the designed approach. We also conducted additional studies to see if sulfoxide can be reduced to activate the probe to a similar extent in a much shorter time. A similar procedure and detection method/instrument as for sulfone reduction by HeLa cells was used to compare the probe turn-on over time. Briefly, HeLa cells were incubated with probe **2c** at 20 μM for designated times under anaerobic conditions before analysis by flow cytometry. Probe **2c** showed similar reduction kinetics as probe **1c** in the presence of HeLa cells under anaerobic conditions (Figures 11 and 12). Specifically, 20% of the cellular population showed increased fluorescence intensity at 3 h time point and 54% at 6 h time point under hypoxic conditions (Figure 12). On the other hand, under normoxia, only 6% of the fluorescent population showed increase fluorescent intensity at 3 h and 15% at 6 h, indicating a higher propensity for the sulfoxide to be reduced as compared to the sulfone (Figure S5). Sulfoxide probe **2c** showed a 4-fold increase in the cellular population toward higher fluorescence intensity under hypoxia as compared to normoxia (Figures S5 and S12). Overall, both sulfone **1c** and sulfoxide **2c** showed utility for detecting hypoxia and provide new directions in sensing hypoxia or reductive cellular environments.

**Figure 12:**
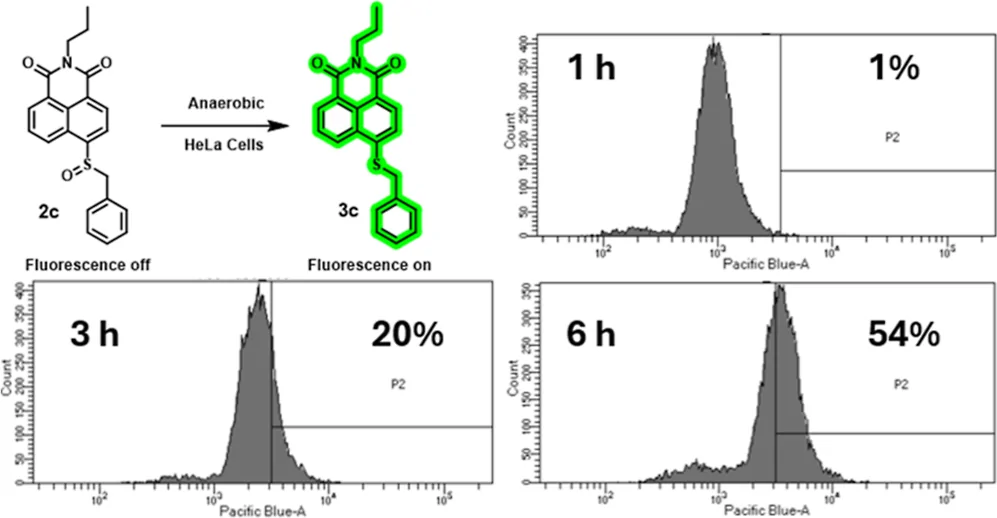
Reduction of probe **2c** by HeLa cells under anaerobic conditions.

### Stability of a Probe with GSH or Other Amino Acids

One critical feature of any probe is to be activated only by the intended mechanism. Along this line, arylsulfoxide- and/or arylsulfone-containing molecules have been reported to react with thiols via a displacement reaction,^101^ and the reactivity depends on the leaving group.^101^ Specifically, methyl sulfone naphthalimide **1d** has been reported to react with GSH,^101^ while benzyl sulfone naphthalimide **1c** does not.^133^ This difference in reactivity has been reported to be dependent on the leaving group at the 4-position of napthalimide.^134^ More mechanistic studies are needed in order to truly understand these differences in reactivity. However, such studies are beyond the scope of this manuscript. Nevertheless, we tested our probes for GSH or amino acid reactivity before cellular studies. First, we tested a positive control by incubating the reported probe **1d** with 1 mM GSH and observed the consumption of probe **1d** and subsequent formation of a new peak at ∼7.4 min within 1 h (Figure S6), which was shown to be consistent with the GSH conjugation product **7** (Figure S7) by LC–MS.^101^ When probes **1** *c* **/2c** were subjected to the same conditions, no substitution reaction was observed, consistent with the literature report of benzyl sulfone being nonreactive toward GSH.^133^ Briefly, we incubated 100 μM of **1c** or **2c** with 2.5 mM GSH in PBS/ACN (1:1) at pH 7.4 and 37 °C (Figure 13A–D). At designated time points, 20 μL was drawn from the reaction mixture and analyzed by HPLC. No stability issues were observed for either sulfone **1c** or sulfoxide **2c** (Figure 13C,D). Similarly, we then studied these probes for reactivity with amino acids using a similar procedure. Briefly, we incubated 10 μM of the probe with 10 μM of amino acid solution in PBS/ACN (1:1) at pH 7.4 and 37 °C for 6 h and found all four probes **1** *c* **/2c** and **1** *a* **/2a** to be stable in the presence of the following four amino acids: l-cysteine, l-lysine, l-histidine, and Boc-serine (Figures 13E,F and S8). These results indicate that the probe does not have reactivity concerns with GSH or amino acids.

**Figure 13:**
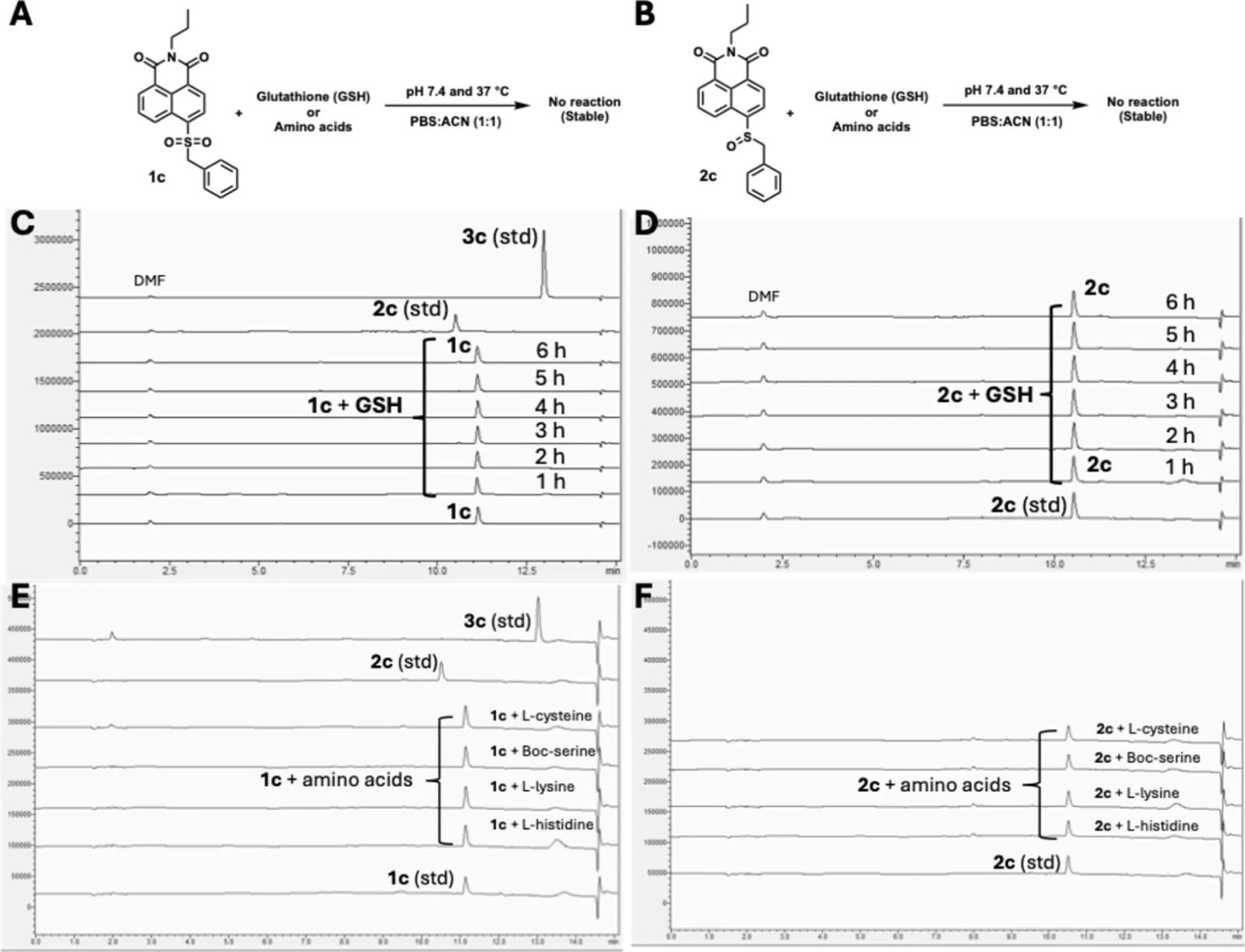
Probe reaction with GSH or amino acids. (A/B). Reaction scheme of probe reaction with GSH or amino acids. (C) HPLC chromatogram showing stability when 100 μM probe **1c** was incubated with 2.5 mM GSH in PBS/ACN (1:1) at pH 7.4 and 37 °C. (D) HPLC chromatogram showing stability when 100 μM probe **2c** was incubated with 2.5 mM GSH in PBS/ACN (1:1) at pH 7.4 and 37 °C. (E) HPLC chromatogram showing stability when 10 μM probe **1c** was incubated with 10 μM amino acids in PBS/ACN (1:1) at pH 7.4 and 37 °C. (F) HPLC chromatogram showing stability when 10 μM probe **2c** was incubated with 10 μM amino acids in PBS/ACN (1:1) at pH 7.4 and 37 °C.

## Conclusion

The association of redox homeostasis with various pathological conditions is given. Tools for studying redox changes are crucial. Herein, we developed a novel reversible approach to detect both reductive and oxidative changes using a redox-responsive probe pair. Furthermore, this approach also has a new activation chemistry for the detection of hypoxia through the selective reduction of a sulfone group to a thioether under severe hypoxia while providing excellent stability under normoxia. Specifically, sulfones **1a** or **1c** were found to turn on selectively by reduction under severe hypoxia in two cell lines: HeLa and RAW264.7 cells. Furthermore, the same probe pair **3a** and **3c** was found to turn off under oxidative stress. However, we should note that we do not have experimental evidence on the specific form of ROS that turned off the fluorescence of **3a,c**. Though all the indications are that the probe fluorescent changes in cell culture experiments were due to hROS production (specifically hypochlorite), a definitive statement can only be made until further rigorous experimental work.

Overall, this approach (1) offers new chemistry to detect the reductive environment and (2) opens a new direction in designing redox-sensitive tools.

## Supplementary Material



## Notes

Synthesis, NMR, experimental protocols, and general information, including materials, instruments, and methods (PDF)
