# Supplementary material for: One Stone Two Birds: Novel Fluorescent Probe Chemistry That Detects Redox Stress in Cell Culture
Source: Anal Chem. 2026 Aug 11;98(33):24327–39. doi: 10.1021/acs.analchem.6c02835 (PMC13523556; doi:10.1021/acs.analchem.6c02835)
Supplement: Supplementary file 1 [file ac6c02835_si_001.pdf]

<u>Supporting Information</u>

One Stone Two Birds: Novel Fluorescent Probe Chemistry that Detects Redox Stress in Cell Culture

Shubham Bansal, Muskan Gori, and Binghe Wang*

Department of Chemistry and Center for Diagnostics and Therapeutics, Georgia State University, Atlanta, Georgia 30303 USA

*Address correspondence to
Dr. Binghe Wang
Regents Professor and Dr. Frank Hannah Chair
Georgia Research Alliance Eminent Scholar
Department of Chemistry
Georgia State University
Atlanta, Georgia 30301
USA
wang@gsu.edu
Phone: 404-413-5544
ORCID: 0000-0002-2200-5270

Table of Contents

1. Experimental Section 2
2. UV-Vis and fluorescence spectra 2
3. Thioether probe reaction with ROS 2
4. Quantum yield (Φ_F) determination 4
5. Probe reaction with GSH or amino acids 4
6. ROS production using LPS or PMA as stimulants 5
   6.1. General protocol for adherent cells including RAW264.7, HeLa, 4T1, H9C2, and HEK293T cells 5
   6.2. General protocol for suspension type cells including RS4;11 cells 5
7. Chemistry 6
8. Supporting figures 11
9. NMR Spectra 15
10. References 27

1. Experimental Section

General information of materials, instruments, and methods: All reagents and solvents were of reagent grade from Sigma-Aldrich (Massachusetts, USA) or Oakwood Products, Inc. (South Carolina, USA). PBS buffer, pH 7.4: 10 mM was purchased from Corning (New York, USA). DCFH-DA (CAS 4091-99-0 and Catalog No. HA-7023) was purchased from Combi-Blocks (California, USA). APF (CAS 359010-70-1 and Catalog No. A36003, 5 mM solution in DMF) was purchased from Invitrogen (Thermo Fisher Scientific, Waltham, MA, USA). Boronate Probe **4** was purchased from Ambeed (Illinois, USA). RAW264.7, HeLa, 4T1, H9C2, RS4;11, and HEK293T cells were purchased from ATTC (Virginia, USA). Fetal bovine serum (FBS), DMEM without sodium pyruvate (Catalog number 10-017-CV) were from Corning (New York, USA) and purchased from ThermoFisher Scientific (Massachusetts, USA). Samples of milligram-quantities were weighed on C-33 microbalance (CAHN instruments Inc., California, USA). For general cell culture HeLa cells were cultured in DMEM without sodium pyruvate supplemented with 10% FBS and 1% PNS. The gas tank having 0.5% oxygen and 5% CO_2 was purchased by Airgas (Pennsylvania, USA). For UV-Vis measurements were performed on Shimadzu UV-1900i UV-Vis spectrophotometer (Kyoto, Japan). HPLC analysis was performed using Shimadzu LC-2050C 3D liquid chromatograph system (Kyoto, Japan). Column: Sunfire C18 3.5 μm, 3.0 × 150 mm. The HPLC mobile phase was made of A: H_2O (0.1% TFA) and B: ACN (0.1% TFA). Flow rate 0.5 mL/min. Gradient methods used: 95% B, 0-10 min; 95% B, 10-12 min; 5% B, 12-12.1 min; 5% B, 12.1-15 min. For incubation under hypoxia, an airtight incubation chamber having water, for humidity control, was used.

2. UV-Vis and fluorescence spectra

Firstly, a 10 mM stock solution of compound was prepared in DMF. Then a 50 μM solution was prepared by adding 15 μL of 10 mM stock to 2.985 mL of PBS:ACN (1:1) and UV-Vis or fluorescence spectra was recorded.

3. Thioether probe reaction with ROS

Firstly, stock solutions were prepared. A 10 mM stock solution of probe was prepared in DMF.

NaOCl: a 100 mM of NaOCl was prepared by adding 55 μL of 1.811 M NaOCl (commercial bottle) to 945 μL of H_2O. After that a 10 mM working solution of NaOCl was prepared by adding 100 μL of 100 mM NaOCl to 900 μL of H_2O.

H_2O_2: A 100 mM H_2O_2 solution was prepared by adding 9 µL of 11 M H_2O_2 (commercial bottle) to a final volume of 991 µL H_2O. The H_2O_2 solution was subsequently diluted by adding 100 µL of 100 mM H_2O_2 to 900 µL water to obtain a 10 mM working solution.

$ONOO^-$: $ONOO^-$ was prepared and quantified to be of 120 mM by following a reported procedure.[1] Then, a 10 mM working solution of $ONOO^-$ was prepared in H_2O by adding 83 µL of 120 mM $ONOO^-$ in 917 µL of H_2O.

To study thioether reactivity towards various ROS:

50 µM probe and 50 µM NaOCl reaction: Firstly, 15 µL of 10 mM probe was added to 2.970 mL of PBS:ACN (1:1) at pH 7.4, followed by the addition of 15 µL of 10 mM NaOCl. After that, the fluorescence spectrum was recorded.

50 µM probe and 50 µM H_2O_2 reaction: Firstly, 15 µL of 10 mM probe was added to 2.970 mL of PBS:MeOH (1:1) at pH 7.4, followed by the addition of 15 µL of 10 mM H_2O_2 solution. After that, the fluorescence spectrum was recorded.

50 µM probe and 50 µM $ONOO^-$ reaction: Firstly, 15 µL of 10 mM probe was added to 2.970 mL of PBS:ACN (1:1) at pH 7.4, followed by the addition of 15 µL of 10 mM $ONOO^-$. After that, the fluorescence spectrum was recorded.

50 µM probe and hydroxyl radical reaction: Firstly, 15 µL of 10 mM probe was added to 2.655 mL of PBS:ACN (1:1) at pH 7.4, followed by the addition of 30 µL of 10 mM ferrous ammonium sulfate and 300 µL of 10 mM H_2O_2. Then the fluorescence spectrum was recorded.

For the reaction of 10 µM APF probe and hydroxyl radical reaction: Firstly, 6 µL of 5 mM probe was added to 2.664 mL of PBS:ACN (1:1) at pH 7.4, followed by the addition of 30 µL of 10 mM ferrous ammonium sulfate and 300 µL of 10 mM H_2O_2. Then the fluorescence spectrum was recorded using 325 nm as excitation wavelength.

For the reaction of boronate probe **4** reaction with $ONOO^-$: $ONOO^-$ was prepared and quantified to be of 101 mM by following a reported procedure.[1] Then, 15 µL of 10 mM probe was added to 2.983 mL of PBS:ACN (1:1) at pH 7.4, followed by the addition of 1.48 µL of 82 mM $ONOO^-$. After that, the fluorescence spectrum was recorded using 492 nm as excitation wavelength.

4. Quantum yield (Φ_F) determination

The fluorescence quantum yields of the compounds were determined using quinine sulfate as a reference standard (Φ_F = 0.55 in 0.5 M H_2SO_4, η = 1.346, Ir = 18131 at 5/5 nm excitation/emission bandwidths, Ar = 0.051). Quantum yields were calculated using the equation shown below (equation S1). Compounds were prepared in PBS at concentrations ranging from 10 to 20 µM, and their absorbance values were measured at their maximum excitation wavelength.[2, 3]

$$\Phi_x = \Phi_r . \frac{I_x A_r \lambda_r \eta_x^2}{I_r A_x \lambda_x \eta_r^2} \text{............ (S1)}$$

In this equation, the subscripts *r* and *x* represent the reference and test compounds, respectively. Φ denotes the quantum yield, I correspond to the integrated fluorescence emission area, A is the absorbance measured at the excitation wavelength, λ represents the excitation wavelength, and η is the refractive index of the solvent.

Compound	λex (nm)	I_x	A_x	η (medium)	Φ_F
3a	382	8290	0.059	1.3349 (PBS)	0.21
3b	390	3053	0.048	1.3349 (PBS)	0.09
3c	385	3358	0.050	1.3349 (PBS)	0.10

Table S1: Quantum yield determination

5. Probe reaction with GSH or amino acids

For studying reactivity with GSH, firstly, a 10-mM stock solution of compound was prepared in DMF and 50-mM GSH solution was prepared in H_2O. Then, 10 µL was taken from the 10 mM stock solution of compound and added to 940 µL of PBS:ACN (1:1). This was followed by the addition of 50 µL of 50 mM GSH solution. The reaction mixture had a final concentration of 100 µM of probe and 2.5 mM of GSH in PBS:ACN (1:1) containing 1% DMF at 37 ˚C. Probe **1d** was tested at the reported conditions of 100 µM of probe and 1 mM of GSH.[4]

For studying reactivity with amino acids, a 10-mM stock solution of compound was prepared in DMF, and 1 mM amino acid solution was prepared in H_2O. Then, 1 µL was taken from the 10 mM stock solution of compound was added to 989 µL of PBS:ACN (1:1). This was followed by the addition of 10 µL of 1 mM amino acid solution. The reaction mixture had a final concentration of 10 µM of probe and 10 µM of amino acid in PBS:ACN (1:1) containing 1% DMF at 37 ˚C.

Then after 6 h of oincubation, 20 μL aliquot was drawn from the reaction mixture and injected into HPLC.

6. ROS production using LPS or PMA as stimulants

6.1. General protocol for adherent cells including RAW264.7, HeLa, 4T1, H9C2, and HEK293T cells

First, stock solutions were prepared as 10-mM stock solution of DCFH-DA in DMF, 10-mM PMA in DMF, and 2-μg/μL of LPS in H_2O. Then, working solutions were prepared as 10 μM DCFH-DA only by adding 5 μL of 10 mM DCFH-DA to 5 mL DMEM Fluorobrite medium; 10 μM DCFH-DA and 2 μg/mL LPS by adding 5 μL of 10 mM DCFH-DA and 5 μL 2 μg/μL LPS to 5 mL DMEM Fluorobrite medium; 10 μM DCFH-DA and 2 μM PMA by adding 5 μL of 10 mM DCFH-DA and 1 μL of 10 mM PMA to 5 mL DMEM Fluorobrite medium. Subsequently a 96-well plate having all sides black and clear bottom suitable for fluorescence measurement was prepared to have 80% cell confluency. Media was removed from each well of the 96-well plate and washed with 100 μL of cold PBS. After that, 100 μL of the designated working solution was added to the designated wells and the plate was then incubated at 37 °C before fluorescent measurement. At the designated time, the media was removed, and the cells were washed twice with 100 μL of cold PBS and then 100 μL of fresh DMEM Fluorobrite medium was added to the cells and the fluorescence was measured from the bottom side of the 96-well plate using a plate reader (λ_{ex} 495 nm and λ_{em} 530 nm). The final concentrations were 10 μM DCFH-DA only; 10 μM DCFH-DA and 2 μg/mL LPS; and 10 μM DCFH-DA and 2 μM PMA.

6.2. General protocol for suspension type cells including RS4;11 cells

First, stock solutions were prepared as 10-mM stock solution of DCFH-DA in DMF, 10 mM PMA in DMF, and 2 μg/μL of LPS in H_2O. Then, working solutions were prepared at 2-fold of final concentrations as 20 μM DCFH-DA only by adding 10 μL of 10 mM DCFH-DA to 5 mL DMEM Fluorobrite medium; 20 μM DCFH-DA and 4 μg/mL LPS by adding 10 μL of 10 mM DCFH-DA and 10 μL of 2 μg/μL LPS to 5 mL DMEM Fluorobrite medium; 20 μM DCFH-DA and 4 μM PMA by adding 10 μL of 10 mM DCFH-DA and 2 μL of 10 mM PMA to 5 mL DMEM Fluorobrite medium. Subsequently a pellet of RS4;11 cells were suspended in DMEM Fluorobrite medium and then 100 μL of the cell containing media was added to the designated wells. After that, 100 μL of the designated working solution was added to the designated wells and the plate

was then incubated at 37 °C before fluorescent measurement. At the designated time, the fluorescence was measured from the top side of the 96-well plate using a plate reader (λ_{ex} 495 nm and λ_{em} 530 nm). The final concentrations were 10 μM DCFH-DA; 10 μM DCFH-DA and 2 μg/mL LPS; and 10 μM DCFH-DA and 2 μM PMA.

7. Chemistry

2-((1,3-Dioxo-1H,3H-benzo[de]isochromen-6-yl)thio)-N-methylacetamide (6a): To a solution of 2-mercapto-N-methylacetamide (91 mg, 1.2 eq., 0.86 mmol) and potassium carbonate (3 eq) in DMF, compound **4** (200 mg, 1 eq, 0.72 mmol) was added. The reaction mixture was heated at reflux, and the reaction was monitored using TLC. After completion of reaction, the mixture was poured into ice cold water, and the compound was precipitated. The precipitates were collected using filtration. The crude product was purified using a silica gel column and methanol/DCM (dichloromethane) as eluent. Yellow colored product, isolated yield: 180 mg, 82%. ^{1}H NMR (DMSO) δ 8.61 (dd, *J* = 28.9, 7.8 Hz, 2H), 8.41 (d, *J* = 7.9 Hz, 1H), 8.30 (s, 1H), 8.00 – 7.89 (m, 1H), 7.78 (d, *J* = 8.0 Hz, 1H), 4.05 (s, 2H), 2.63 (d, *J* = 4.6 Hz, 3H).^{13}C NMR (DMSO) δ 167.4, 161.0, 160.8, 146.3, 133.3, 132.5, 131.2, 130.2, 129.1, 128.0, 124.1, 120.0, 115.8, 35.7, 26.4. HRMS calculated for $C_{15}H_{11}NO_4S$ $(M+H)^+$: 302.0487, found 302.0486.

6-(Phenylthio)-1H,3H-benzo[de]isochromene-1,3-dione (6b): To a solution of thiophenol (230 mg, 2.1 mmol, 1.2 eq) and potassium carbonate (751 mg, 5.4 mmol, 3 eq) in DMF, compound **4** (500 mg, 1.8 mmol, 1 eq) was added. The reaction mixture was heated under reflux, and the reaction was monitored using TLC. After completion of reaction, the mixture was poured into ice cold water, and the compound was precipitated. The precipitates were collected using filtration. The crude product was purified using a silica gel column and ethyl acetate/hexane as eluent. Yellow colored product, isolated yield: 420 mg, 75%. ^{1}H NMR ($CDCl_3$) δ 8.76 (dd, *J* = 8.5, 1.0 Hz, 1H), 8.70 (dd, *J* = 7.3, 1.0 Hz, 1H), 8.37 (d, *J* = 8.0 Hz, 1H), 7.88 (dd, *J* = 8.4, 7.4 Hz, 1H), 7.58 (tdd, *J* = 9.6, 6.4, 3.2 Hz, 5H), 7.21 (d, *J* = 8.0 Hz, 1H). ^{13}C NMR ($CDCl_3$) δ 160.6, 160.3, 148.5, 135.1, 133.8, 132.9, 131.4, 130.5, 130.3, 130.1, 129.3, 129.0, 127.2, 124.8, 119.3, 115.3. HRMS calculated for $C_{18}H_{10}O_3S$ $(M+H)^+$: 307.0429, found 307.0417

6-(Benzylthio)-1H,3H-benzo[de]isochromene-1,3-dione (6c): The compound was synthesized by following reported procedure.[5] Yellow solid; Isolated yield: 204 mg, 70% yield. ^{1}H NMR ($CDCl_3$) δ 8.68 – 8.65 (m, 2H), 8.49 (d, *J* = 7.9 Hz, 1H), 7.83 (d, *J* = 7.5 Hz, 1H), 7.64 (d, *J* = 7.9

Hz, 1H), 7.46 (d, J = 7.0 Hz, 2H), 7.36 (dd, J = 10.8, 7.2 Hz, 3H), 4.44 (s, 2H). HRMS calculated for $C_{19}H_{12}O_3S$ $(M+H)^+$: 321.0599 found 321.0585.

6-(Methylthio)-1H,3H-benzo[de]isochromene-1,3-dione (6d): The compound was synthesized by following reported procedure.[4] Yellow solid; Isolated yield: 190 mg, 85 % yield ^{1}H NMR ($CDCl_3$) ^{1}H NMR ($CDCl_3$) δ 8.68 (d, J = 7.3 Hz, 1H), 8.63 (d, J = 8.5 Hz, 1H), 8.54 (d, J = 7.9 Hz, 1H), 7.87 – 7.81 (m, 1H), 7.53 (d, J = 8.0 Hz, 1H), 2.76 (s, 3H). HRMS calculated for $C_{13}H_9O_3S$ $(M+H)^+$: 245.0272 and found: 245.0263.

2-((1,3-Dioxo-2-propyl-2,3-dihydro-1H-benzo[de]isoquinolin-6-yl)thio)-N-methylacetamide (3a): To a round bottom flask, compound **6a** (120 mg, 0.39 mmol, 1 eq) was dissolved in ethanol and then n-propyl amine (1.2 eq) was added. Then, the reaction mixture was refluxed for 2 h. After that the reaction mixture was allowed to cool down, leading to the formation of precipitates. The precipitates were then collected by filtration, and the crude product was then washed with distilled water. The crude product was then purified using a silica gel column and methanol/DCM as eluent. Yellow solid; isolated yield: 115 mg, 86%. ^{1}H NMR (DMSO) δ 8.56 (dd, J = 12.5, 7.9 Hz, 2H), 8.38 (d, J = 7.9 Hz, 1H), 8.27 (s, 1H), 7.93 – 7.89 (m, 1H), 7.76 (d, J = 8.0 Hz, 1H), 4.06 – 3.97 (m, 4H), 2.63 (d, J = 4.6 Hz, 3H), 1.65 (dd, J = 14.8, 7.4 Hz, 2H), 0.92 (t, J = 7.4 Hz, 3H). ^{13}C NMR (DMSO) δ 167.6, 163.7, 163.6, 131.6, 130.9, 130.1, 129.0, 127.9, 127.8, 123.8, 123.1, 119.2, 41.6, 35.5, 26.4, 21.3, 11.8. HRMS calculated for $C_{18}H_{18}N_2O_3S$ $(M+H)^+$: 343.1116, found 343.1130.

6-(Phenylthio)-2-propyl-1H-benzo[de]isoquinoline-1,3(2H)-dione (3b): To the solution of n-propylamine (1.2 eq) in 5 ml of ethanol compound **6b** (150 mg, 0.49 mmol, 1 eq) was added and the resulting reaction mixture was refluxed. The reaction was monitored using TLC. After completion of reaction, the mixture was then poured into ice cold water leading to the formation of precipitates. The precipitates were then collected by filtration, and the crude product was then washed with distilled water. The crude product was then purified using a silica gel column and ethyl acetate/hexane as eluent. Yellow colored product, isolated yield: 145 mg, 85%. ^{1}H NMR (CD_3OD) δ 8.60 (dd, J = 34.1, 7.8 Hz, 2H), 8.27 (d, J = 7.8 Hz, 1H), 7.82 (t, J = 7.9 Hz, 1H), 7.63 – 7.49 (m, 5H), 7.26 (d, J = 7.8 Hz, 1H), 4.08 (dd, J = 8.3, 6.8 Hz, 2H), 1.79 – 1.69 (m, 2H), 1.00 (t, J = 7.4 Hz, 3H). ^{13}C NMR ($CDCl_3$) δ 164.0, 163.9, 144.9, 134.0, 131.6, 131.1, 130.8, 130.2,

130.0, 129.5, 129.2, 128.5, 126.9, 126.1, 123.2, 120.3, 41.9, 21.4, 11.5. HRMS calculated for $C_{21}H_{17}NO_2S$ $(M+H)^+$: 348.1058, found 348.1075

6-(Benzylthio)-2-propyl-1H-benzo[de]isoquinoline-1,3(2H)-dione (3c): To a round bottom flask, compound **6b** (100 mg, 2.58 mmol, 1 eq) was dissolved in ethanol and then n-propyl amine (1.2 eq) was added. The reaction mixture was then heated under reflux for 2 h. After that, the reaction mixture was allowed to cool down, leading to the formation of precipitates. The precipitates were then collected by filtration, and the crude product was then washed with distilled water. The crude product was then purified using a silica gel column and ethyl acetate/hexane as eluent. Yellow solid; 90%, isolated yield: 103 mg. ^{1}H NMR ($CDCl_3$) δ 8.62 (dd, *J* = 21.1, 7.9 Hz, 2H), 8.46 (d, *J* = 7.8 Hz, 1H), 7.79 – 7.74 (m, 1H), 7.61 (d, *J* = 7.9 Hz, 1H), 7.44 – 7.31 (m, 5H), 4.39 (s, 2H), 4.18 – 4.13 (m, 2H), 1.82 – 1.74 (m, 2H), 1.03 (t, *J* = 7.4 Hz, 3H). ^{13}C NMR ($CDCl_3$) δ 164.0, 164.0, 144.3, 135.3, 131.5, 130.7, 130.1, 129.7, 128.9, 128.8, 128.3, 127.8, 126.7, 124.1, 123.1, 119.7, 77.0, 76.7, 41.9, 37.5, 21.4, 11.5. HRMS calculated for $C_{22}H_{19}NO_2S$ $(M+H)^+$: 362.1215, found: 362.1205.

2-Butyl-6-(methylthio)-1H-benzo[de]isoquinoline-1,3(2H)-dione (3d): The compound was synthesized by following reported procedure.[6] Yellow solid; Isolated yield: 110 mg, 90% yield. ^{1}HNMR ($CDCl_3$) δ 8.66, 8.66, 8.64, 8.64, 8.55, 8.55, 8.53, 8.51, 7.79, 7.78, 7.77, 7.75, 7.49, 7.47, 4.22, 4.20, 4.18, 2.72, 1.74, 1.48, 1.46, 1.02, 1.00, 0.98. HRMS calculated for $C_{17}H_{18}NO_2S$ $(M+H)^+$: 300.1058 found: 300.1044.

For synthesis of sulfone and sulfoxide, corresponding thioether was added to a round bottom flask containing DCM (dichloromethane). To the flask, m-chloroperbenzoic acid (m-CPBA) (0.9 eq for sulfoxide and 3 eq for sulfone) was added. The reaction was stirred at room temperature and monitored using TLC. After completion of reaction, sodium bicarbonate was used to quench it and product was extracted using 4 × 40 mL DCM. The organic layers were combined and dried over anhydrous sodium sulfate. After that, the DCM layer was filtered, and filtrate was concentrated under reduced pressure using a rotary evaporator. The crude product was then purified by chromatography using a silica gel column.

2-**((1,3-Dioxo-2-propyl-2,3-dihydro-1H-benzo[de]isoquinolin-6-yl)sulfonyl)-N-methylacetamide (1a):** ^{1}H NMR ($CDCl_3$) δ 9.09 (d, *J* = 8.7 Hz, 2H), 8.83 – 8.68 (m, 4H), 8.51 (dd, *J* = 7.7, 2.6 Hz, 2H), 8.01 (dd, *J* = 11.0, 5.0 Hz, 2H), 6.56 (s, 3H), 4.27 – 4.08 (m, 8H), 2.86

(d, J = 4.8 Hz, 6H), 1.79 (dd, J = 15.0, 7.5 Hz, 13H), 1.05 (t, J = 7.4 Hz, 6H).^{13}C NMR ($CDCl_3$) δ 163.2, 160.2, 138.2, 132.1, 130.8, 129.6, 129.3, 128.9, 128.3, 127.6, 123.7, 62.1, 42.3, 27.0, 21.3, 11.5**.** HRMS calculated for $C_{18}H_{18}N_2O_5S$ $(M+H)^+$: 375.1015, found: 375.0995

2**-(Phenylsulfonyl)-2-propyl-1H-benzo[de]isoquinoline-1,3(2H)-dione (1b):** ^{1}H NMR ($CDCl_3$) δ 9.06 (d, J = 8.7 Hz, 1H), 8.75 – 8.61 (m, 3H), 7.99 (d, J = 7.3 Hz, 2H), 7.86 (dd, J = 8.6, 7.5 Hz, 1H), 7.56 (dd, J = 21.3, 7.5 Hz, 3H), 4.17 – 4.07 (m, 2H), 1.74 (d, J = 7.5 Hz, 3H), 1.00 (t, J = 7.4 Hz, 3H). ^{13}C NMR ($CDCl_3$) δ 163.4, 162.9, 141.5, 140.7, 133.8, 131.8, 130.4, 129.6, 129.5, 129.5, 128.9, 127.6, 127.5, 127.22, 123.3, 42.2, 21.3, 11.4. HRMS calculated for $C_{21}H_{18}NO_4S$ $(M+H)^+$: 380.0957, found 380.0965.

6-(Benzylsulfonyl)-2-propyl-1H-benzo[de]isoquinoline-1,3(2H)-dione (1c): Isolated yield: 86 mg, 80%; ^{1}H NMR ($CDCl_3$) δ 8.99 (dd, J = 8.7, 1.0 Hz, 1H), 8.71 (dd, J = 7.3, 1.0 Hz, 1H), 8.58 (d, J = 7.7 Hz, 1H), 8.23 (d, J = 7.7 Hz, 1H), 7.86 (dd, J = 8.7, 7.3 Hz, 1H), 7.27 (s, 4H), 7.18 (t, J = 7.6 Hz, 2H), 6.96 (d, J = 7.3 Hz, 2H), 4.56 (s, 2H), 4.22 – 4.12 (m, 2H), 1.80 (d, J = 7.6 Hz, 2H), 1.05 (t, J = 7.4 Hz, 3H). ^{13}C NMR ($CDCl_3$) δ 163.4, 162.8, 138.4, 131.8, 131.4, 130.6, 130.3, 129.2, 129.2, 129.0, 128.7, 128.5, 128.1, 127.5, 127.1, 123.2, 63.3, 42.3, 21.3, 11.5. HRMS calculated for $C_{22}H_{19}NO_4S$ $(M+H)^+$: 394.1113, found: 394.1100.

2-Butyl-6-(methylsulfonyl)-1H-benzo[de]isoquinoline-1,3(2H)-dione (1d): The compound was synthesized by following reported procedure.[4] White solid; Isolated yield: 86% yield, 46 mg. ^{1}H NMR ($CDCl_3$) δ 9.14 (dd, J = 8.7, 0.9 Hz, 1H), 8.78 – 8.74 (m, 2H), 8.59 (d, J = 7.7 Hz, 1H), 8.01 (dd, J = 8.6, 7.4 Hz, 1H), 4.24 – 4.20 (m, 2H), 3.29 (s, 3H), 1.73 (dd, J = 7.3, 2.3 Hz, 2H), 1.48 (d, J = 7.7 Hz, 2H), 1.02 (d, J = 7.3 Hz, 3H). HRMS calculated for $C_{17}H_{18}NO_4S$ $(M+H)^+$: 332.0957 found 332.0939.

2**-((1,3-Dioxo-2-propyl-2,3-dihydro-1H-benzo[de]isoquinolin-6-yl)sulfinyl)-N-methylacetamide (2a):** ^{1}H NMR ($CDCl_3$) δ 8.79 (d, J = 7.7 Hz, 1H), 8.72 (d, J = 7.2 Hz, 1H), 8.35 (d, J = 7.7 Hz, 1H), 8.26 (d, J = 8.0 Hz, 1H), 7.95 – 7.88 (m, 1H), 4.23 – 4.13 (m, 2H), 3.90 (d, J = 14.0 Hz, 1H), 3.59 (d, J = 13.9 Hz, 1H), 2.86 (d, J = 4.9 Hz, 3H), 1.79 (dt, J = 14.9, 7.5 Hz, 2H), 1.05 (t, J = 7.4 Hz, 3H).^{13}C NMR ($CDCl_3$) δ 163.3, 163.2, 144.4, 131.8, 130.3, 128.6, 128.2, 127.0, 126.8, 125.8, 123.6, 58.7, 42.2, 26.6, 26.6, 21.3, 11.5. HRMS calculated for $C_{18}H_{18}N_2O_5S$ $(M+H)^+$: 359.1066, found: 359.1082.

6-(Phenylsulfinyl)-2-propyl-1H-benzo[de]isoquinoline-1,3(2H)-dione (2b): ^{1}H NMR ($CDCl_3$) δ 9.09 (d, *J* = 8.7 Hz, 1H), 8.76 – 8.61 (m, 3H), 8.01 (d, *J* = 8.3 Hz, 2H), 7.88 (s, 1H), 7.56 (d, *J* = 7.8 Hz, 3H), 4.15 (t, *J* = 7.5 Hz, 2H), 1.76 (d, *J* = 7.4 Hz, 2H), 1.02 (td, *J* = 7.4, 3.3 Hz, 4H). ^{13}C NMR ($CDCl_3$) δ 163.5, 163.2, 147.7, 144.1, 131.7, 131.6, 130.6, 129.7, 128.5, 128.2, 128.0, 127.6, 125.3, 125.1, 123.9, 123.5, 77.3, 77.0, 76.7, 42.1, 21.3, 11.4., HRMS calculated for $C_{21}H_{17}NO_3S$ $(M+H)^+$: 364.1007, found 364.1019

6**-(Benzylsulfinyl)-2-propyl-1H-benzo[de]isoquinoline-1,3(2H)-dione (2c):** Isolated yield: 65 mg, 60%. ^{1}H NMR ($CDCl_3$) δ 8.65 (dd, *J* = 11.1, 7.5 Hz, 2H), 8.19 – 8.13 (m, 1H), 8.05 (d, *J* = 7.7 Hz, 1H), 7.75 (dd, *J* = 8.3, 7.5 Hz, 1H), 7.17 (t, *J* = 7.5 Hz, 2H), 6.85 (d, *J* = 7.2 Hz, 2H), 4.33 – 4.21 (m, 2H), 4.18 (dd, *J* = 8.5, 6.8 Hz, 2H), 1.81 (d, *J* = 7.6 Hz, 2H), 1.06 (t, *J* = 7.4 Hz, 3H). ^{13}C NMR ($CDCl_3$) δ 163.5, 163.4, 131.4, 130.3, 130.2, 130.2, 129.8, 128.7, 128.5, 128.1, 127.9, 127.8, 127.3, 124.8, 124.3, 123.4, 62.5, 42.2, 21.3, 11.5. HRMS calculated for $C_{22}H_{19}NO_3S$ $(M+H)^+$: 378.1164, found: 378.1162.

8. Supporting figures

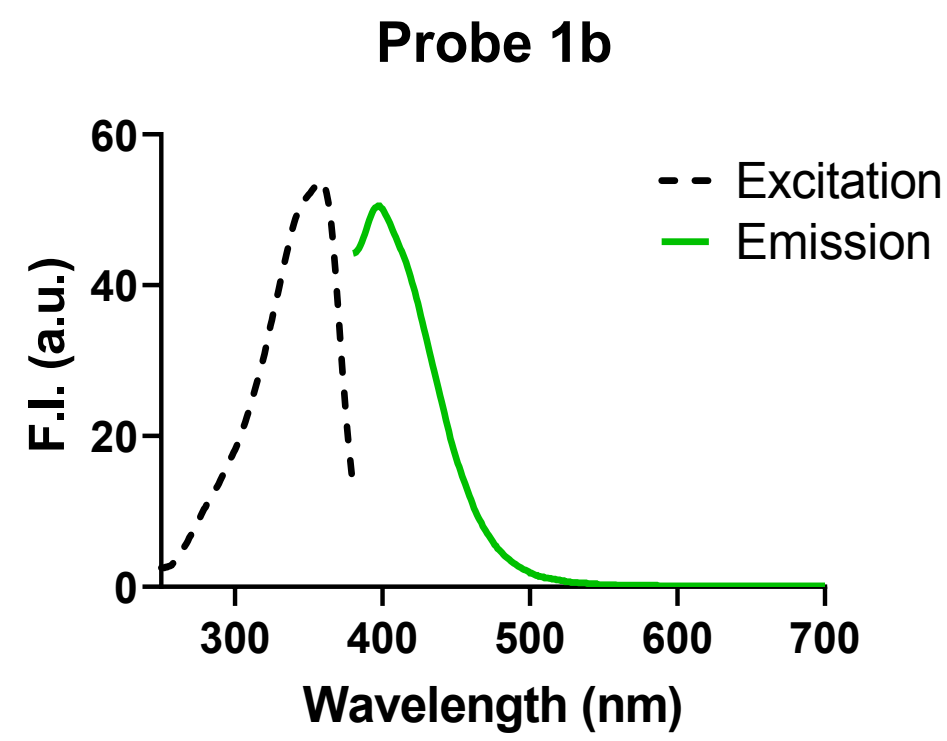

Figure S1. Excitation and emission spectra of probe **1b**.

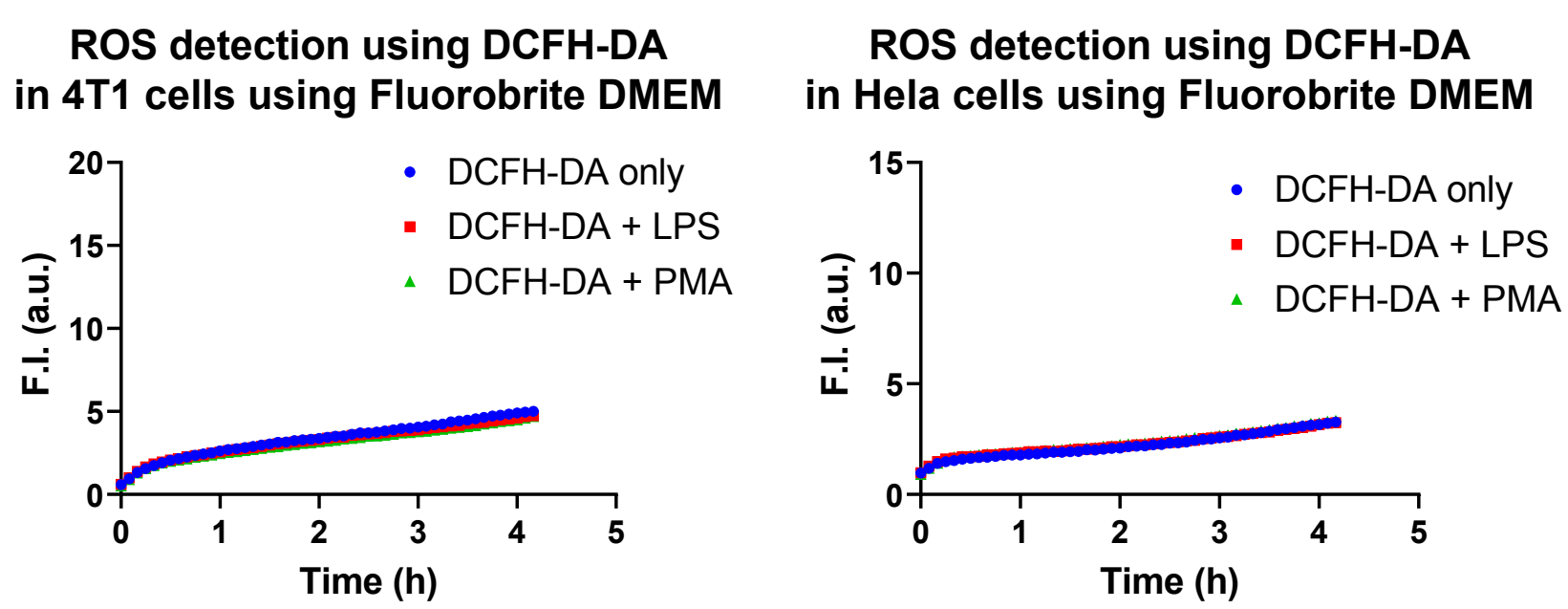

Figure S2. ROS production in HeLa or 4T1 upon stimulation with LPS or PMA.

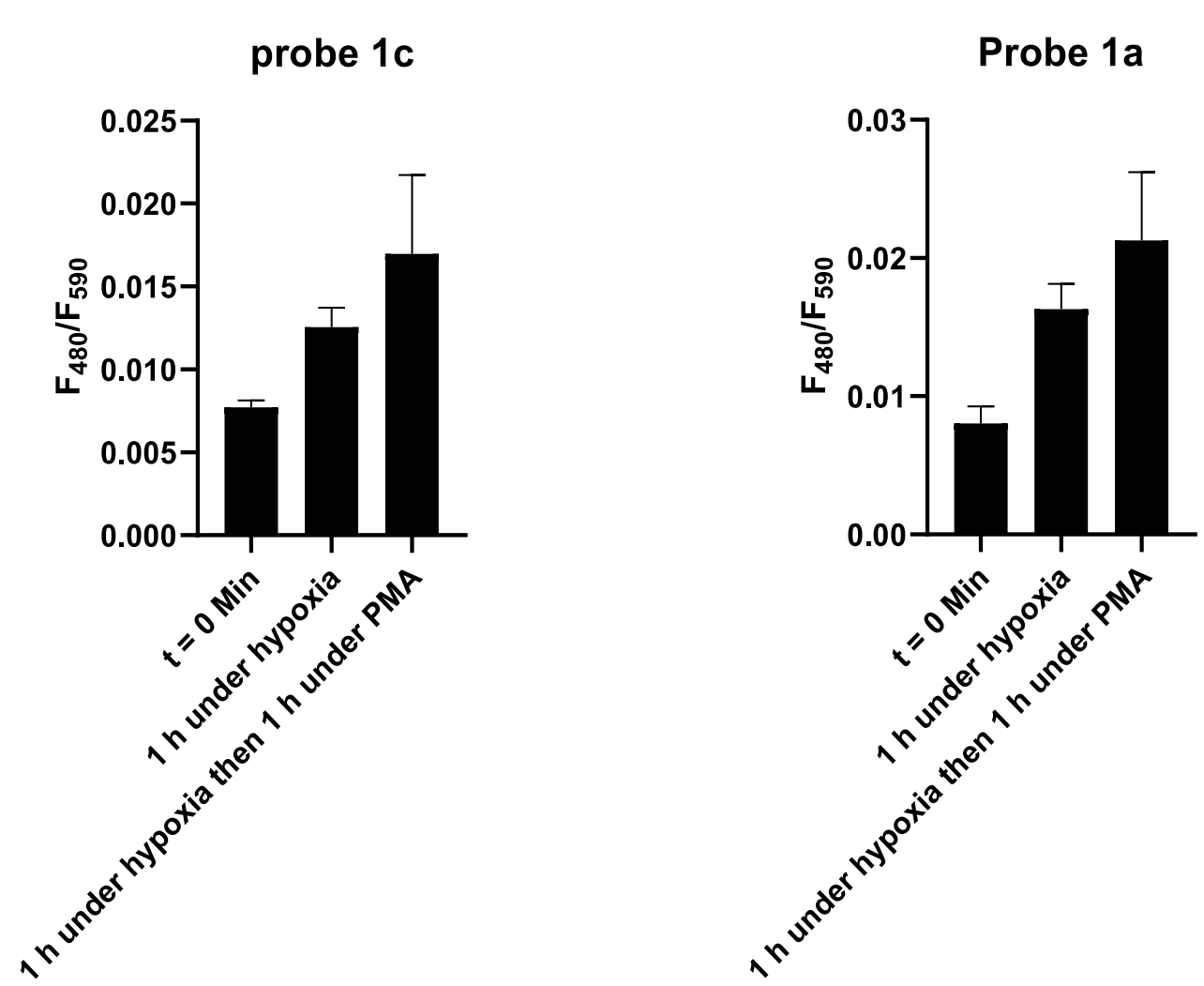

Figure S3. Sensing redox state in RAW264.7 cells. Rhodamine B was used as an internal standard and y-axis represent ratio of emission intensity at 480 nm and 590 nm.

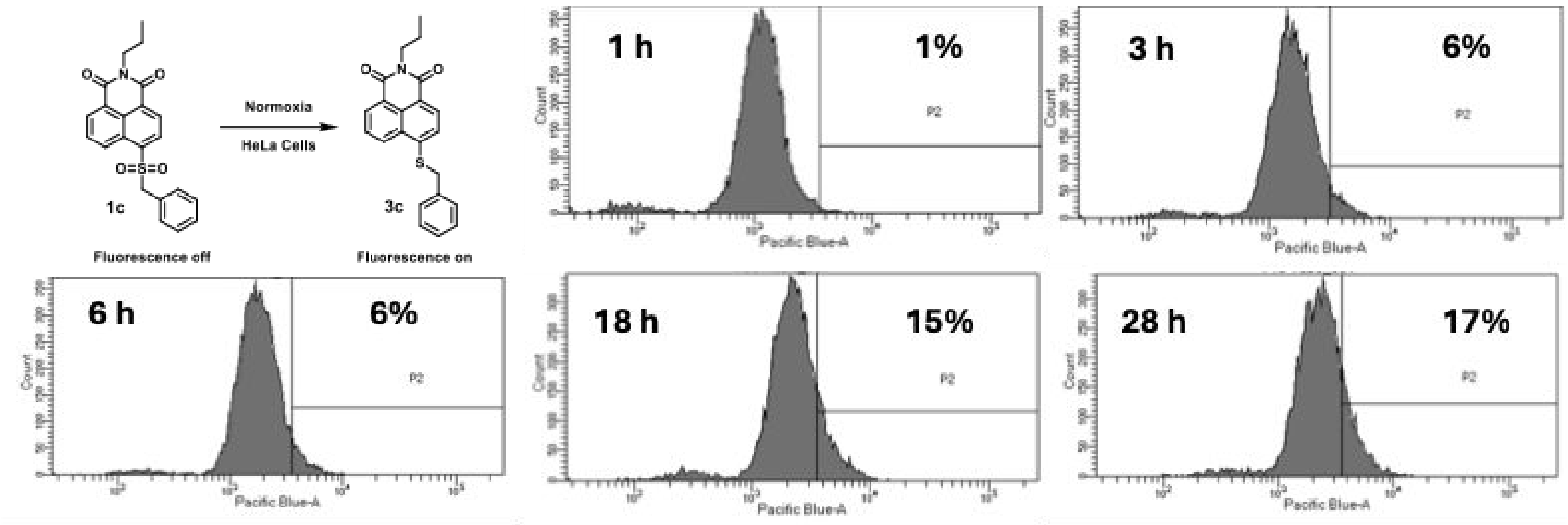

Figure S4. Reduction of sulfone **1c** in HeLa cells under in normoxia.

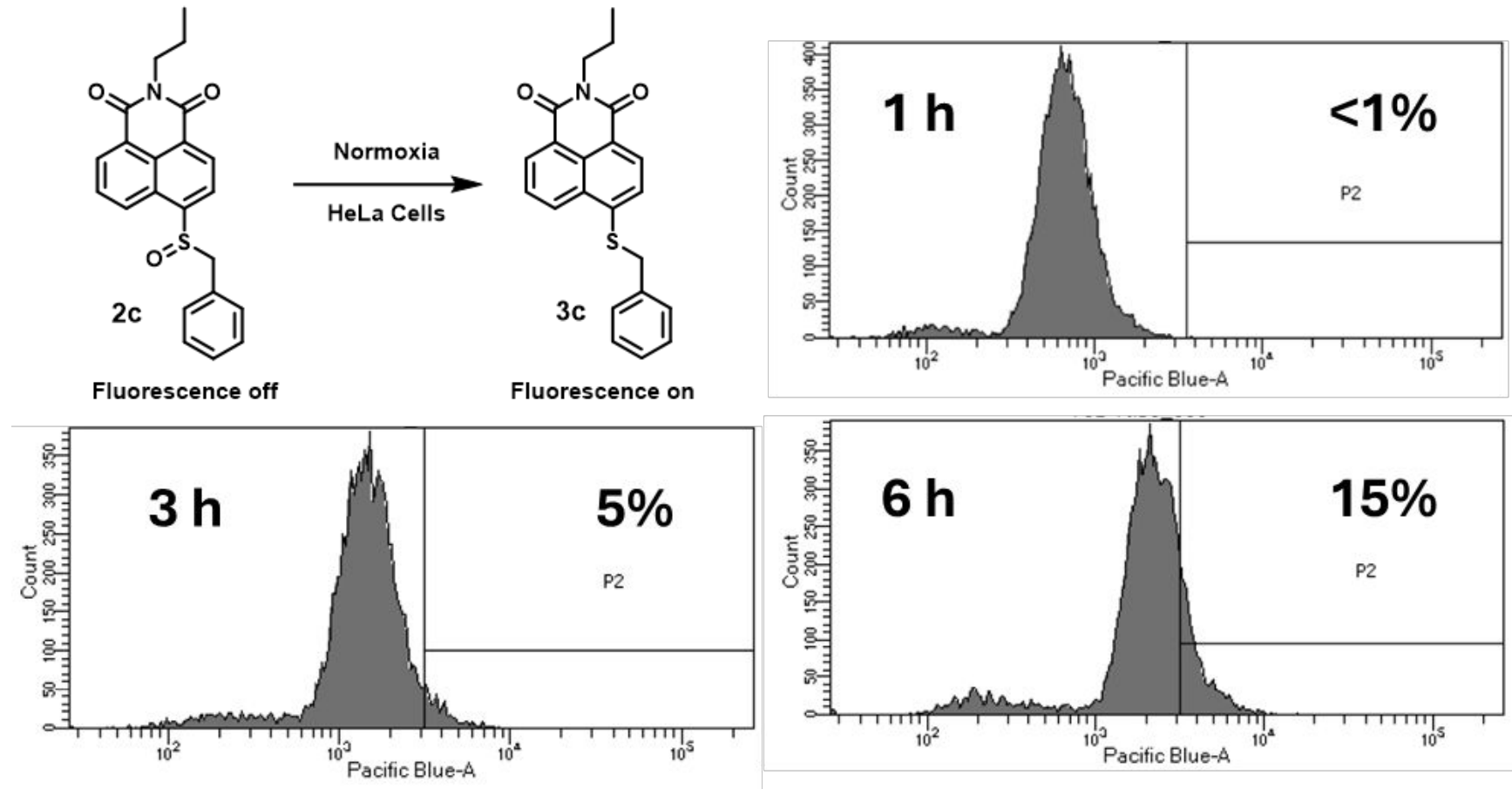

Figure S5. Reduction of sulfoxide **2c** in HeLa cells under in normoxia.

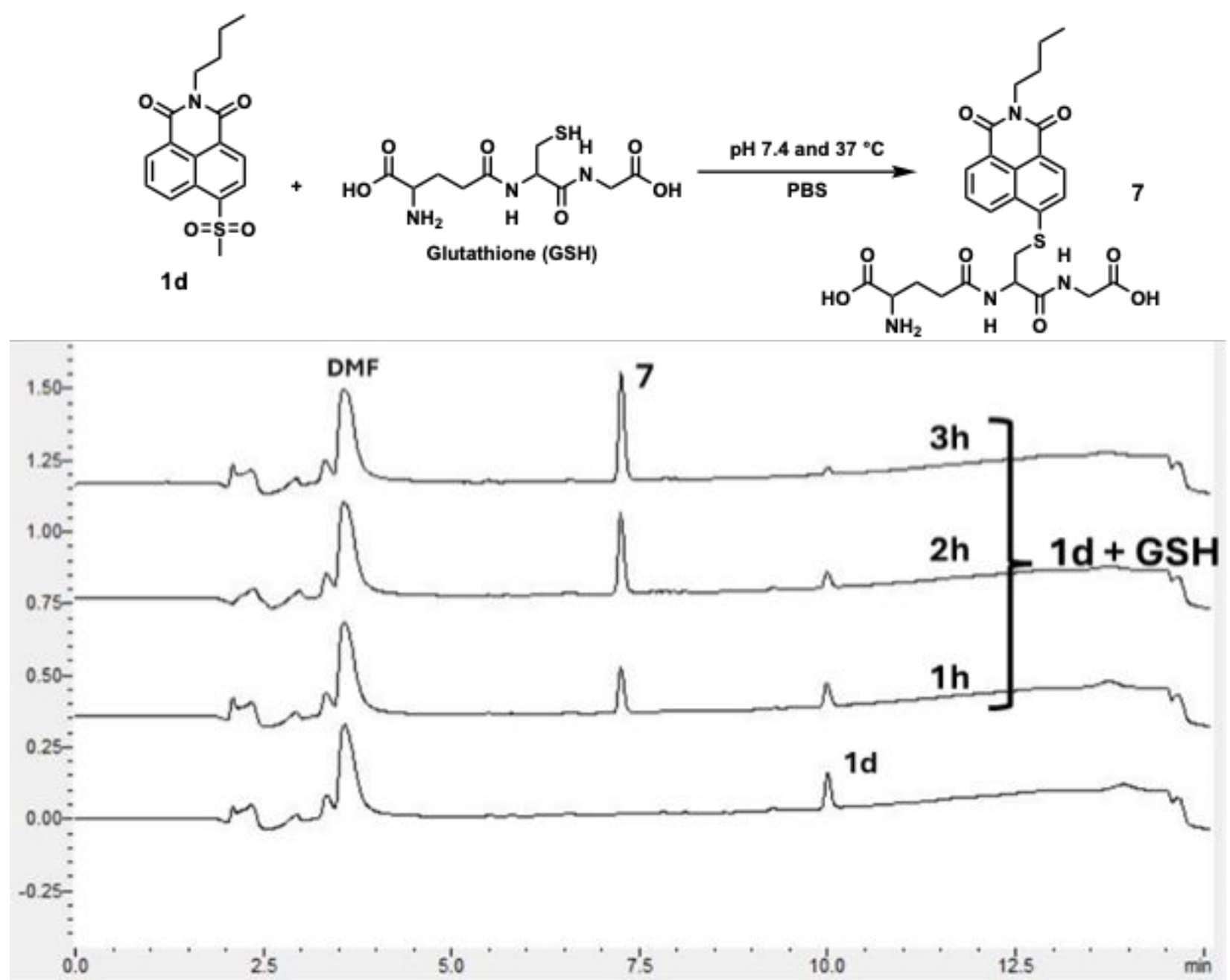

Figure S6. 100 µM probe **1d** incubated with 1 mM GSH in PBS:ACN (1:1) at pH 7.4 and 37 °C.

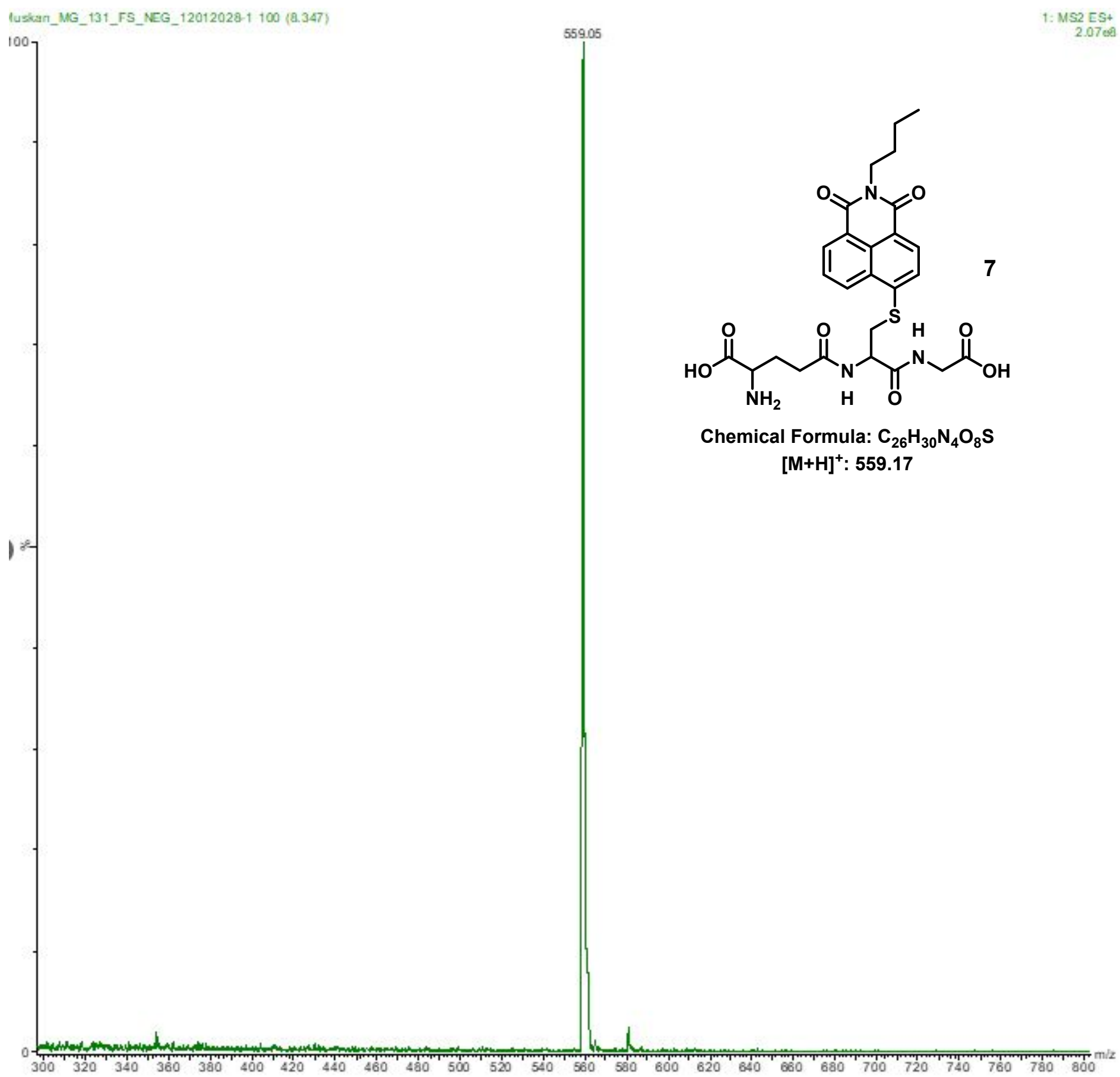

Figure S7. MS spectrum product **7**

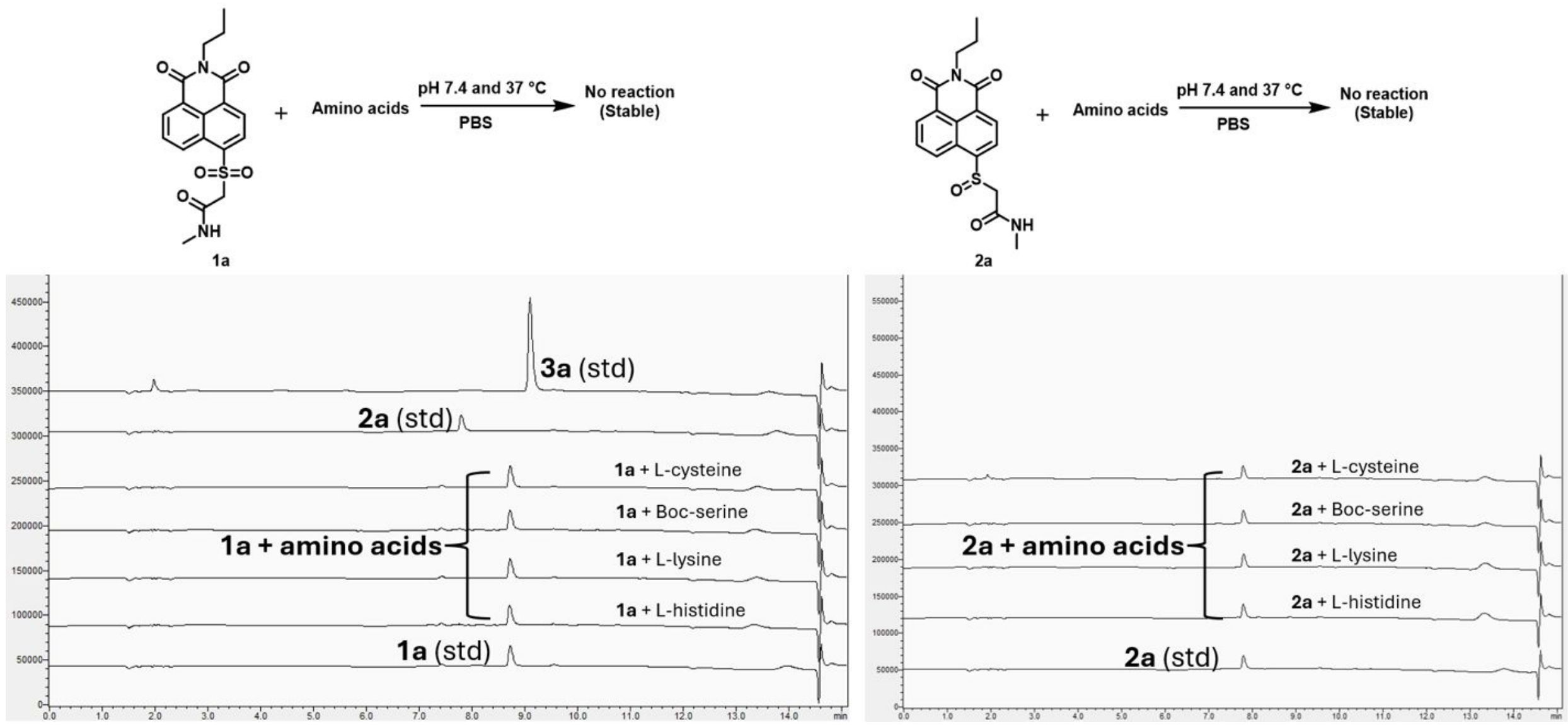

Figure S8. Probe **1a/2a** reaction with various amino acids. 10 μM probe **1a** or **2a** was incubated with 10 μM of various amino acids in PBS:ACN (1:1) at pH 7.4 and 37 °C.

9. NMR Spectra

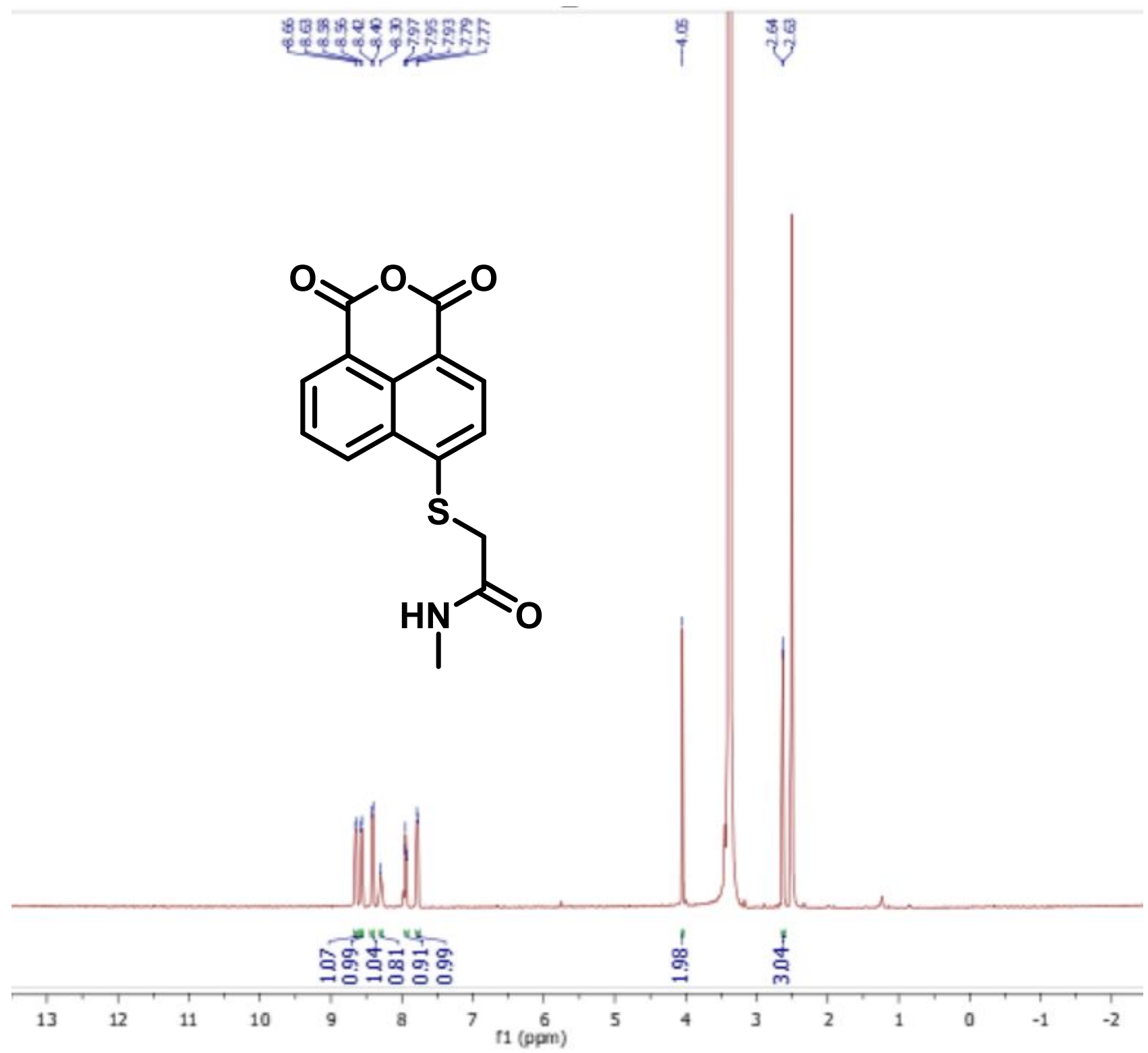

Figure S9. ^{1}H NMR of compound **6a**.

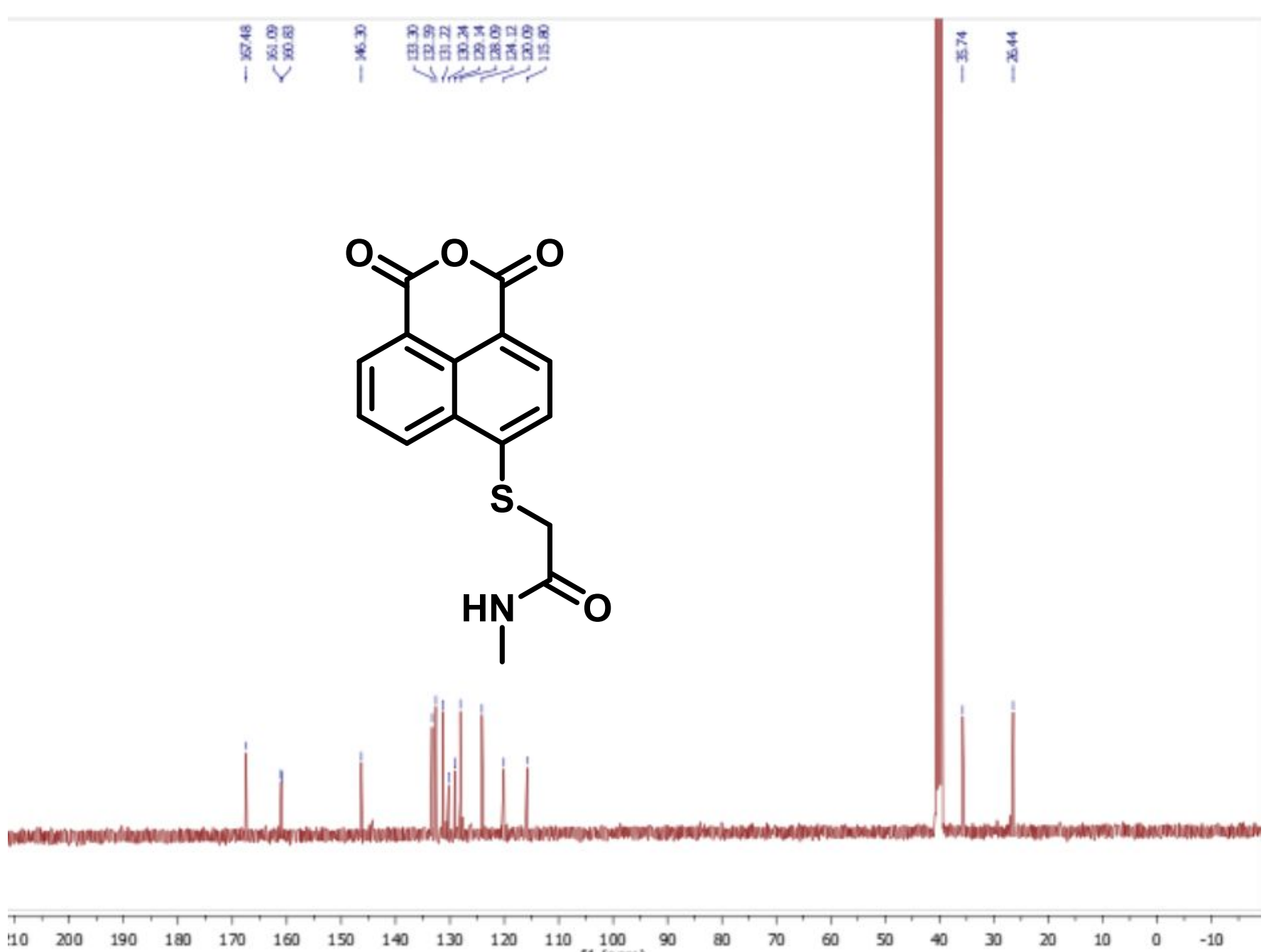

Figure S10. ^{13}C NMR of compound **6a**.

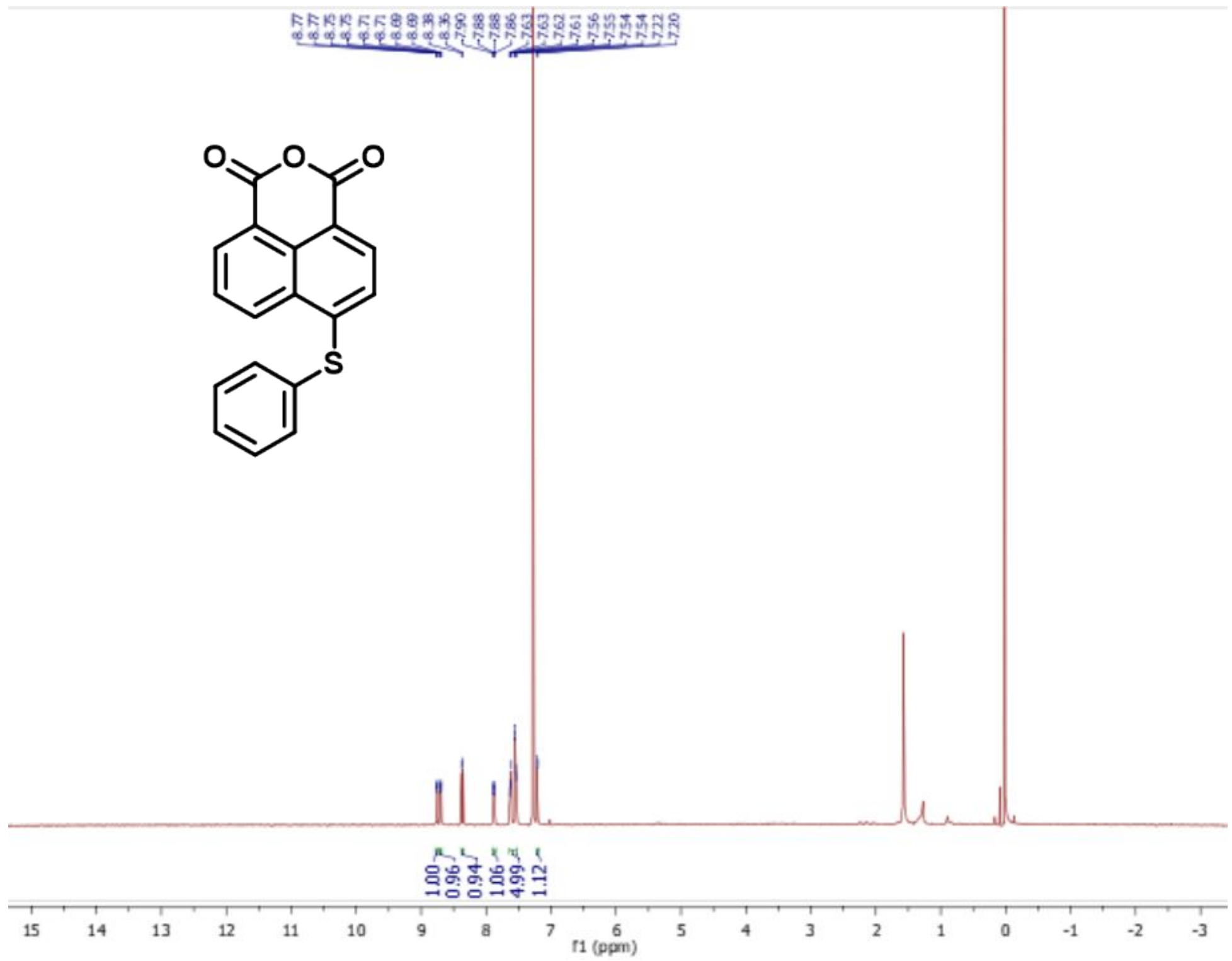

Figure S11. ^{1}H NMR of compound **6b**.

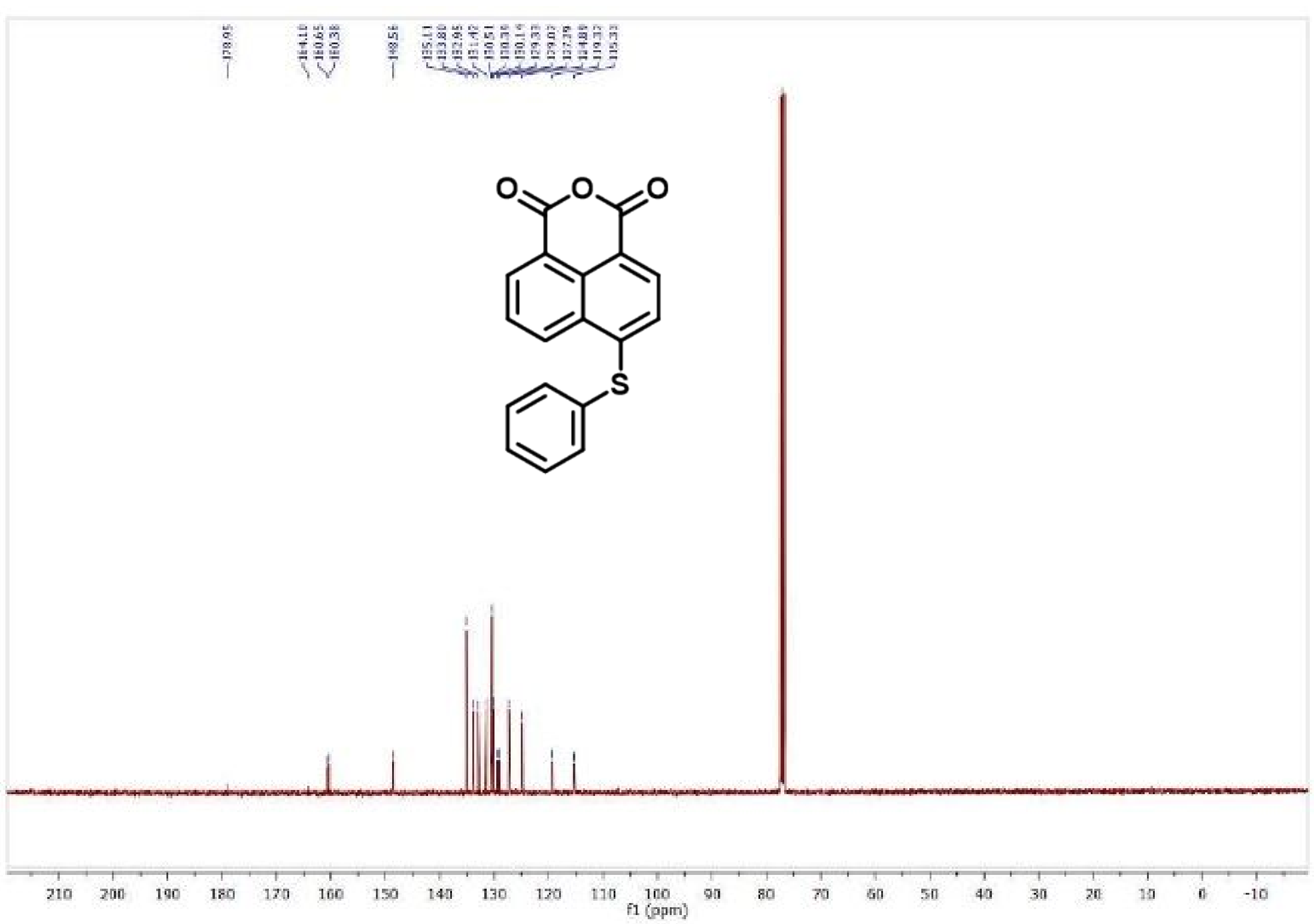

Figure S12. ^{13}C NMR of compound **6b**.

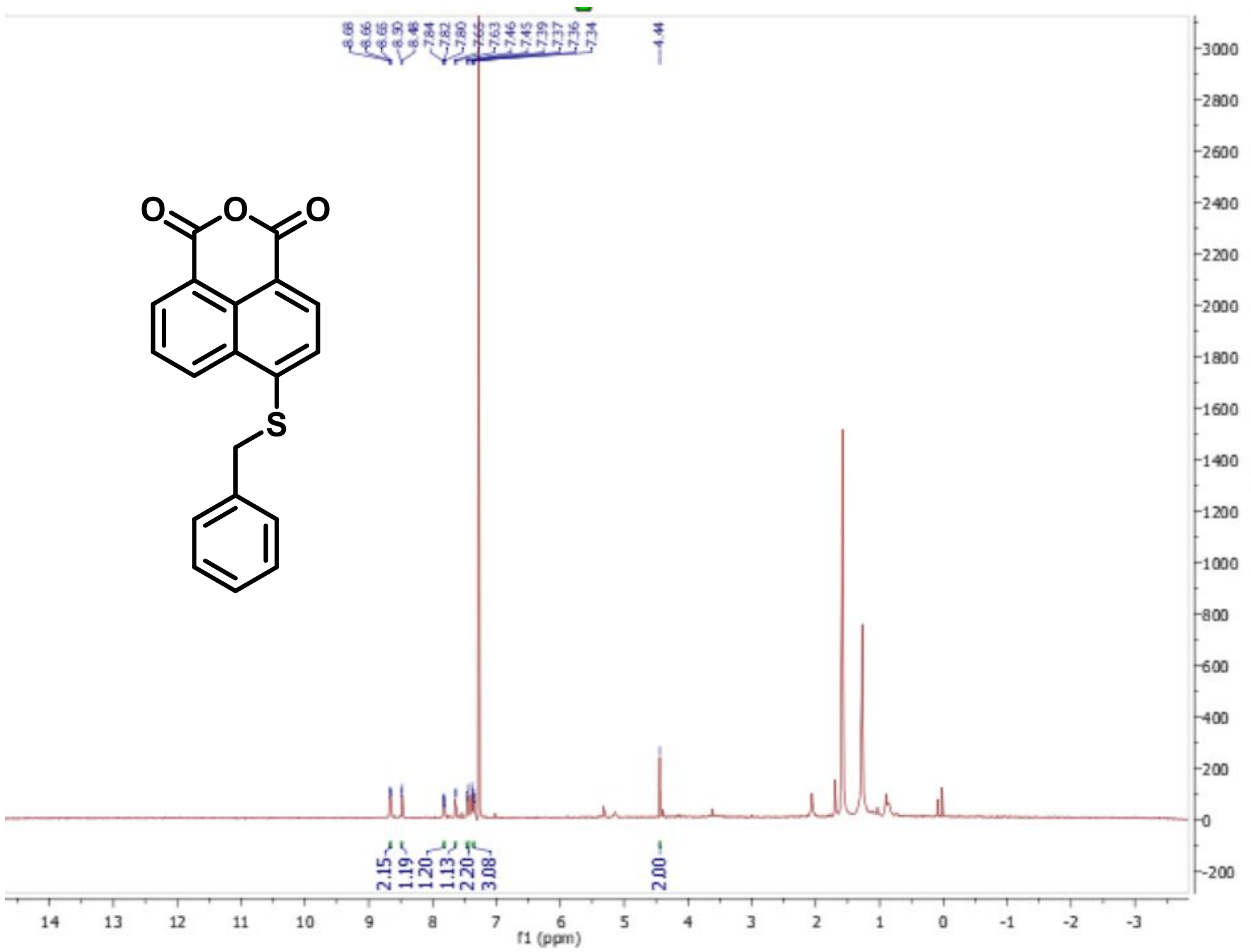

Figure S13. ^{1}H NMR of compound **6c**.

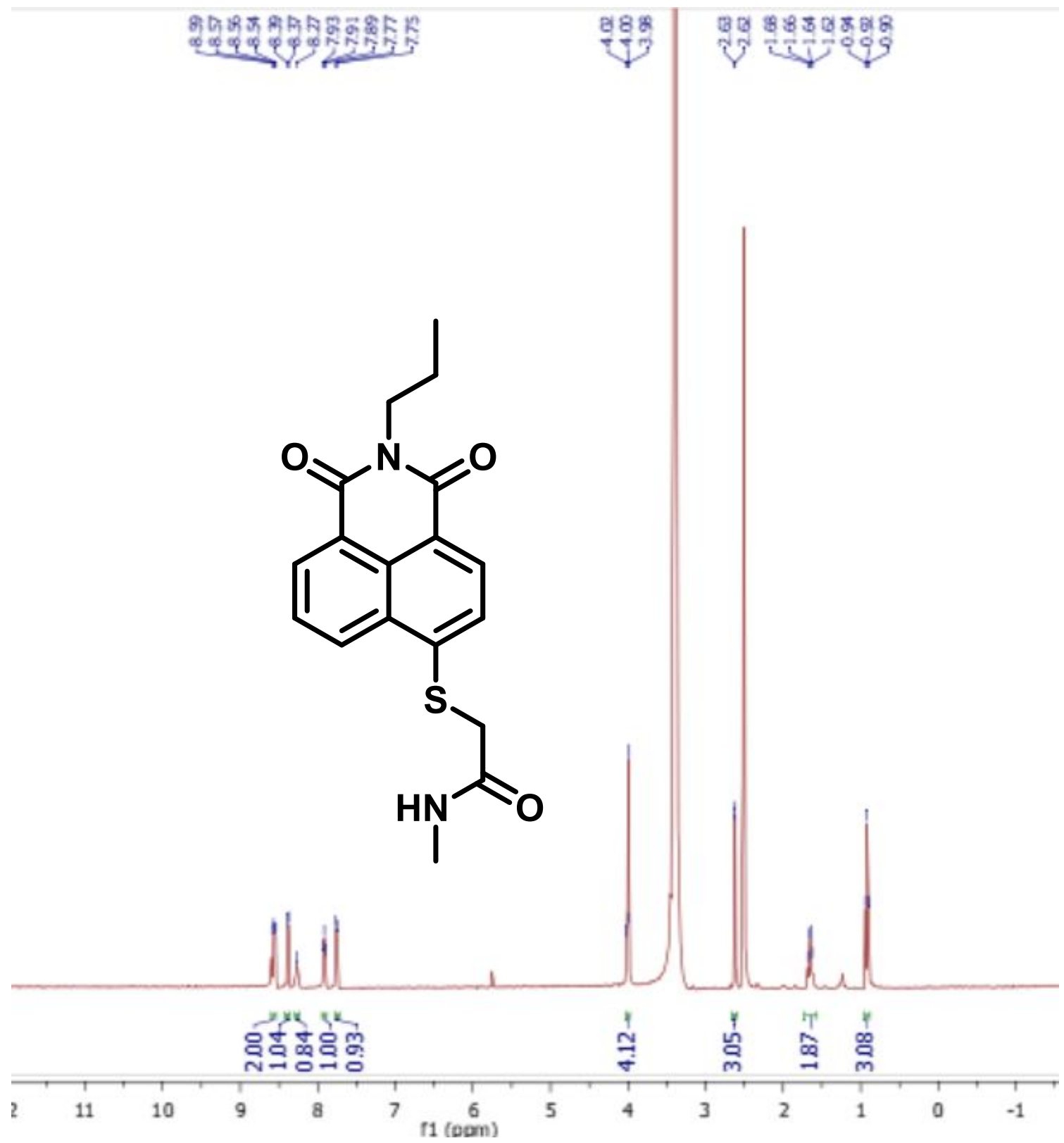

Figure S14. ^{1}H NMR of compound **3a**.

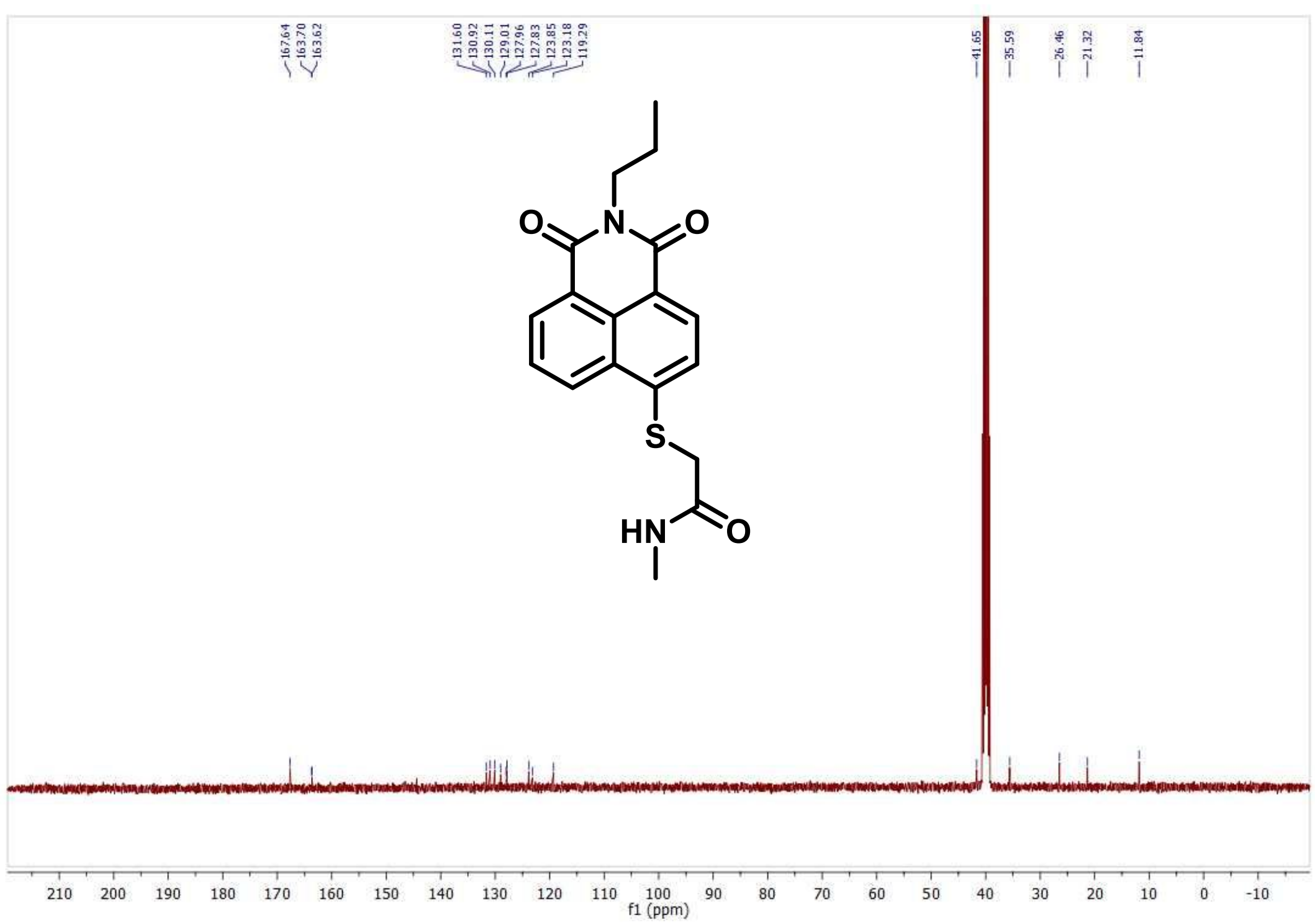

Figure S15. ^{13}C NMR of compound **3a**.

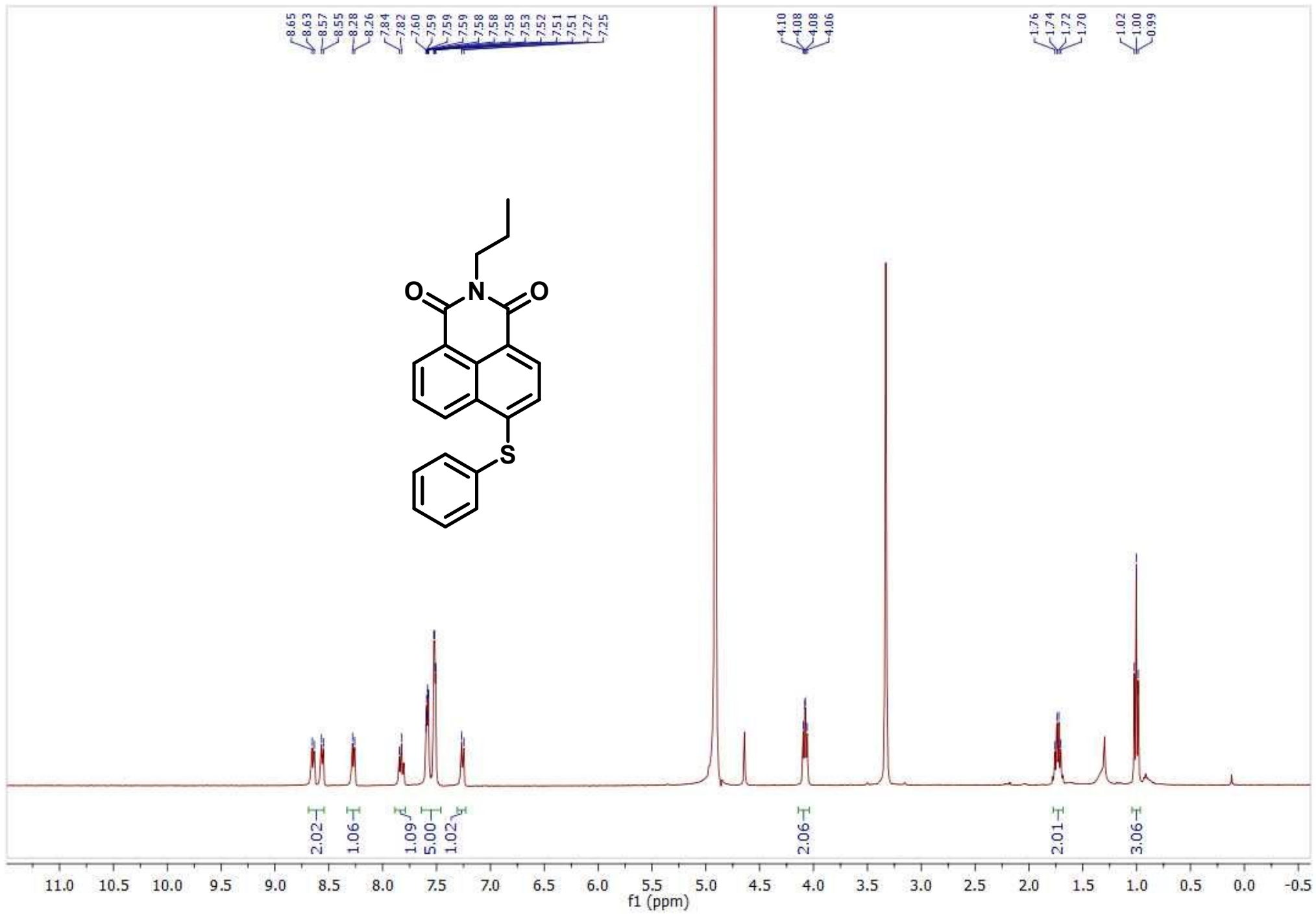

Figure S16. ^{1}H NMR of compound **3b**.

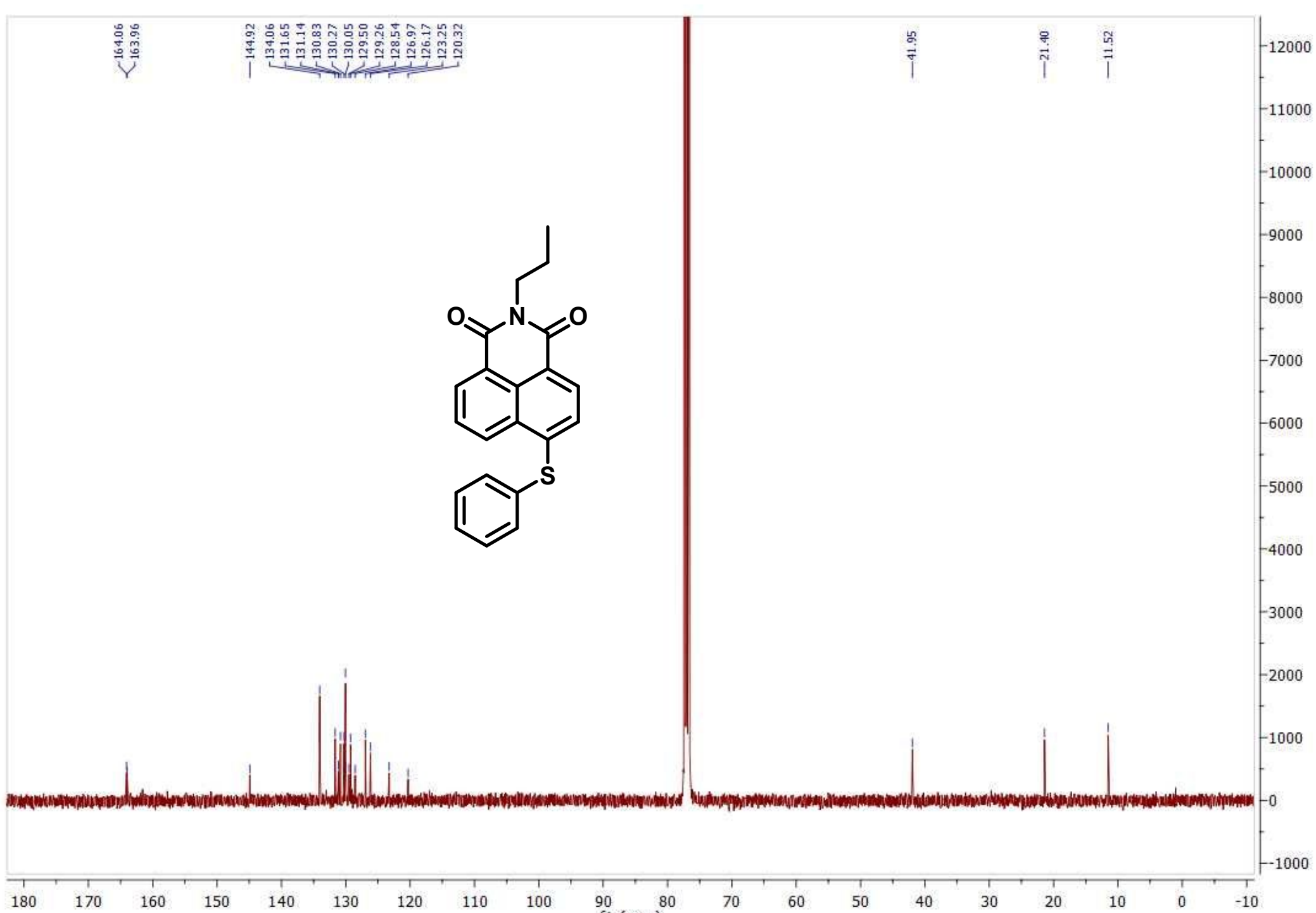

Figure S17. ^{13}C NMR of compound **3b**.

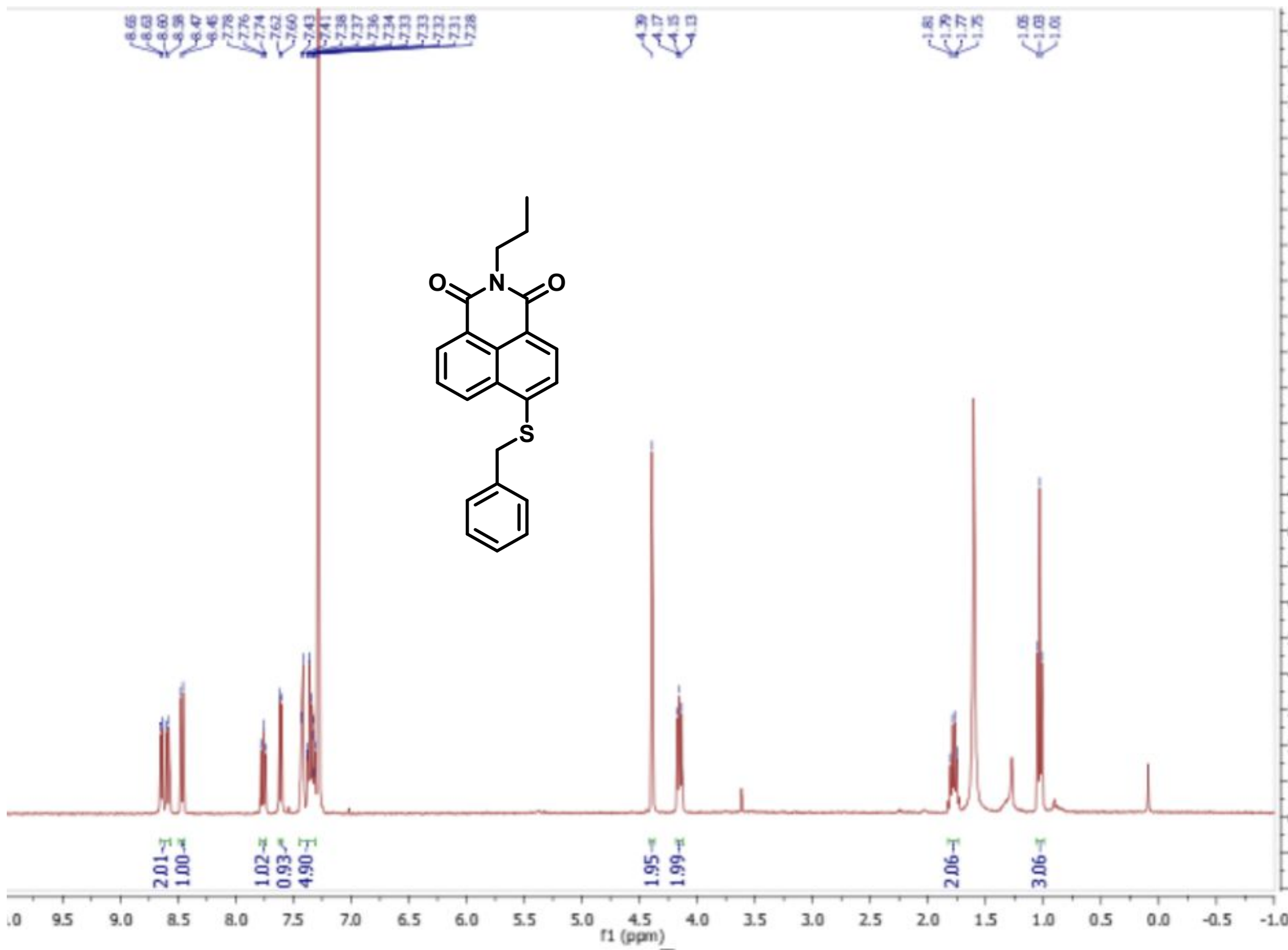

Figure S18. ^{1}H NMR of compound **3c**.

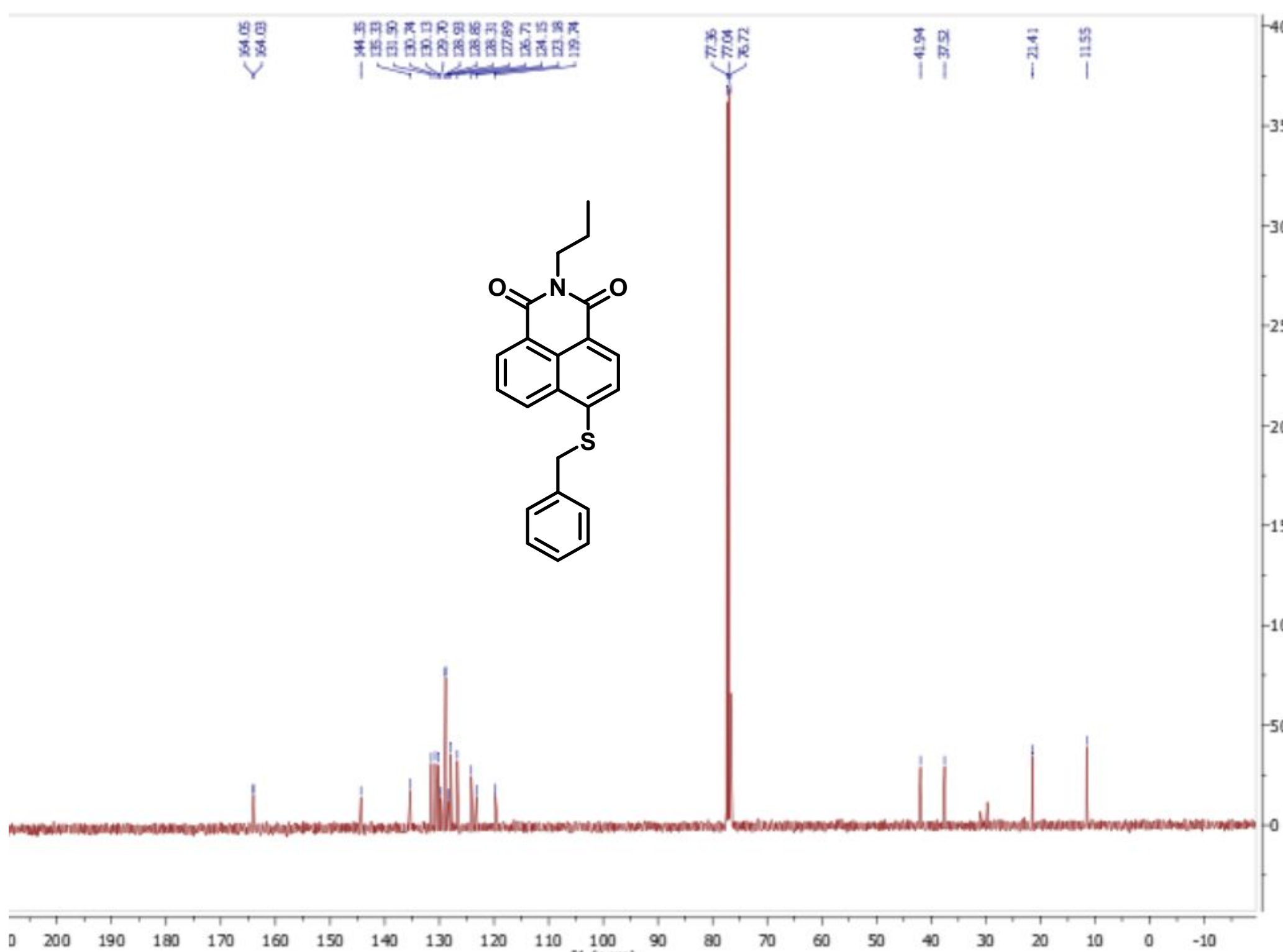

Figure S19. ^{13}C NMR of compound **3c**.

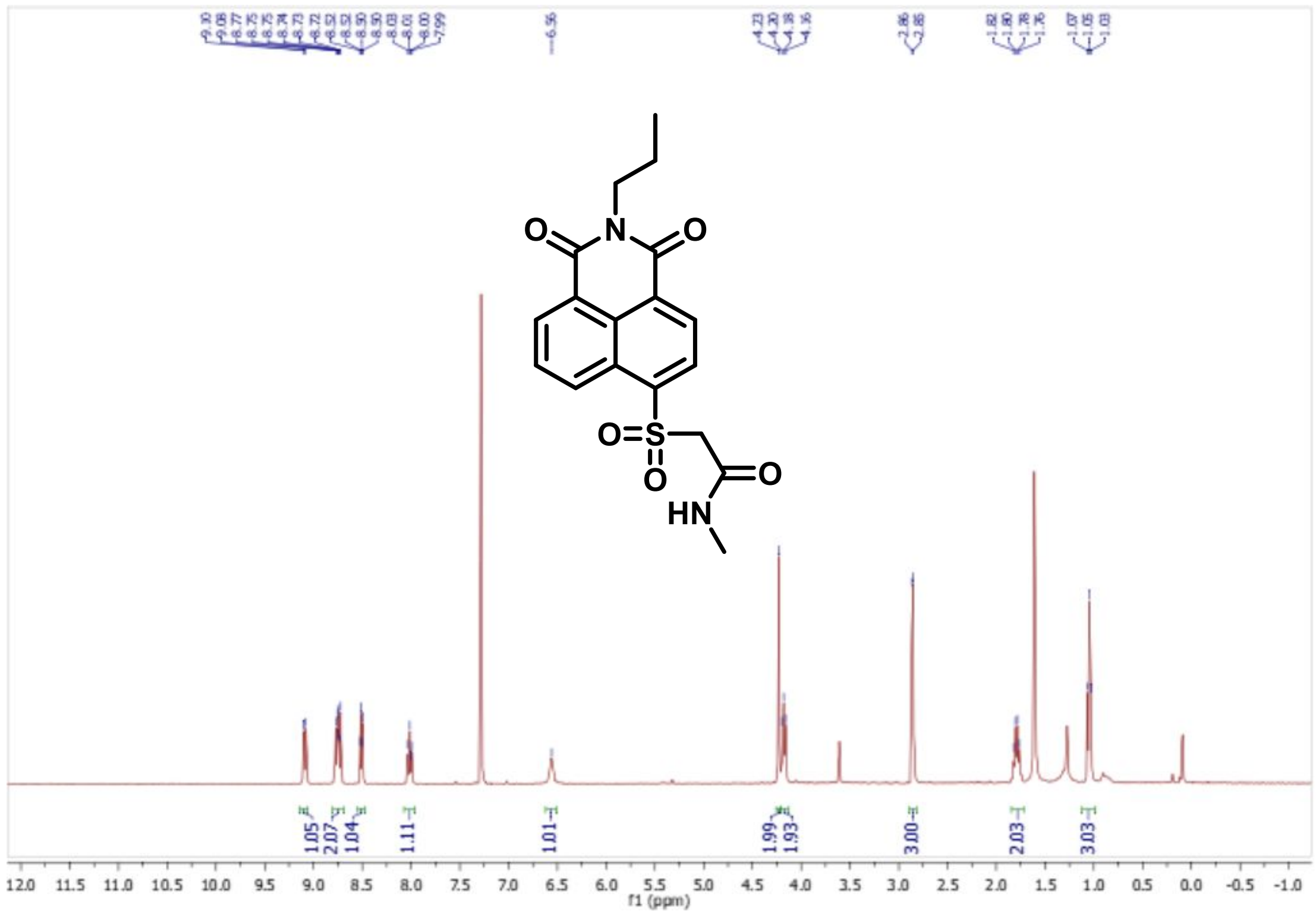

Figure S20. ^{1}H NMR of compound **1a**.

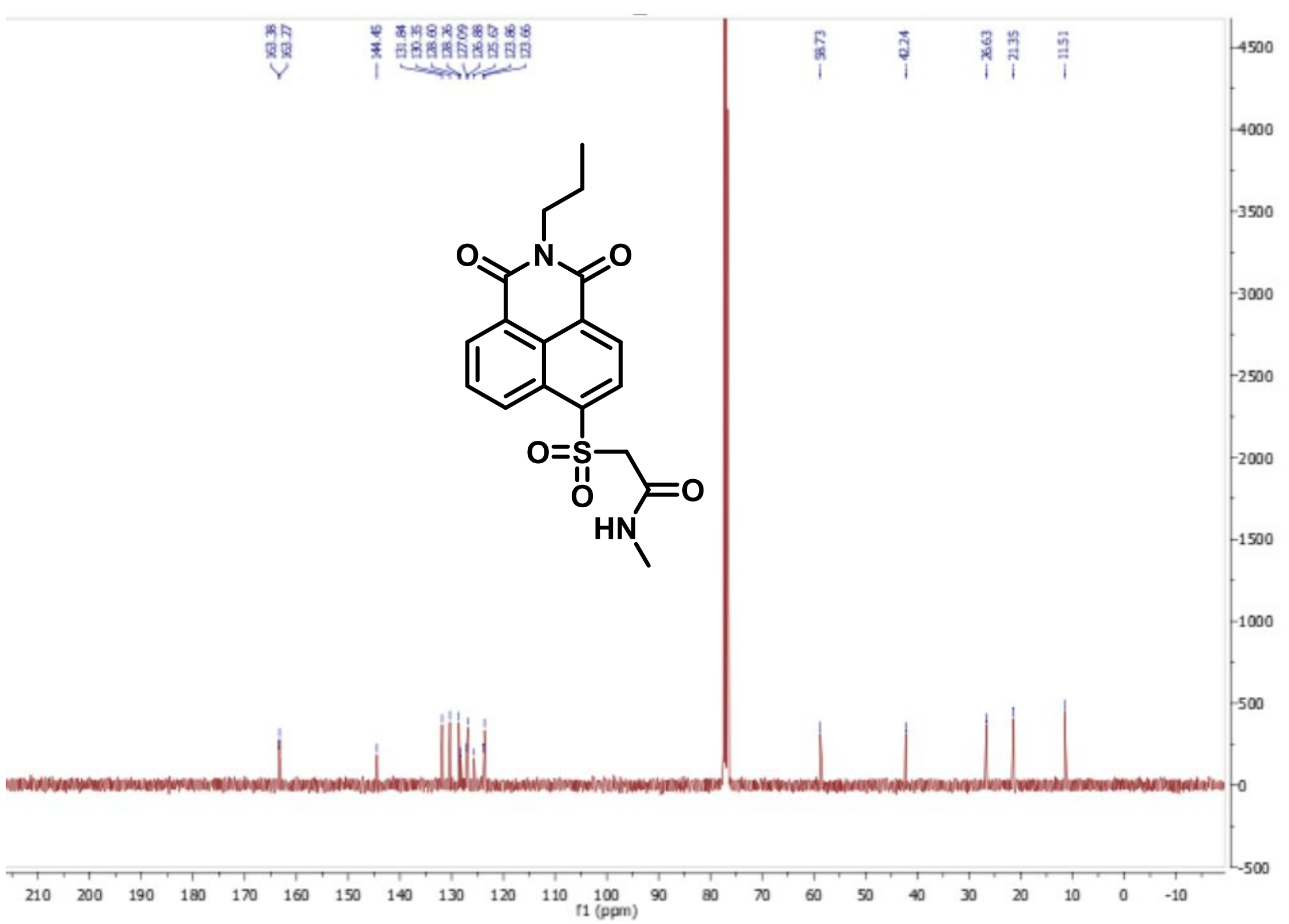

Figure S21. ^{13}C NMR of compound **1a**.

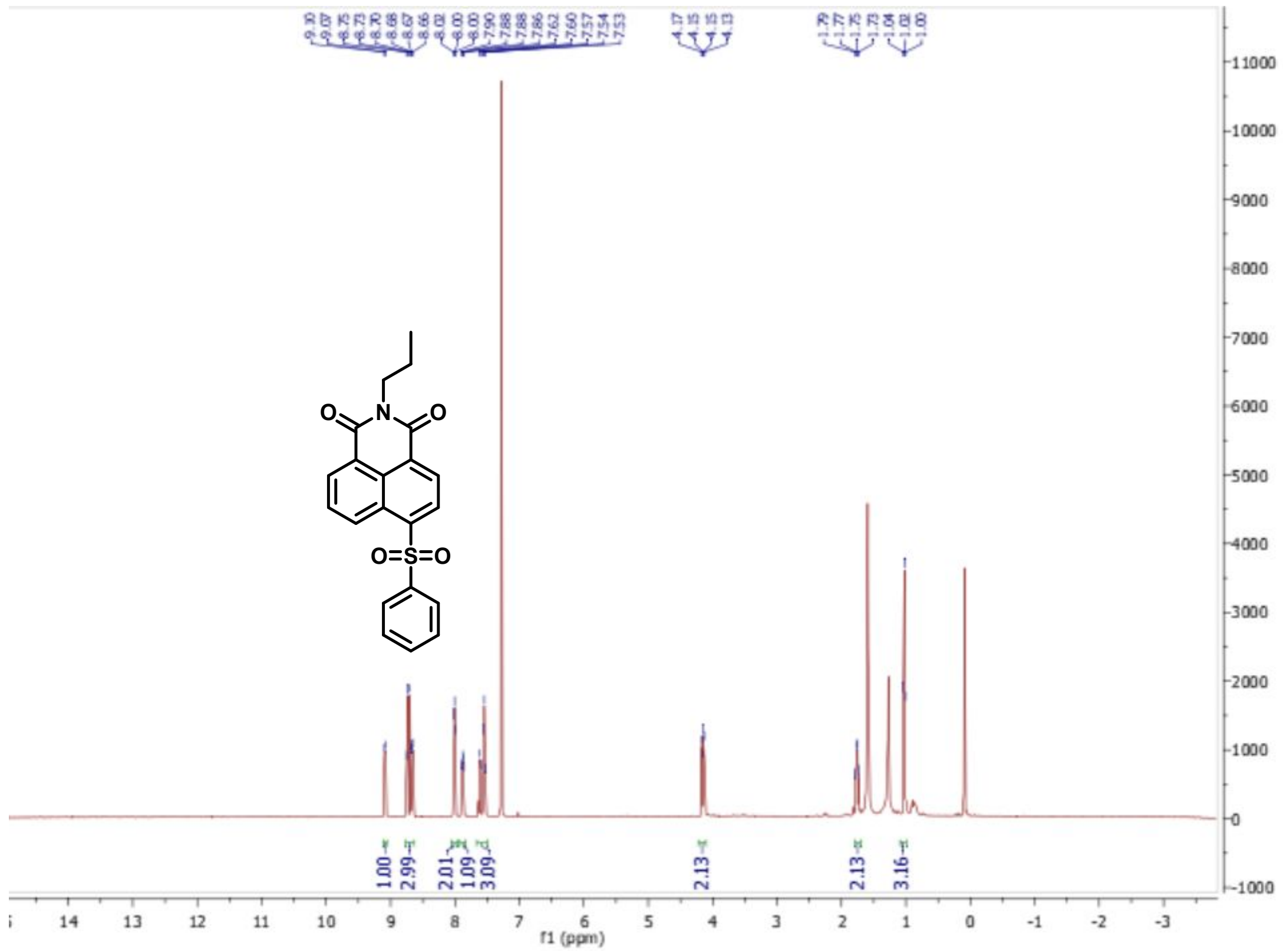

Figure S22. ^{1}H NMR of compound **1b**.

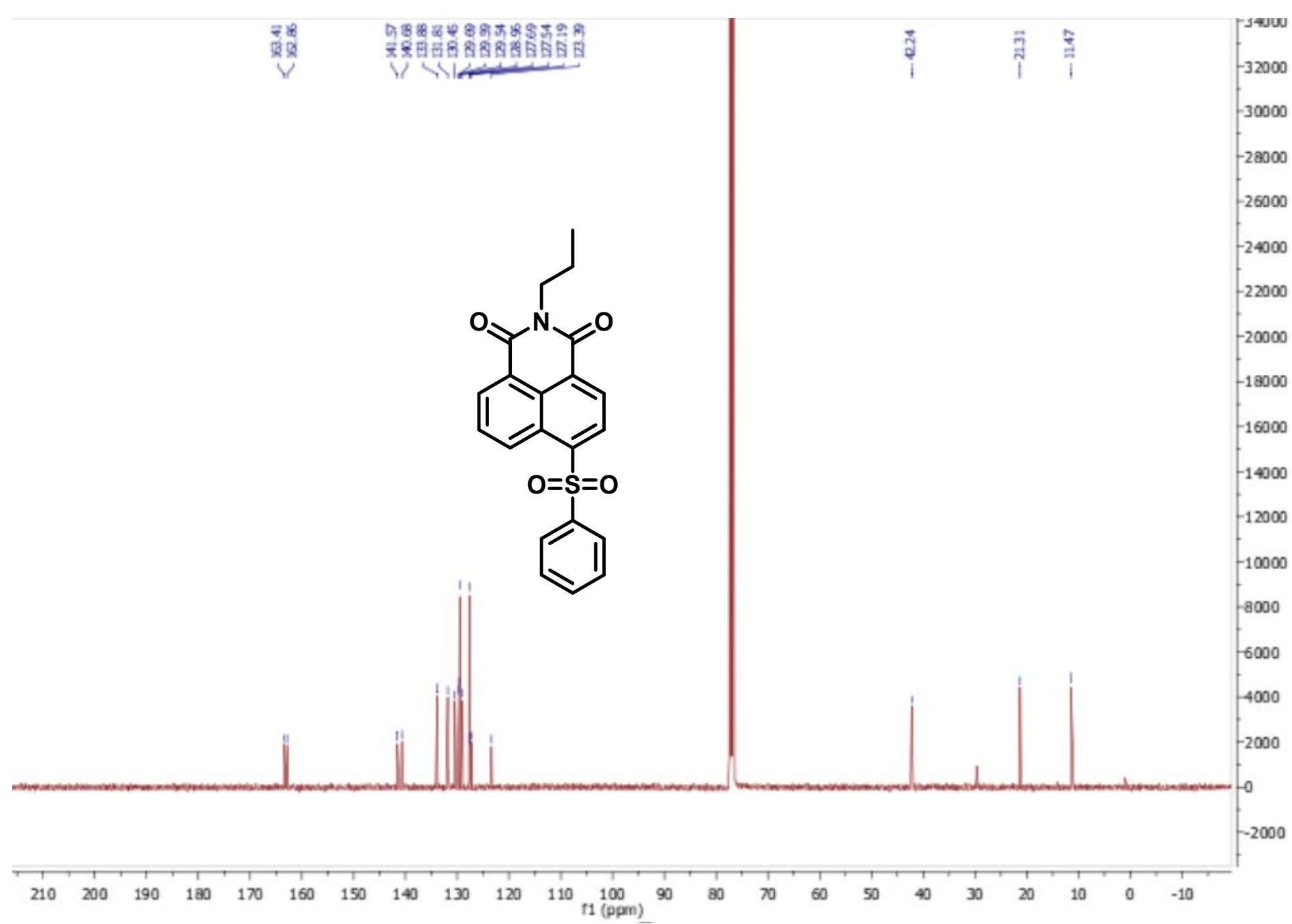

Figure S23. ^{13}C NMR of compound **1b**.

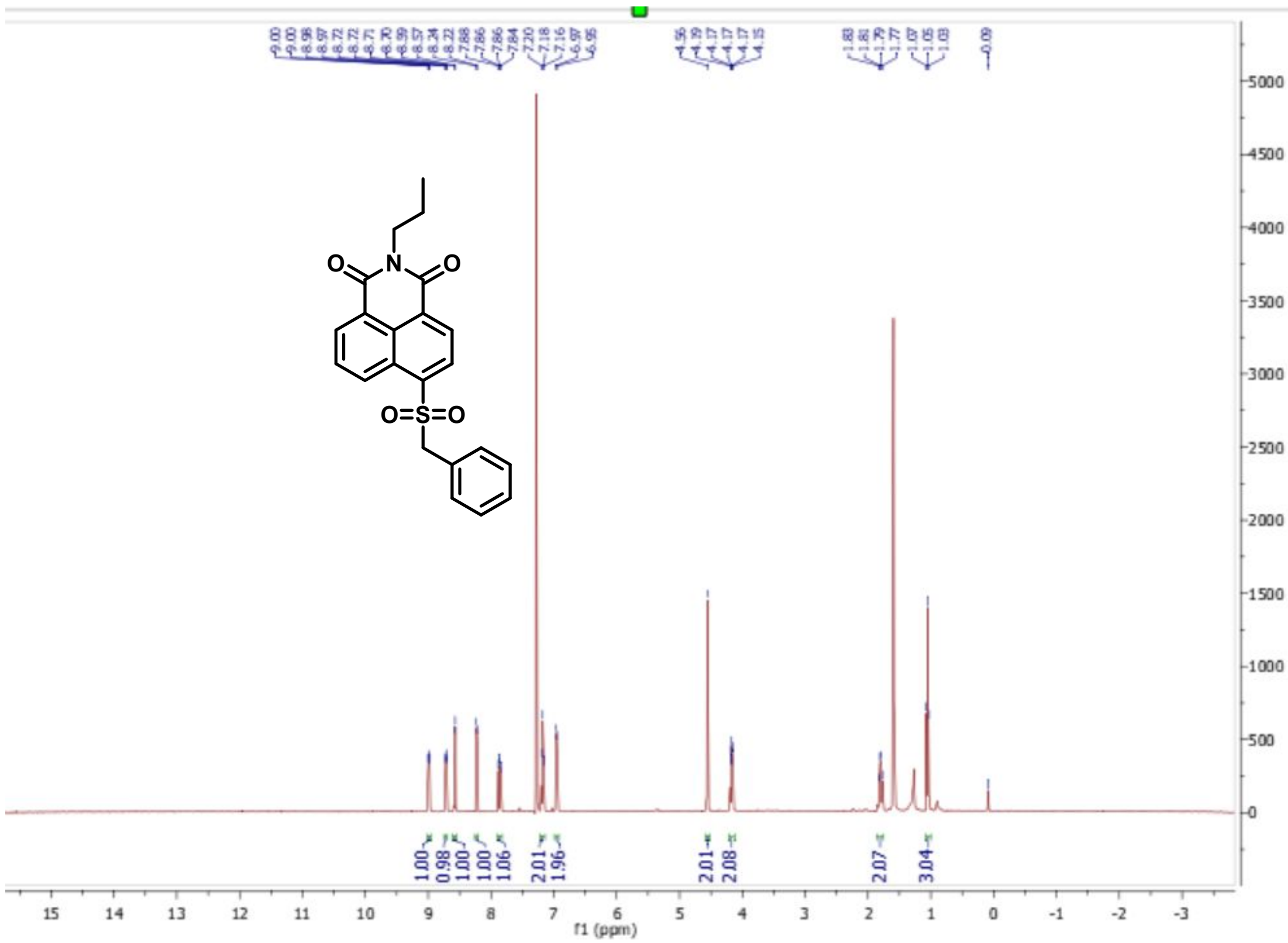

Figure S24. ^{1}H NMR of compound **1c**.

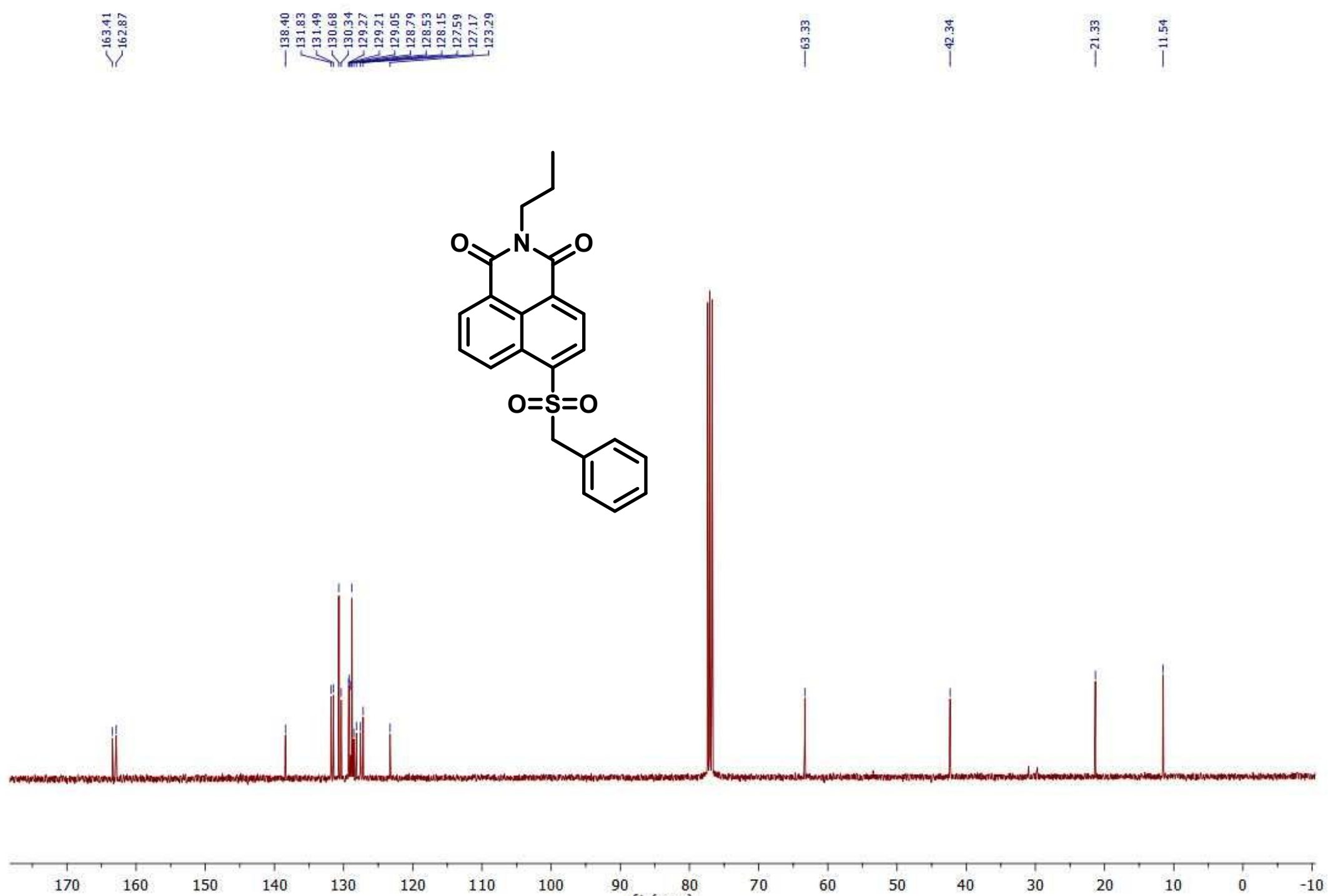

Figure S25. ^{13}C NMR of compound **1c**.

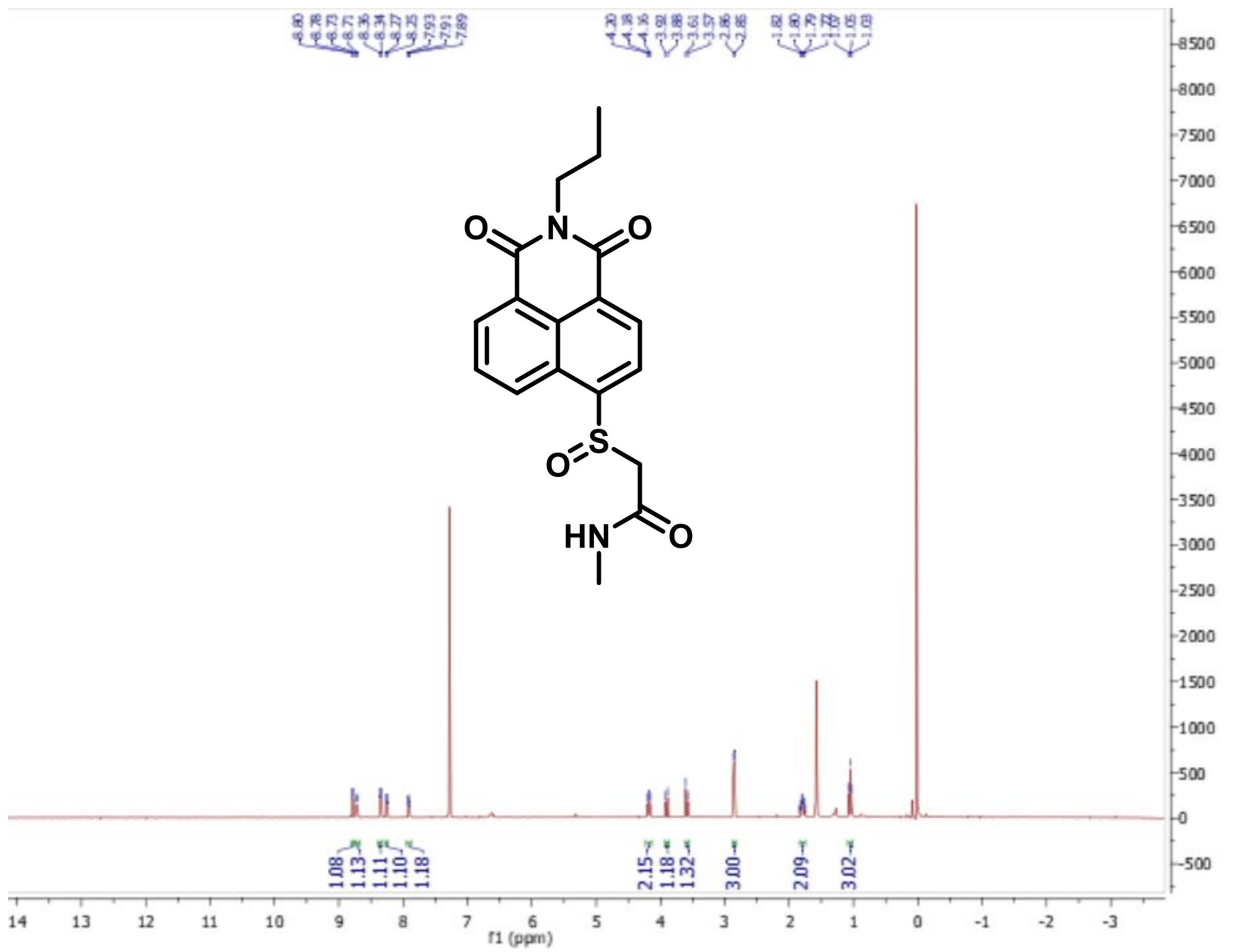

Figure S26. ^{1}H NMR of compound **2a**.

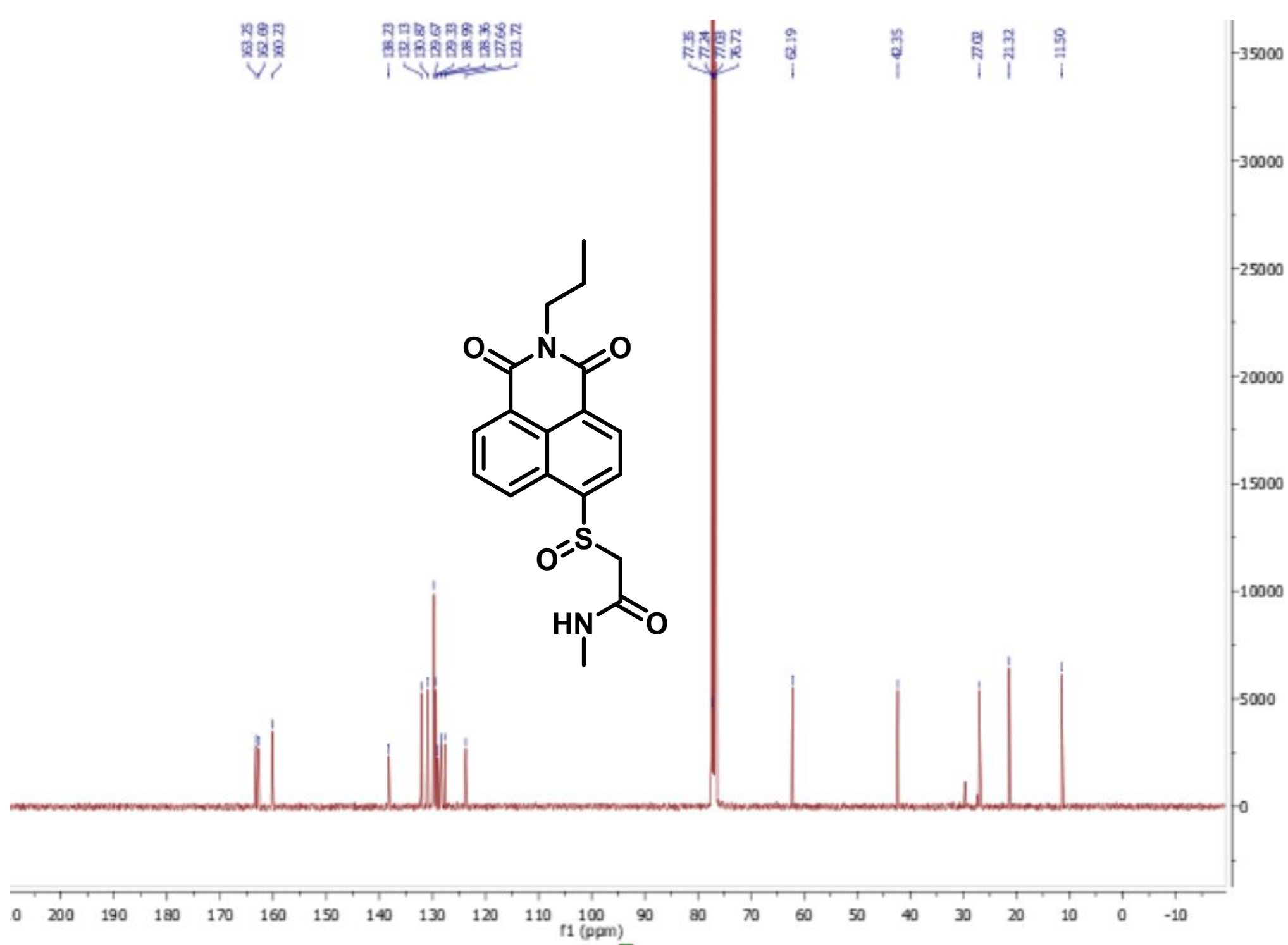

Figure S27. ^{13}C NMR of compound **2a**.

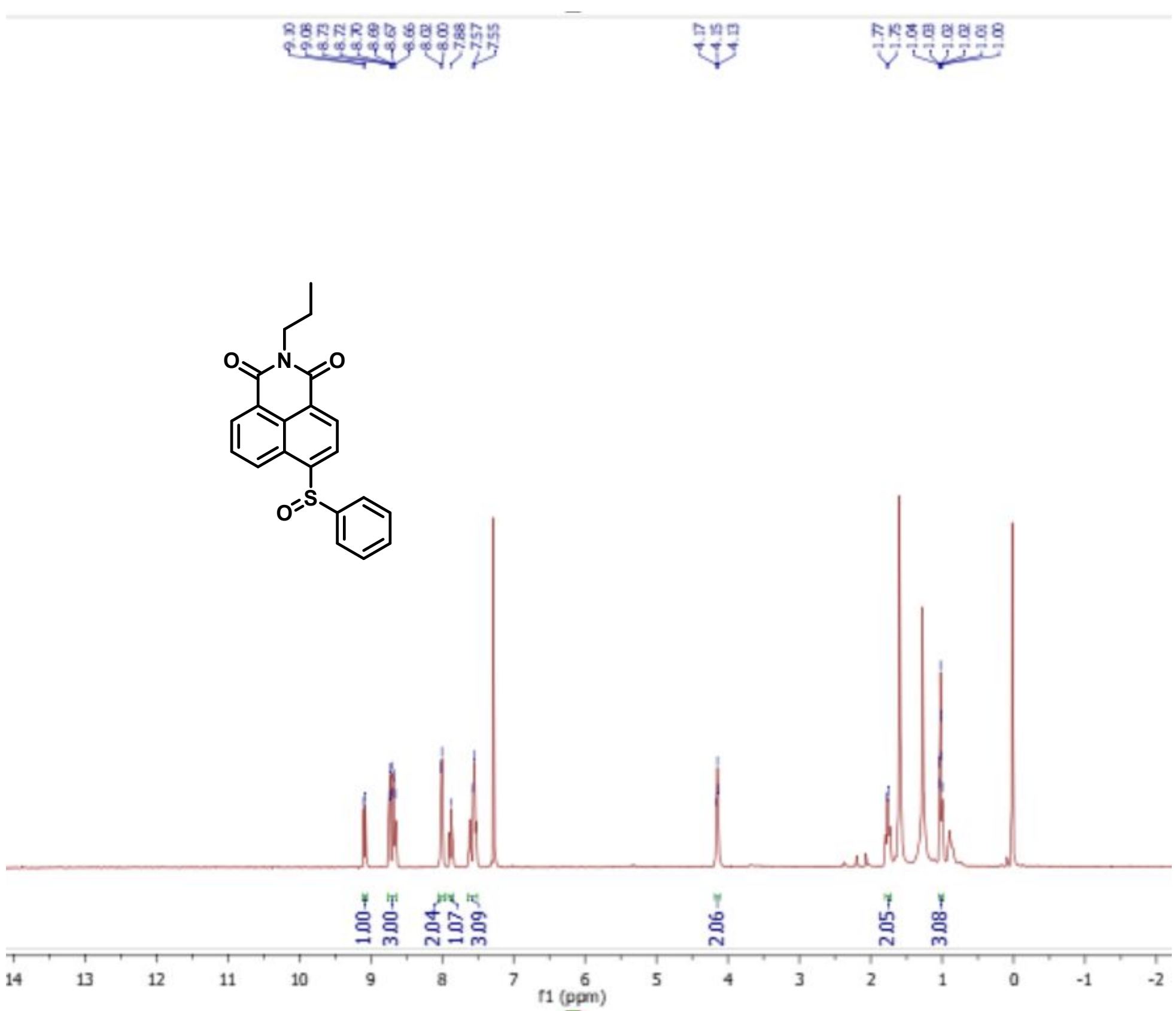

Figure S28. ^{1}H NMR of compound **2b**.

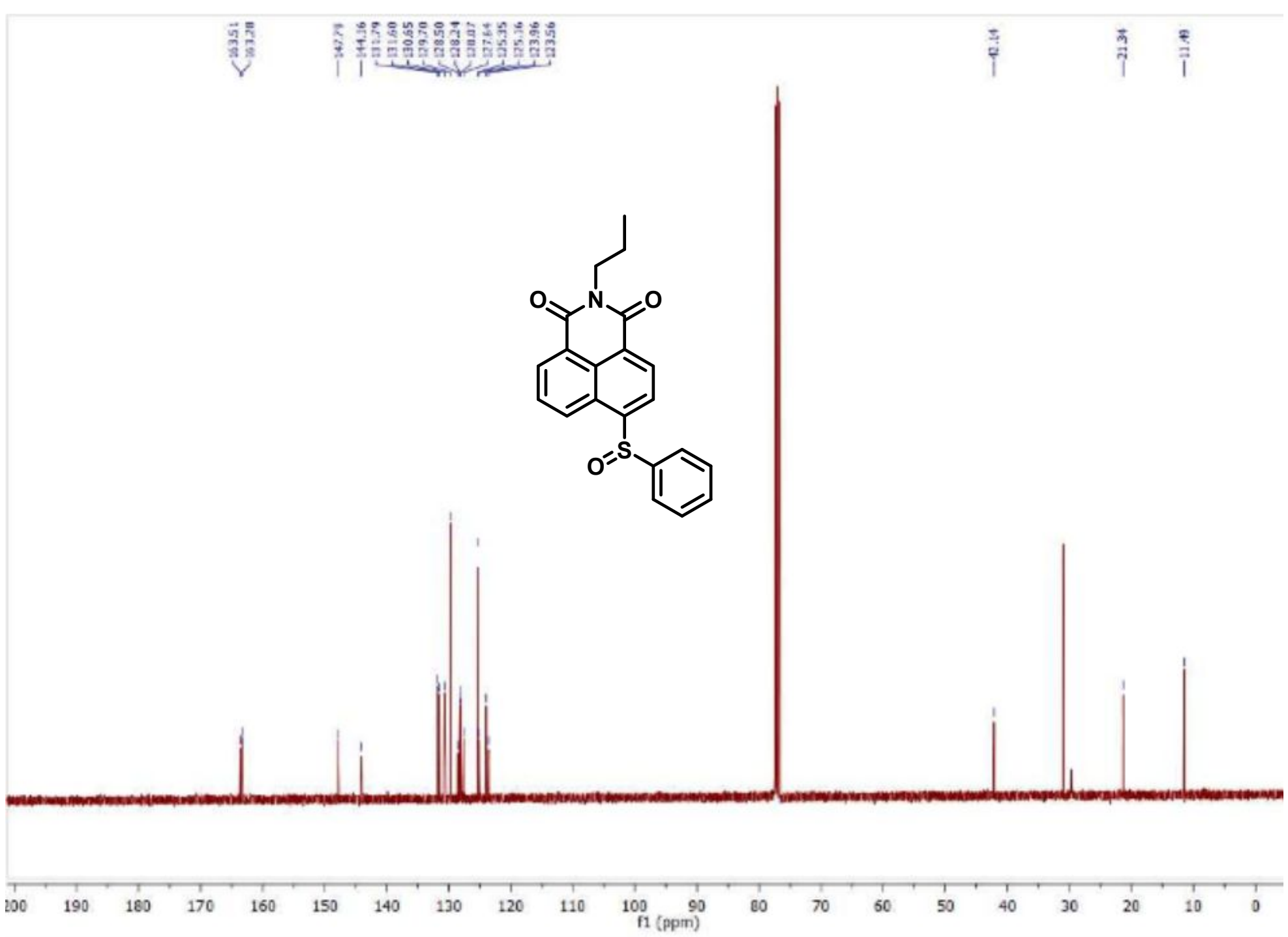

Figure S29. ^{13}C NMR of compound **2b**.

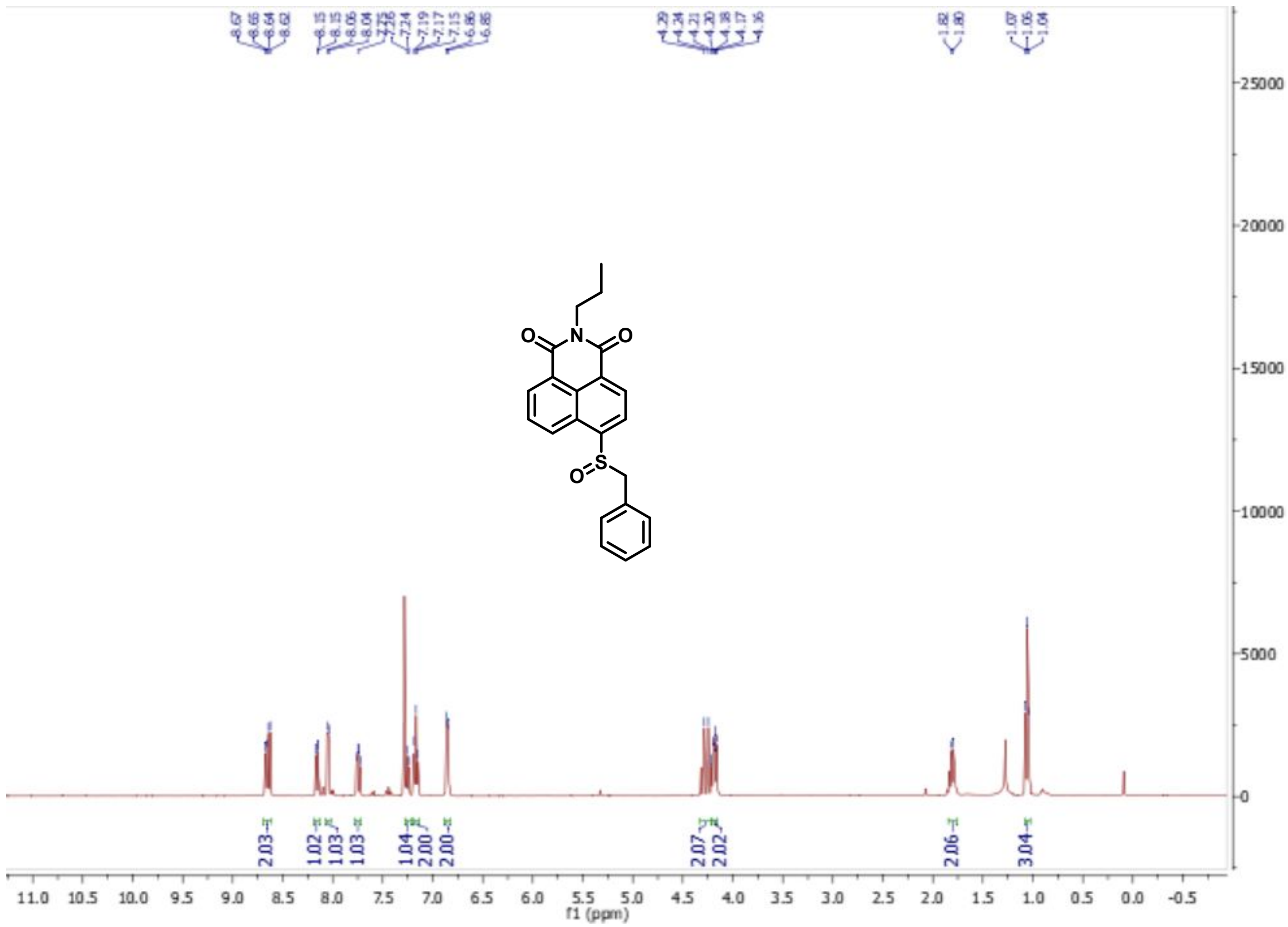

Figure S30. ¹H NMR of compound **2c**.

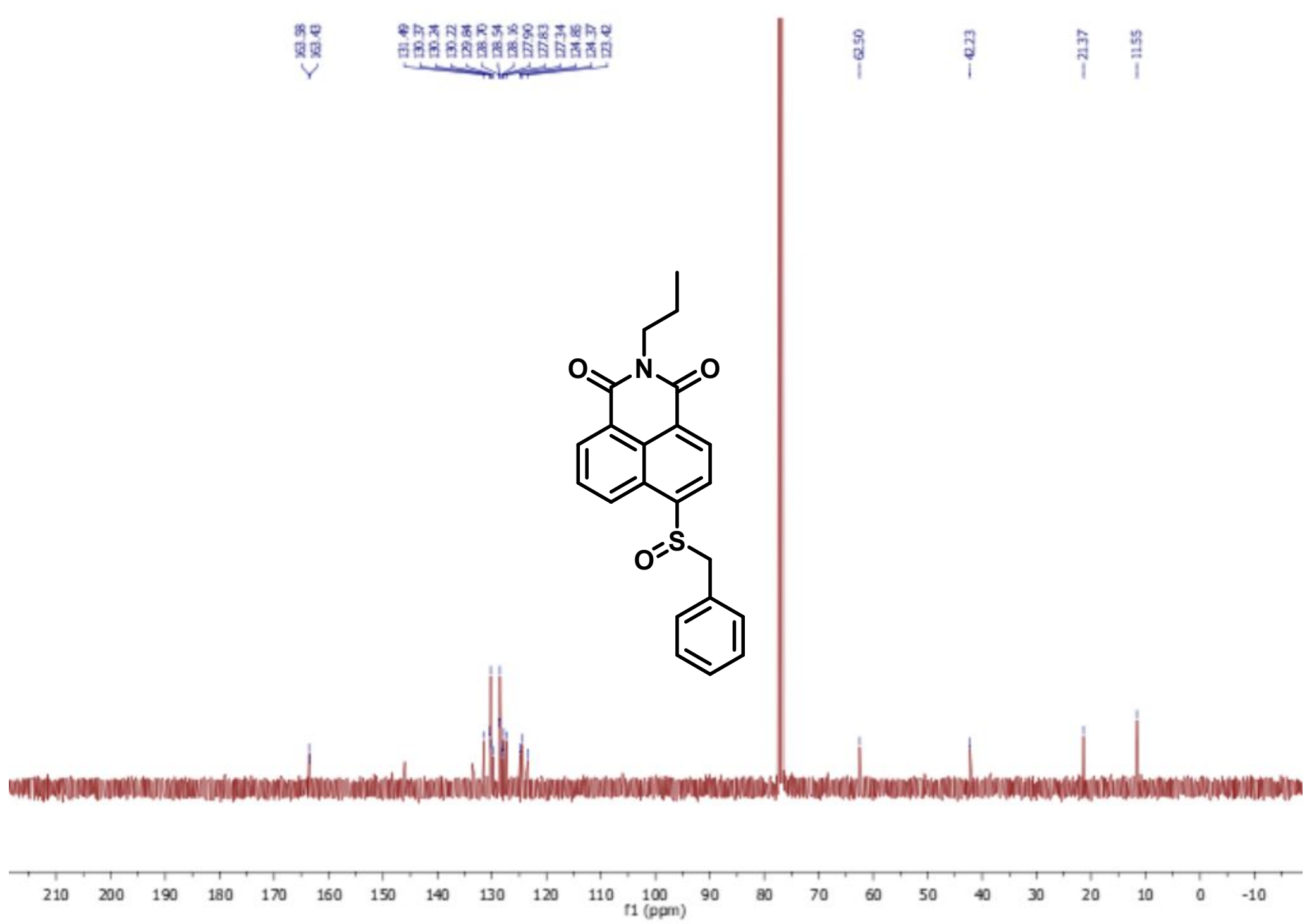

Figure S31. ¹H NMR of compound **2c**.

10. References

(1) Bruemmer, K. J.; Merrikhihaghi, S.; Lollar, C. T.; Morris, S. N.; Bauer, J. H.; Lippert, A. R. ^{19}F magnetic resonance probes for live-cell detection of peroxynitrite using an oxidative decarbonylation reaction. *Chem Commun (Camb)* **2014**, *50* (82), 12311-12314.
(2) Heller, C. A.; Henry, R. A.; McLaughlin, B. A.; Bliss, D. E. Fluorescence spectra and quantum yields. Quinine, uranine, 9,10-diphenylanthracene, and 9,10-bis(phenylethynyl)anthracenes. *Journal of Chemical & Engineering Data* **1974**, *19* (3), 214-219.
(3) Hoang, V. T.; Stępniewski, G.; Czarnecka, K. H.; Kasztelanic, R.; Long, V. C.; Xuan, K. D.; Shao, L.; Śmietana, M.; Buczyński, R. Optical Properties of Buffers and Cell Culture Media for Optofluidic and Sensing Applications. *Applied Sciences* **2019**, *9* (6), 1145.
(4) Chen, R.; Lu, H.; Liu, C.; Kong, Q. Novel chemosensors for detection of glutathione by reduction or substitution of naphthalimide derivatives containing sulfoxide or sulfone substituents. *Bioorg Med Chem Lett* **2015**, *25* (1), 59-61.
(5) Ott, I.; Xu, Y.; Liu, J.; Kokoschka, M.; Harlos, M.; Sheldrick, W. S.; Qian, X. Sulfur-substituted naphthalimides as photoactivatable anticancer agents: DNA interaction, fluorescence imaging, and phototoxic effects in cultured tumor cells. *Bioorg Med Chem* **2008**, *16* (15), 7107-7116.
(6) Lan, H.; Li, Q.; Li, Y.; Xiao, S.; Li, Y.; Yan, X.; Yuan, S.; Zhu, P. Solvent-controlled tautomerism of malononitrile-naphthalimide via intramolecular proton transfer. *Dyes and Pigments* **2018**, *155*, 121-125.
